# Supplementary material for: Intravenously injected hPSC-derived pericytes for Alzheimer disease: Neuroprotection and vascular repair via extracellular vesicles
Source: Mol Ther. 2025 Aug 19;33(11):5681–703. doi: 10.1016/j.ymthe.2025.08.024 (PMC12628151; doi:10.1016/j.ymthe.2025.08.024)
Supplement: Document S1. Tables S1–S7 and Figures S1–S10 [file mmc1.pdf]

YMTHE, Volume *33*

Supplemental Information

Intravenously injected hPSC-derived pericytes for Alzheimer disease: Neuroprotection and vascular repair via extracellular vesicles

Ying Liu, Zhiyuan Ning, Qingyuan Dai, Xinkai Zhang, Yibin Xiao, Zhan Zhang, Daji Guo, Junhua Chen, Yi Li, Weiqiang Li, Songhua Xiao, and Yamei Tang

Table S1. Top 20 miRNA in the EVs

ID	miRNA	Average_UMI
MIMAT0002177	hsa-miR-486-5p	100378.6667
MIMAT0016887	hsa-miR-4325	64528.66667
MIMAT0026613	hsa-miR-510-3p	9010
MIMAT0027477	hsa-miR-6788-3p	7454.666667
MIMAT0000423	hsa-miR-125b-5p	5873.666667
MIMAT0022947	hsa-miR-1238-5p	5082
MIMAT0000062	hsa-let-7a-5p	4658.333333
MIMAT0031014	hsa-miR-8087	4114.333333
MIMAT0003278	hsa-miR-610	3103
MIMAT0000076	hsa-miR-21-5p	2316.333333
MIMAT0027494	hsa-miR-6797-5p	2192.666667
MIMAT0012735	hsa-miR-718	2152
MIMAT0000232	hsa-miR-199a-3p	2081.333333
MIMAT0004563	hsa-miR-199b-3p	2069.333333
MIMAT0000069	hsa-miR-16-5p	1978
MIMAT0000261	hsa-miR-183-5p	1932.666667
MIMAT0027647	hsa-miR-6873-3p	1837
MIMAT0027507	hsa-miR-6803-3p	1770.333333
MIMAT0015062	hsa-miR-3182	1684.666667
MIMAT0027384	hsa-miR-6741-3p	1564.333333

Table S2. Primers used for quantitative PCR

Gene	Direction	Sequences
U6-F	5’-3’	CTCGCTTCGGCAGCACA
U6-R	5’-3’	ACGCTTCACGAATTTGC
miR-486-5p-F	5’-3’	TCCTGTACTGAGCTGCCCCGAG
Igf1r-F	5’-3’	GTGGGGGCTCGTGTTTCTC
Igf1r-R	5’-3’	GATCACCGTGCAGTTTTCCA
PDGFRβ-F	5’-3’	AATGCTGAGCGACCACTCCATC
PDGFRβ-R	5’-3’	TCGGGTCATGTTCAAGTCCAGC
NG2-R	5’-3’	GAGGTCTTGGTGAACTTCACCC
NG2-F	5’-3’	GACAGTAGGAGACCGATGGTGT
Human ALU-F	5’-3’	CAT GGT GAA ACC CCG TCT CTA
Human ALU-R	5’-3’	GCC TCA GCC TCC CGA GTA G
Mouse Gapdh-F	5’-3’	CAT CAC TGC CAC CCA GAA GAC TG
Mouse Gapdh-R	5’-3’	ATG CCA GTG AGC TTC CCG TTC AG
Mmp19-F	5’-3’	AGG CAC TCA TGG CTC CTG TCT A
Mmp19-R	5’-3’	TGA GCA TCT CGG TCT CTT CCT C
Smo-F	5’-3’	GAG GCT ACT TCC TCA TCA GAG G
Smo-R	5’-3’	GCT GAA GGT GAT GAG CAC AAA GC
Gli1-F	5’-3’	CTC AAA CTG CCC AGC TTA ACC C
Gli1-R	5’-3’	TGC GGC TGA CTG TGT AAG CAG A
Dock3-F	5’-3’	CTT CTC CAT CGT CAA GAC CAG C
Dock3-R	5’-3’	GAT GGT GAG GAG AGT CTG TAG G
Foxo1-F	5’-3’	CTA CGA GTG GAT GGT GAA GAG C
Foxo1-R	5’-3’	CCA GTT CCT TCA TTC TGC ACT CG
Smad1-F	5’-3’	CTG AAG CCT CTG GAA TGC TGT G
Smad1-R	5’-3’	CAG AAG GCT GTG CTG AGG ATT G
Smad4-F	5’-3’	CAG CCA TAG TGA AGG ACT GTT GC
Smad4-R	5’-3’	CCT ACT TCC AGT CCA GGT GGT A
Tbx2-F	5’-3’	TCA TCG CTG TCA CTG CCT ACC A
Tbx2-R	5’-3’	CGG CTT ACA GTG CTC CTC ATA C

Table S3. Antibodies used in Western blotting

Antigen	Host	Dilution	Company	Cat. No
PSD95	rabbit	1:2000	Abcam	ab18258
α-Actinin	mouse	1:1000	Proteintech	66895-1-IG
β-Tubulin	mouse	1:5000	Proteintech	66240-1-Ig
ZO-1	rabbit	1:1000	Invitrogen	61-7300
SYP	rabbit	1:2000	Abcam	ab32127

Table S4. Antibodies and dyes used in immunofluorescence staining.

Antigen	Host	Dilution	Company	Cat. No
ZO-1	rabbit	1:1000	Invitrogen	61-7300
PDGFRβ	rabbit	1:1000	Cell Signaling Technology	4564S
CD31	rabbit	1:1000	Abcam	ab28364
CD13	rat	1:200	Abcam	ab33489
Iba1	Rabbit	1:1000	Wako	019-19741
GFAP	mouse	1:1000	Servicebio	GB12096
βIII-tubulin	rabbit	1：500	Beyotime	AF1216
CD9	rabbit	1：2000	Abmart	T55337M
CD63	rabbit	1：2000	Abmart	T55253S
TSG101	rabbit	1：2000	Abmart	T55985M
Fibrinogen	rabbit	1：500	Solarbio	K114703P
DAPI	/	1:1000	Cell Signaling Technology	4083S
PKH26	/	/	Sigma-Aldrich	MINI26
EVlink 505	/	/	TINGOscience	EL012100200
anti-mouse IgG HRP-linked Ab	horse	1:1000	Cell Signaling Technology	7076S
anti-rabbit IgG HRP-linked Ab	horse	1:1000	Cell Signaling Technology	7074S

Table S5. Reagents used for flow cytometry:

Reagent	Catalog No.	Clone	Brand
Zombie NIR™ Fixable Viability Kit	423105	–	BioLegend
TruStain FcX™ (anti-mouse CD16/32)	101319	93	BioLegend
APC anti-mouse F4/80	123116	BM8	BioLegend
FITC anti-mouse CD45	157214	S18009F	BioLegend
APC/Cyanine7 anti-mouse CD3	100221	17A2	BioLegend
BV786 Rat anti-mouse CD4	563331	GK1.5	BioLegend
Brilliant Violet 605™ anti-mouse CD8a	100744	53-6.7	BioLegend
PE anti-mouse CD49b (pan-NK cells)	108907	–	BioLegend
Alexa Fluor® 700 anti-mouse/human CD11b	101222	M1/70	BioLegend
BV421 Rat anti-mouse Ly-6C	562727	AL-21	BD Horizon™
PE/Cyanine5 anti-mouse Ly-6G	127671	–	BioLegend
Brilliant Stain Buffer	563794	–	BD Horizon™

Table S6. Fluorescent dyes for in vivo two-photon imaging

Reagent	MW (kDa)	Company	Cat. No.	Dilution
Fluorescein isothiocyanate–dextran	4	Sigma-Aldrich	46944	10mg/ml in saline
Dextran, Texas Red	70	Invitrogen	D1830	10mg/ml in saline

Table S7. Other major reagents used in cell and animal experiments

Reagent	Company	Cat. No
Human Pericyte Medium (PM)	ScienCell Research Laboratories	1201
pericyte growth additive for PM	ScienCell Research Laboratories	1252
penicillin/streptomycin solution for PM	ScienCell Research Laboratories	0503
fetal bovine serum for PM	ScienCell Research Laboratories	0010
Dulbecco's modified Eagle's medium (DMEM)	Gibco	C11965500BT
phosphate-buffered saline solution (PBS)	Servicebio	G4202
Cyclosporine A (CSA)	Solarbio	SC5120
BCA assay	Beyotime	P0012S
0.3% Triton X-100	Sigma-Aldrich	X100
BSA	Sigma-Aldrich	A1933
Aβ1-42 lyophilized powder	GenScript	RP10017CN
Hexafluoroisopropanol (HFIP)	Macklin	H811027
dimethyl sulfoxide (DMSO)	Sigma-Aldrich	D2650
miRNA mimics /inhibitors	RiboBio	miR10002177-1
MicrON hsa-miR-486-5p agomir and negative control	RiboBio	miR40002177-4-5
1M Tris-HCl, pH 8.8	Beyotime	ST788
1M Tris-HCl, pH 6.8	Beyotime	ST768
RIPA lysis buffer	Beyotime	P0013B
miRNA 1st Strand cDNA Synthesis Kit (by tailing A)	Vazyme	MR201
Taq Pro Universal SYBR qPCR Master Mix	Vazyme	Q712
PrimeScript™ RT Master Mix (Perfect Real Time)	Shiga	RR036A
SYBR qPCR SuperMix Plus kit	Novoprotein	E096-01B
Nissol stain assay	Solarbio	DK0022100

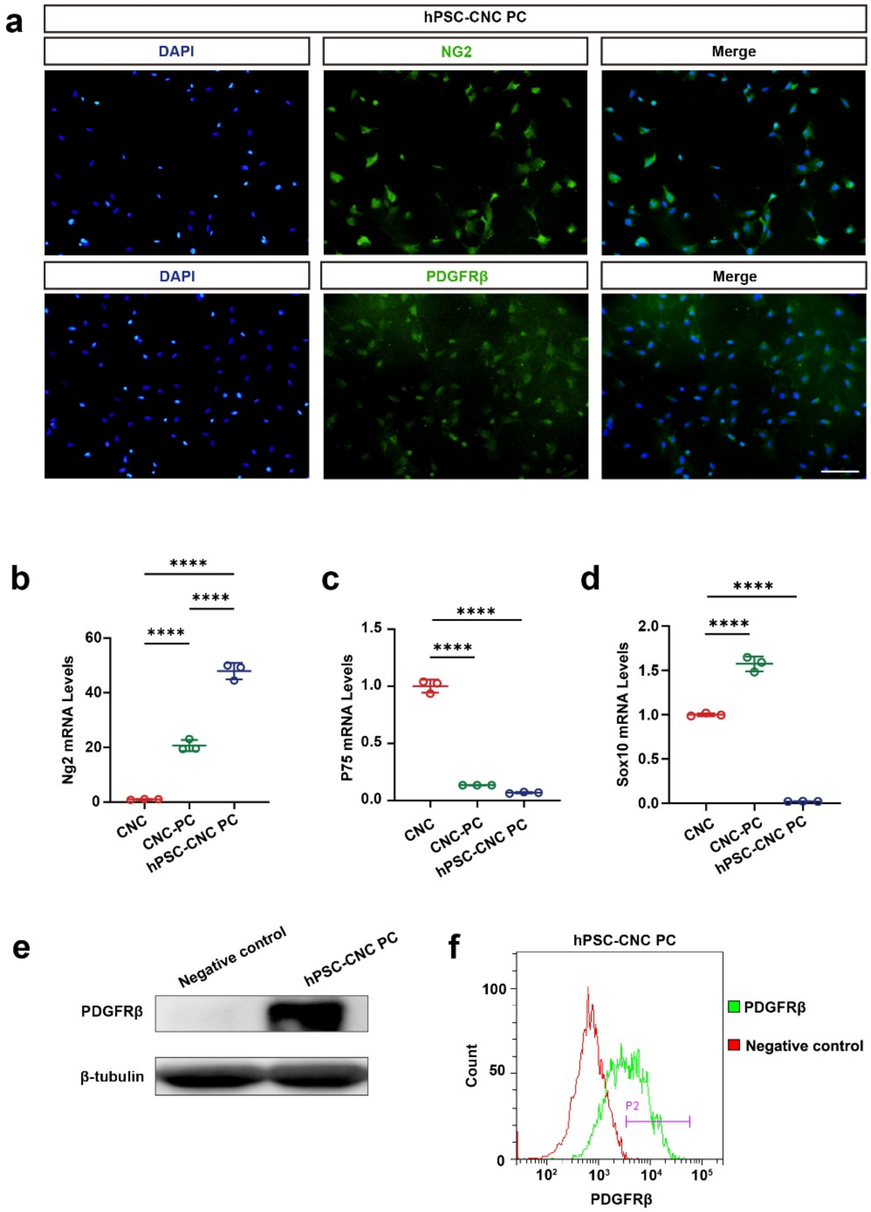

Figure S1. Characteristics of human pluripotent stem cell-derived pericyte-like cells (hPSC-PCs).

(a) Immunofluorescence staining indicated that the induced cells expressed the pericyte markers NG2 and PDGFRβ. (b–d) RT-PCR analysis demonstrated high *NG2* expression and low expression of the neural crest markers *P75* and *SOX10*. (e–f) Western blotting and flow cytometry further confirmed the high expression of the pericyte marker PDGFRβ in these cells. PDGFRβ-negative BV2 cells were used as a negative control.

(b, c, d) Data are presented as the mean ± S.E. The experiment had no less than three independent biological replicates (One-way ANOVA). ****, P<0.0001.

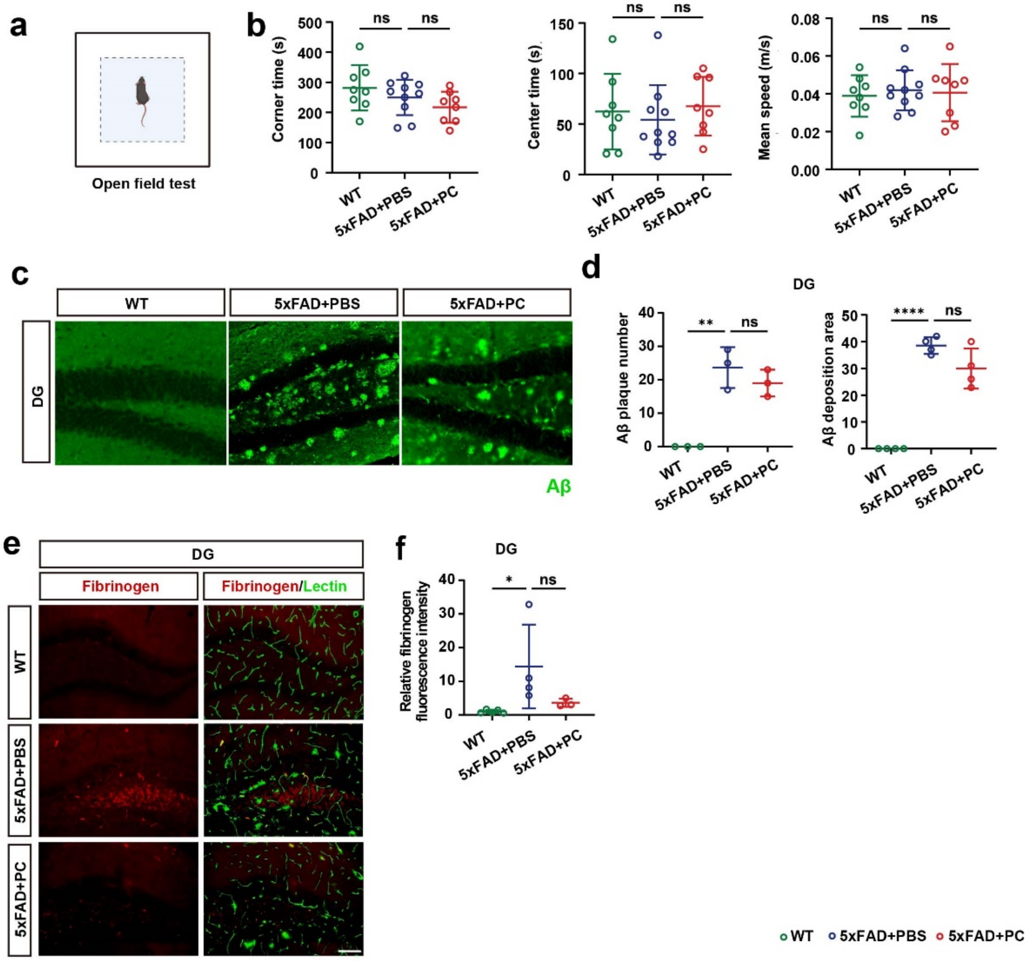

Figure S2. The hPSC-CNC PC treatment had no impact on mouse locomotion, and no differences of Aβ deposition and BBB leakage were found in DG area after cell injection.

(a) A diagram of open field test. (b) The hPSC-CNC PC treatment had no impact on the locomotion of the mice according to open field test. (c) Representative fluorescence photomicrographs of Aβ in the hippocampal DG area of WT, 5XFAD, and 5XFAD mice with hPSC-CNC PCs transplantation. (c-d) Fluorescence intensity and plaque number of Aβ in the hippocampal CA1 region were quantified in each visible field. No differences were found among three groups in the fluorescence area and plaque number of Aβ in DG. (e) Representative fluorescence photomicrographs of fibrinogen in the hippocampal DG area of WT, 5XFAD, and 5XFAD mice with hPSC-CNC PCs transplantation. (f) Immunofluorescence staining of fibrinogen revealed no significant differences of BBB leakage among three groups in the hippocampal DG region.

(b, d, f) Data are presented as the mean ± S.E. The experiment had no less than three independent biological replicates (One-way ANOVA). ns, P≥0.05; *, P<0.05; **, P<0.01; ****, P<0.0001.

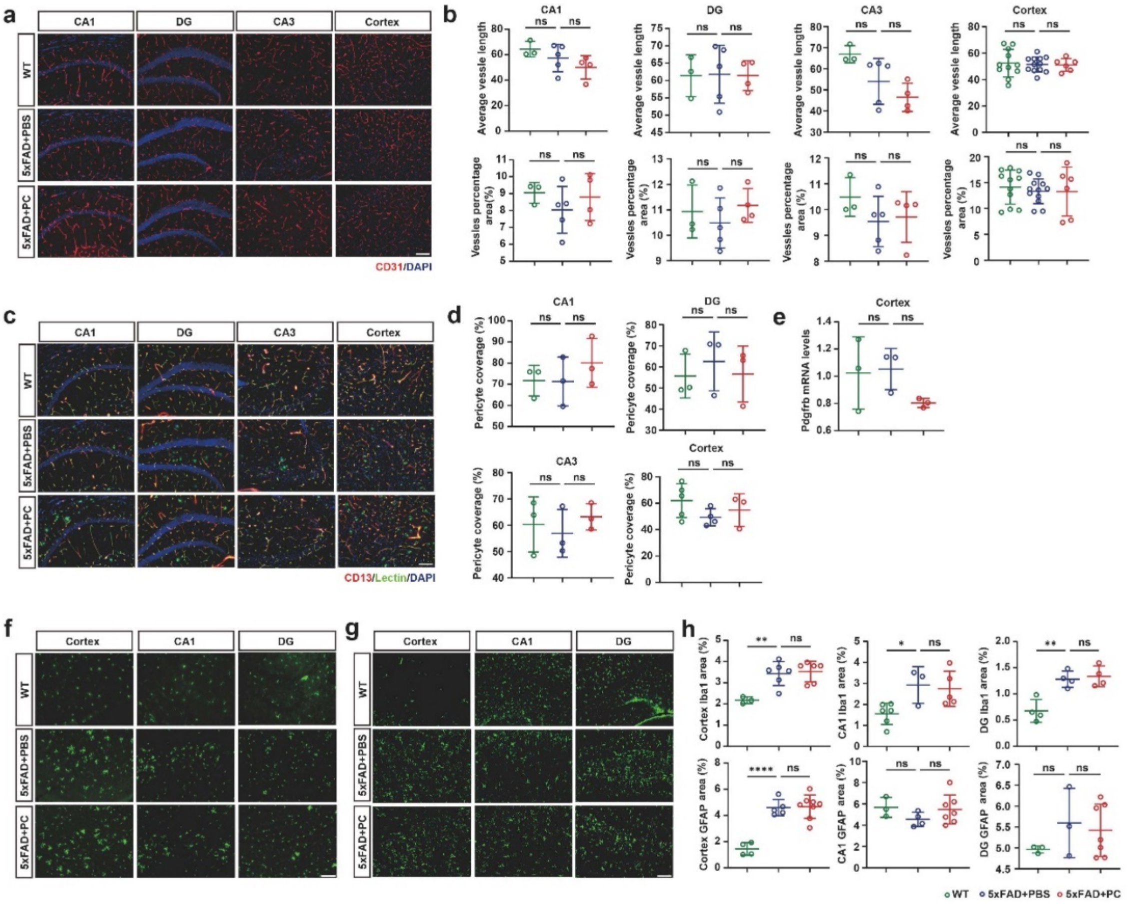

Figure S3. Other types of cells in the central nervous system did not change significantly after hPSC-CNC PC transplantation.

(a–b) Blood vessel length remained unchanged after hPSC-PCs injection. (c–e) Immunofluorescence and RT-PCR results showed no significant changes in the coverage area of pericytes or in the expression of the marker *Pdgfrb* RNA. (e) RT-PCR also indicated that while the RNA expression of inflammatory factors in the nervous system exhibited a downward trend post-cell injection, it did not reach statistical significance. (f-h) Immunofluorescence showed no significant changes in the fluorescence area of IBA1+ microglia or GFAP+ astrocyte after hPSC-PCs injection.

(b, d, e, h) Data are presented as the mean ± S.E. The experiment had no less than three independent biological replicates (One-way ANOVA). ns, P≥0.05; *, P<0.05; **, P<0.01; ****, P<0.0001.

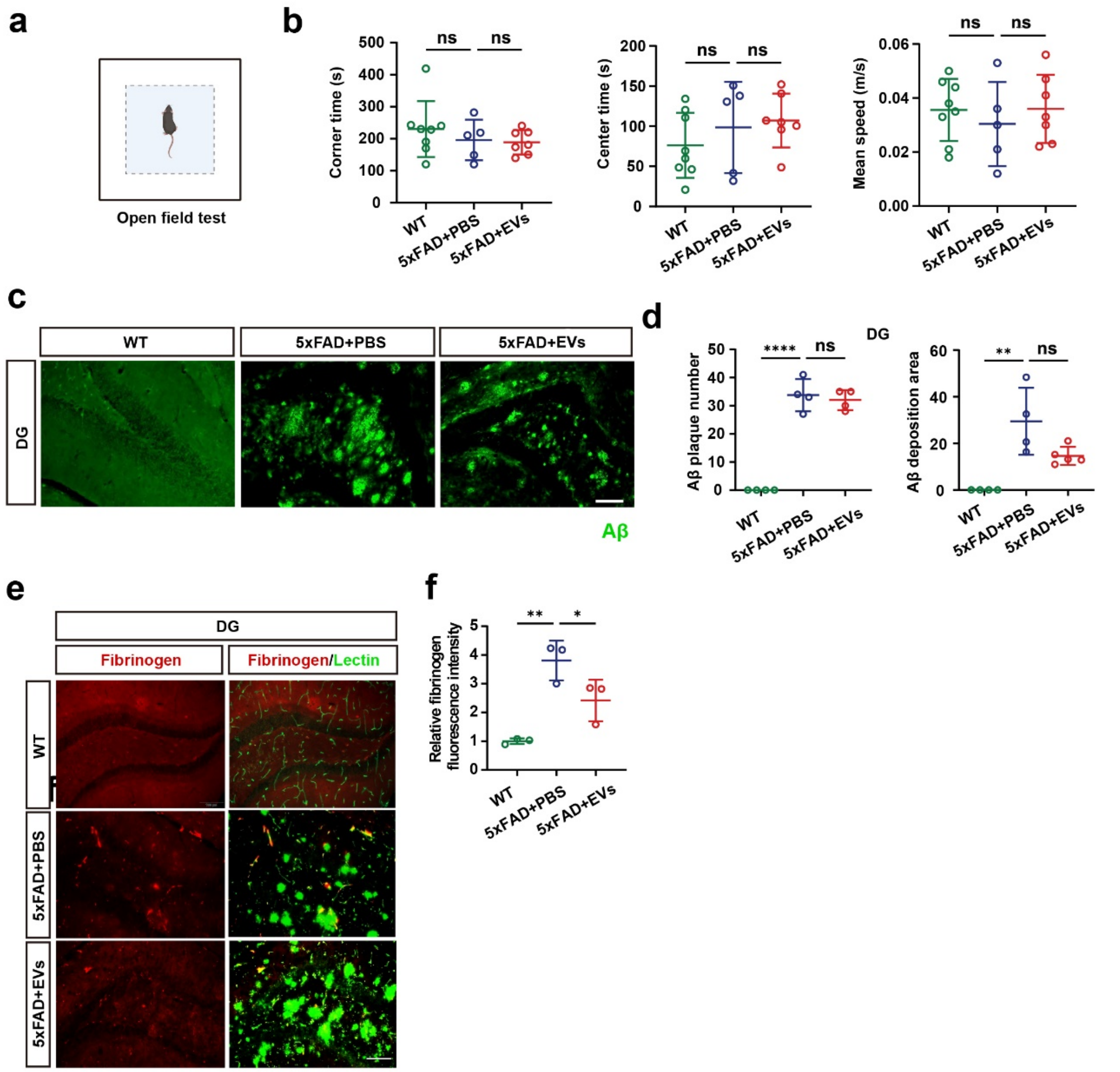

Figure S4. EV treatment had no impact on mouse locomotion or Aβ deposition in DG area, but decreased the BBB leakage in DG area.

(a) A diagram of open field test. (b) The EV treatment had no impact on the locomotion of the mice according to open field test. (c) Representative fluorescence photomicrographs of Aβ in the hippocampal DG area of WT, 5XFAD, and 5XFAD mice with EV transplantation. (d) No differences were found among three groups in the fluorescence intensity and plaque number of Aβ in DG. (e) Representative fluorescence photomicrographs of fibrinogen in the hippocampal DG area of WT, 5XFAD, and 5XFAD mice with EV transplantation. (f) Immunofluorescence staining of fibrinogen revealed significant decrease of BBB leakage in 5xFAD mice after EV injection in the hippocampal DG region.

(b, d, f) Data are presented as the mean ± S.E. The experiment had no less than three independent biological replicates (One-way ANOVA). ns, $P \geq 0.05$; *, $P<0.05$; **, $P<0.01$; ****, $P<0.0001$.

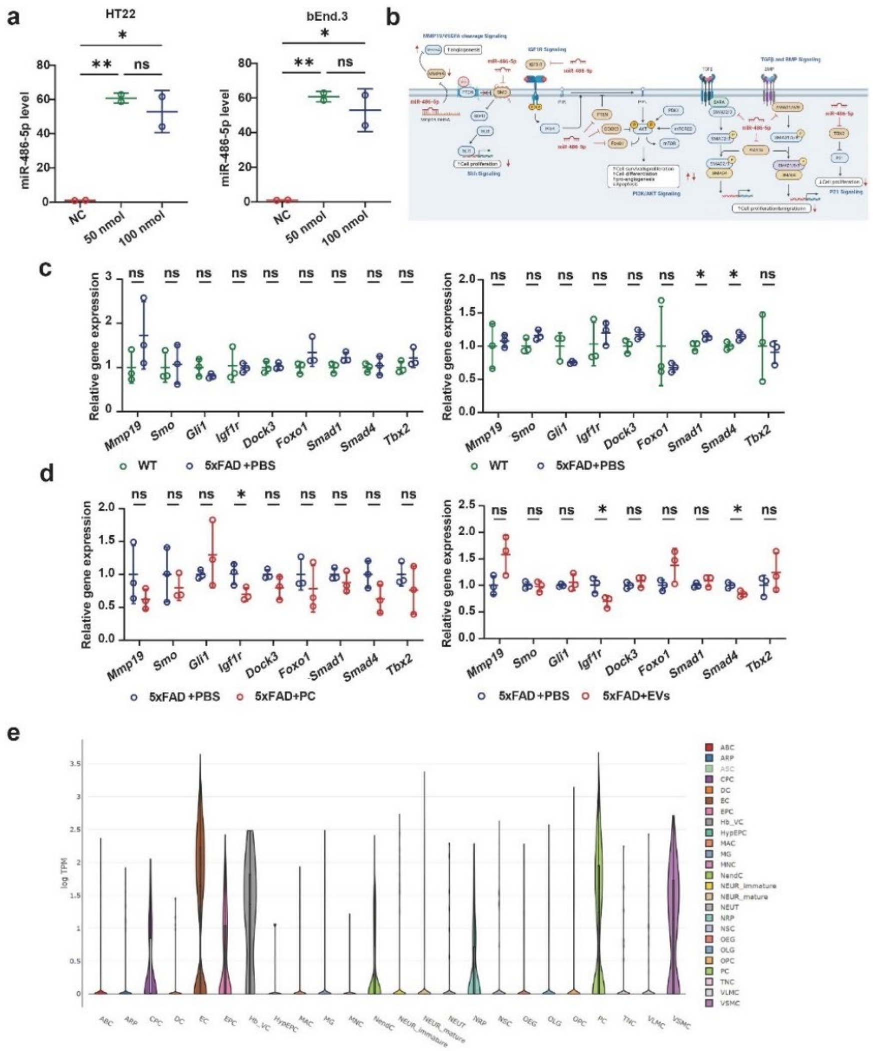

Figure S5. Potential downstream signaling pathways of miR-486-5p.

(a) An amount of 50nmol or 100nmol of miR-486-5P mimics could significantly increase the level of miR-486-5P in HT22 or bEnd.3 cells. (b) We conducted a preliminary exploration of potential downstream signaling pathways. Through sequencing and literature review, we identified miR-486-5p-related signaling pathways. (c–d) Further RT-PCR analysis of various molecules within these pathways revealed significant changes in type 1 insulin-like growth factor receptor (*IGF1R*), indicating that this molecule may be a critical downstream target. (e) *Igf1r* was found to be highly expressed in neurons and endothelial cells in mouse brain according to the published database (https://singlecell.broadinstitute.org/single_cell/study/SCP263/aging-mouse-brain) .

(a, c, d) Data are presented as the mean ± S.E. The experiment had no less than three independent biological replicates (One-way ANOVA). ns, P≥0.05; *, P<0.05; **, P<0.01.

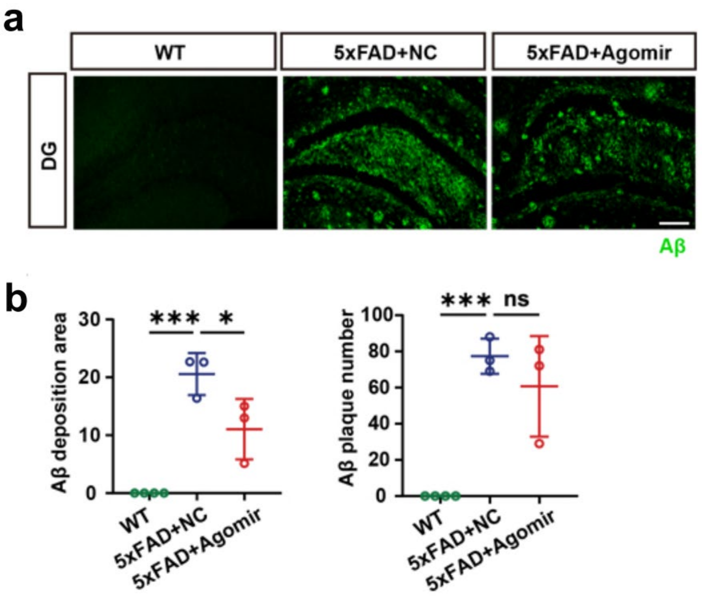

Figure S6. The miR-486-5P treatment decreased the Aβ deposition in DG area.

(a) Representative fluorescence photomicrographs of Aβ in the hippocampal DG area of WT, 5XFAD, and 5XFAD mice with EV transplantation. (b) Immunofluorescence staining revealed that Aβ deposition in the hippocampal DG region of 5xFAD mice decreased post agomir injection.

(b) Data are presented as the mean ± S.E. The experiment had no less than three independent biological replicates (One-way ANOVA). ns, P≥0.05; *, P<0.05; **, P<0.01.

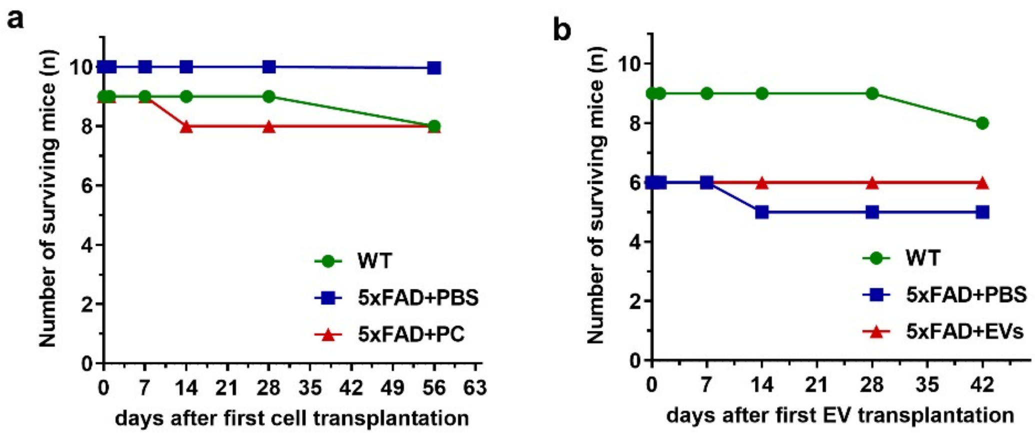

Figure S7. The number of surviving mice at different time points after cell or EV injection.

(a) The number of surviving mice at different time points after cell injection. (b) The number of surviving mice at different time points after EV injection.

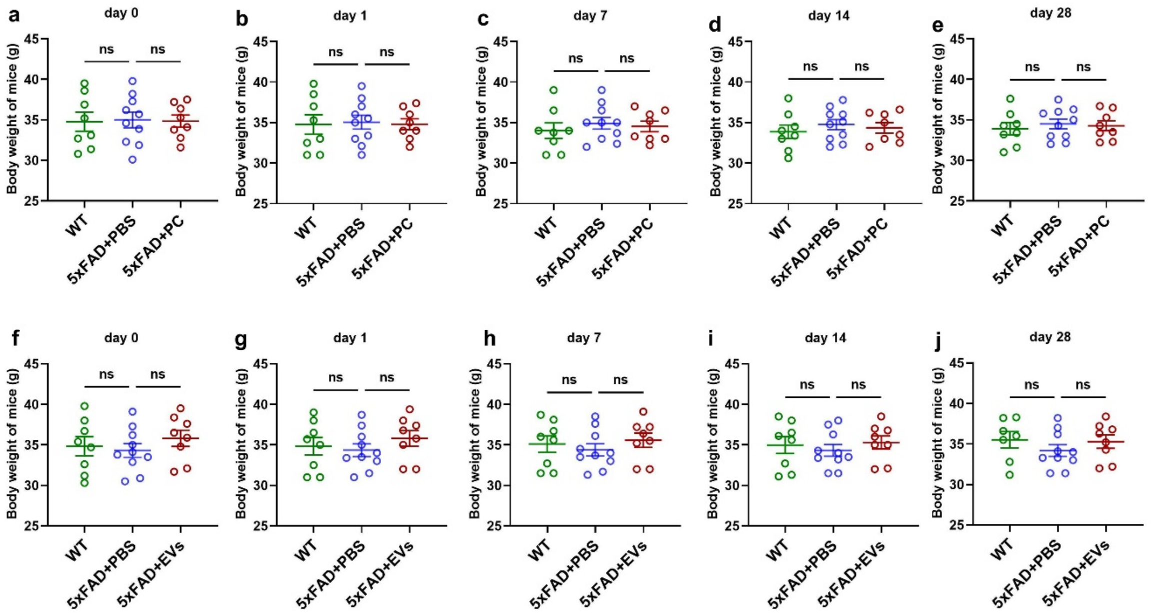

Figure.S8. Body weight of mice in each group at different time points.

Body weight of mice in each group on the day of injection (d0), as well as 1-, 7-, 14-, and 28-days post-injection. No significant decrease in body weight was observed at any of the examined time points. Data are presented as the mean ± S.E. The experiment had no less than three independent biological replicates (One-way ANOVA). ns, $P \geq 0.05$.

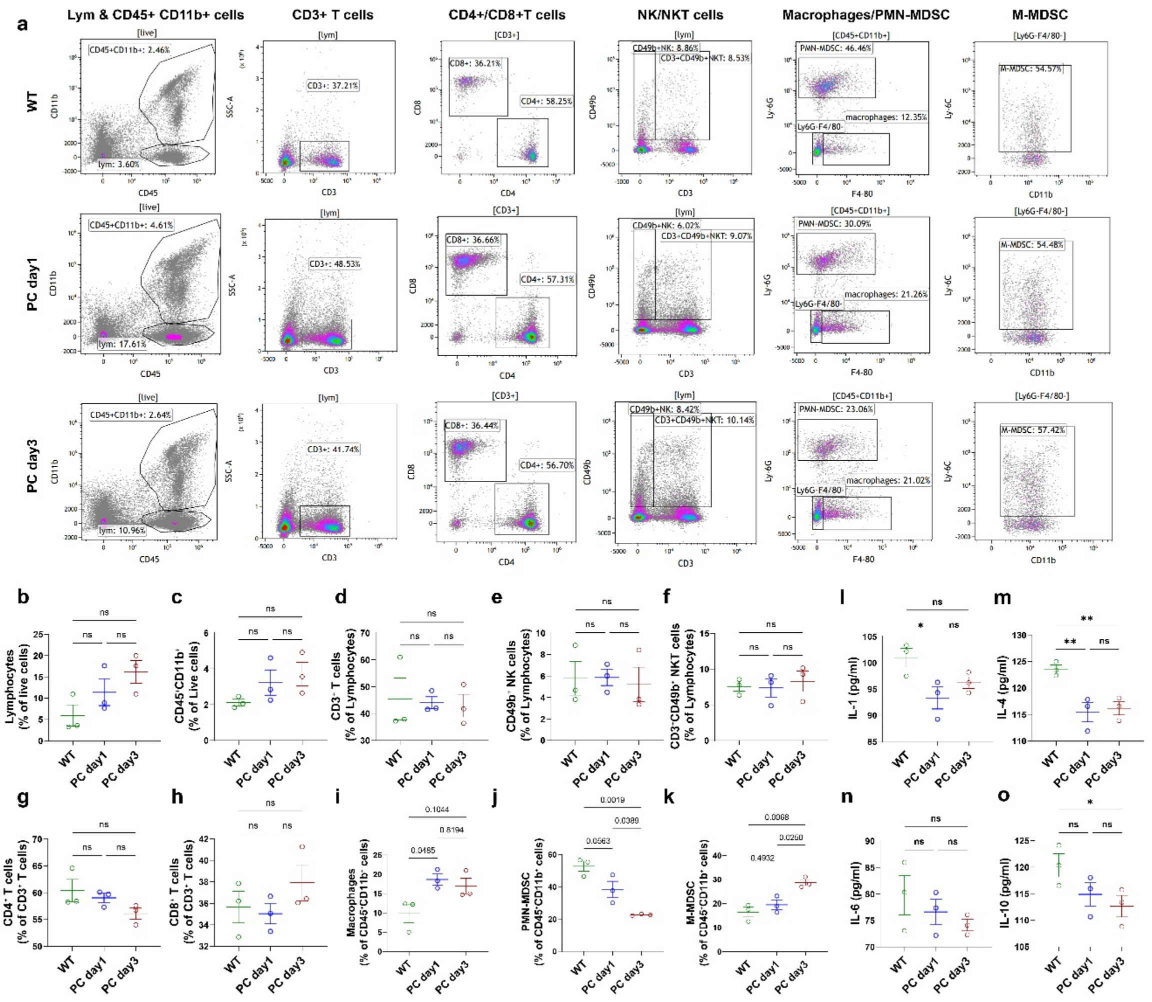

Figure S9. Landscape of peripheral blood immune cell populations and inflammatory cytokines 1 and 3 days after intravenous administration of hPSC-CNC PCs (PC).

(a) Flow cytometric analysis of peripheral blood immune cell dynamics in mice following intravenous injection of PC. Gating strategy and representative dot plots. (b–k) Quantification of immune cell subsets in wild-type mice at 1 and 3 days post-injection: lymphocytes (% of live cells), $CD45^+CD11b^+$ cells (% of live cells), $CD3^+$ T cells (% of lymphocytes), $CD49b^+$ NK cells (% of lymphocytes), $CD3^+CD49b^+$ NKT cells (% of lymphocytes), $CD4^+$ T cells (% of $CD3^+$ T cells), $CD8^+$ T cells (% of $CD3^+$ T cells), macrophages (% of $CD45^+CD11b^+$ cells), PMN-MDSCs (% of $CD45^+CD11b^+$ cells), and M-MDSCs (% of $CD45^+CD11b^+$ cells). (l–o) Serum levels of pro- and anti-inflammatory cytokines IL-1, IL-4, IL-6, and IL-10 measured in the same mice. Data are presented as mean ± SEM (n = 3 per group); statistical significance was determined by one-way ANOVA with Tukey's post hoc test (ns, $P \geq 0.05$; *$P < 0.05$; **$P < 0.01$).

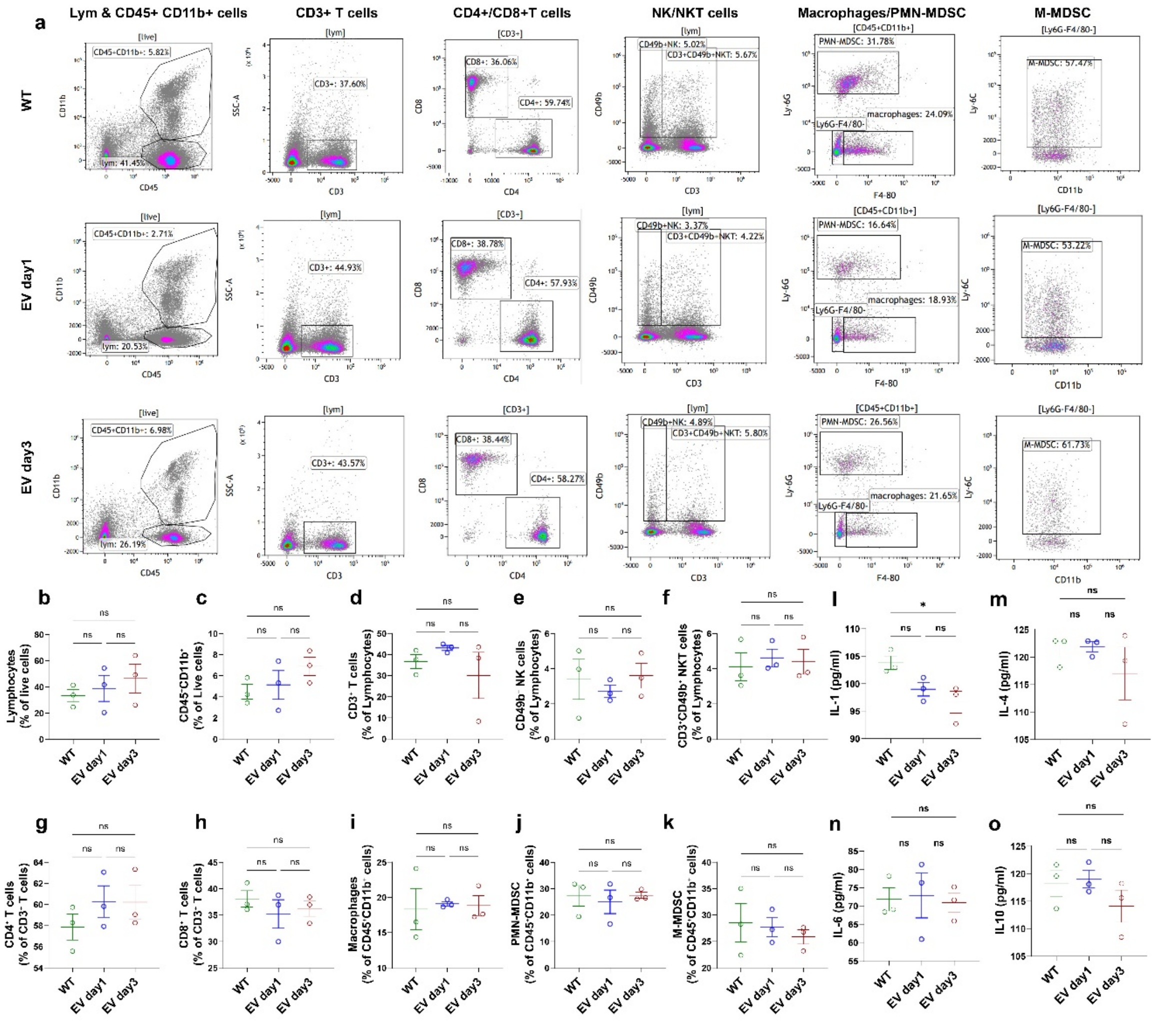

Figure S10. Landscape of peripheral blood immune cell populations and inflammatory cytokines 1 and 3 days after intravenous administration of EVs derived from hPSC-CNC PCs.

(a) Flow cytometric analysis of peripheral blood immune cell dynamics in mice following intravenous injection of EVs. Gating strategy and representative dot plots. (b–k) Quantification of immune cell subsets in wild-type mice at 1 and 3 days post-injection: lymphocytes (% of live cells), $CD45^{+}CD11b^{+}$ cells (% of live cells), $CD3^{+}$ T cells (% of lymphocytes), $CD49b^{+}$ NK cells (% of lymphocytes), $CD3^{+}CD49b^{+}$ NKT cells (% of lymphocytes), $CD4^{+}$ T cells (% of $CD3^{+}$ T cells), $CD8^{+}$ T cells (% of $CD3^{+}$ T cells), macrophages (% of $CD45^{+}CD11b^{+}$ cells), PMN-MDSCs (% of $CD45^{+}CD11b^{+}$ cells), and M-MDSCs (% of $CD45^{+}CD11b^{+}$ cells). (l–o) Serum levels of pro- and anti-inflammatory cytokines IL-1, IL-4, IL-6, and IL-10 measured in the same mice. Data are presented as mean ± SEM (n = 3 per group); statistical significance was determined by one-way ANOVA with Tukey's post hoc test (ns, $P \geq 0.05$; $*P < 0.05$; $**P < 0.01$).
