# Supplementary material for: Intravenously injected hPSC-derived pericytes for Alzheimer disease: Neuroprotection and vascular repair via extracellular vesicles
Source: Mol Ther. 2025 Aug 19;33(11):5681–703. doi: 10.1016/j.ymthe.2025.08.024 (PMC12628151; doi:10.1016/j.ymthe.2025.08.024)
Supplement: Document S2. Article plus supplemental information [file mmc2.pdf]

Molecular Therapy
Original Article

Intravenously injected hPSC-derived pericytes for Alzheimer disease: Neuroprotection and vascular repair via extracellular vesicles

Ying Liu,[1,2,5] Zhiyuan Ning,[1,3,5] Qingyuan Dai,[1,3,5] Xinkai Zhang,[1,3] Yibin Xiao,[1,3] Zhan Zhang,[1,3] Daji Guo,[1,3] Junhua Chen,[4] Yi Li,[1,3] Weiqiang Li,[4] Songhua Xiao,[1,3] and Yamei Tang[1,3]

[1]Department of Neurology, Sun Yat-sen Memorial Hospital, Sun Yat-sen University, No. 107, Yan Jiang Xi Road, Yuexiu District, Guangzhou, Guangdong 510120, China; [2]Huzhou Central Hospital, Fifth School of Clinical Medicine of Zhejiang Chinese Medical University, No. 1558, Sanhuan North Road, Huzhou, Zhejiang 313000, China; [3]Department of Brain Science, School of Medicine, Sun Yat-Sen University, No. 66, Gongchang Road, Guangming District, Shenzhen 518107, China; [4]Center for Stem Cell Biology and Tissue Engineering, Key Laboratory for Stem Cells and Tissue Engineering, Ministry of Education, Sun Yat-Sen University, No. 74, 2nd Yat-Sen Road, Yuexiu District, Guangzhou, Guangdong 510080, China

Intravenously injected human pluripotent stem cell (hPSC)-derived pericytes (PCs) and their extracellular vesicles (EVs) represent promising therapeutic strategies for neurological diseases. Our study aimed to investigate the effects and mechanisms of intravenous transplantation for treating Alzheimer disease (AD), with a focus on elucidating the critical role of EV-related mechanisms. We generated PCs (hPSC-CNC PCs) from hPSC-derived cranial neural crest (CNC) and employed 12-month-old 5xFAD mice as an advanced stage AD model. We investigated memory function, intracerebral β-amyloid (Aβ) deposition, blood-brain barrier (BBB) permeability, neuronal morphology, and associated protein expressions in mice to determine the therapeutic effects of intravenous administration of hPSC-CNC PCs or EVs. miRNA sequencing was conducted to identify potential downstream pathways. We found that intravenous administration of hPSC-CNC PCs improved memory function of aged AD mice, concurrently reducing pathological deposits and BBB leakage and enhancing neurofunctional outcomes via EVs. Furthermore, miRNA-486-5p in EVs might promote neurovascular repair through various mechanisms. Our results demonstrated that EVs from hPSC-CNC PCs exert protective effects against AD.

INTRODUCTION

Alzheimer disease (AD) is the most common form of dementia, characterized by a gradual decline in cognitive abilities that significantly impairs an individual's functionality as the disease advances.[1] Current estimates are alarming, with 55 million people currently living with AD, a number projected to rise to 139 million by 2050.[2] This escalating prevalence underscores AD as a critical global public health concern, imposing a profound economic burden on society and families.[3] Despite recent advancements in AD research, the primary pathogenic hypotheses remain the "amyloid cascade hypothesis" and the "cholinergic dysfunction hypothesis."[4] While anti-amyloid antibody drugs have demonstrated β-amyloid (Aβ) plaque-clearing effects in preclinical studies, most have been discontinued in phase 3 clinical trials due to their inability to improve cognitive function. Although two drugs, aducanumab and lecanemab, were approved by the US Food and Drug Administration (FDA), they are indicated only for patients with mild cognitive impairment (MCI) or early-stage AD, and may cause side effects such as brain edema.[5] In addition, traditional cholinergic system-based drugs, such as donepezil, provide only short-term symptom relief.[6] To date, no definitive cure for AD has been found. Current treatments can only prevent or delay symptoms, but they cannot reverse disease progression. Therefore, there is an urgent need for the continued development of novel, effective therapeutic strategies for AD.

Embedded in the basement membrane of blood microvessels,[7] pericytes (PCs) occupy a unique position within the neurovascular unit, interacting with endothelial cells, astrocytes, and neurons of the central nervous system (CNS). Degeneration or loss of PCs has been implicated as a contributory factor in the progression of neurodegenerative diseases, including AD, Parkinson disease, and Huntington disease.[8,9] During the progression of AD, substantial alterations in

Received 8 January 2025; accepted 14 August 2025;
https://doi.org/10.1016/j.ymthe.2025.08.024.

[5]These authors contributed equally

Correspondence: Weiqiang Li, M.D., Ph.D., Center for Stem Cell Biology and Tissue Engineering, Key Laboratory for Stem Cells and Tissue Engineering, Ministry of Education, Sun Yat-Sen University, No. 74, 2nd Yat-Sen Road, Yuexiu District, Guangzhou, Guangdong 510080, China.
E-mail: liweiq6@mail.sysu.edu.cn
Correspondence: Songhua Xiao, M.D., Ph.D., Department of Neurology, Sun Yat-sen Memorial Hospital, Sun Yat-sen University, No. 107, Yan Jiang Xi Road, Yuexiu District, Guangzhou, Guangdong 510120, China.
E-mail: xiaosh@mail.sysu.edu.cn
Correspondence: Yamei Tang, M.D., Ph.D., Brain Research Center, Department of Neurology, Sun Yat-sen Memorial Hospital, Sun Yat-sen University, No. 107, Yan Jiang Xi Road, Yuexiu District, Guangzhou, Guangdong 510120, China.
E-mail: tangym@mail.sysu.edu.cn

© 2025 The Authors.
Published by Elsevier Inc. on behalf of The American Society of Gene and Cell Therapy.
This is an open access article under the CC BY license (http://creativecommons.org/licenses/by/4.0/).

both the number and function of PCs contribute directly to the disruption of the blood-brain barrier (BBB), thereby exacerbating neuroinflammation and accelerating pathological progression.[10] Furthermore, PC dysfunction impairs critical processes such as neuronal physiology and synaptic transmission, both of which are strongly associated with the cognitive decline observed in AD patients.[11]

Consequently, PC transplantation has emerged as an innovative therapeutic approach for AD and other CNS disorders. With their robust advantages of tissue regeneration, self-renewal, and differentiation potential, pluripotent stem cells (PSCs), such as induced PSCs (iPSCs) and mesenchymal stem cells (MSCs), have emerged as promising sources of stem cells.[12] These cells exhibit multipotency and can be induced to differentiate into various brain cell types, including PCs,[13] neurons,[14] and microglia.[15] Tachibana et al. demonstrated that stereotactic injection of MSC-derived PCs into the brains of APP/PS1 mice significantly improved cerebral microcirculation and reduced Aβ plaque accumulation.[16] Furthermore, while research on PC transplantation in AD is still limited, its potential to enhance cerebrovascular function, reduce BBB leakage, and mitigate neuronal damage has been validated in other disease models. For example, Zhang et al. injected PCs derived from rat adipose tissue into the brains of rats, which improved BBB leakage, promoted endothelial cell-cell adhesion and tight junctions, and reduced neuronal damage, thus treating neurological dysfunction in mice after intracerebral hemorrhage.[17]

Although research on human stem cell-derived brain PCs is still in its early stages, significant progress has been made. A study by Sun et al. successfully generated PCs from cranial neural crest (CNC) cells derived from human PSCs (hPSCs), which expressed typical PC markers and exhibited distinct PC characteristics. Moreover, when these hPSC-CNC-derived PCs were intravenously administered into a rat model of middle cerebral artery occlusion (MCAO), they significantly promoted neurological recovery by reconstructing the BBB integrity and preventing neuronal apoptosis.[13] These findings suggest that stem cell-derived PCs not only serve as an ideal model for studying human brain PC function and cell transplantation but also exhibit therapeutic potential in improving cerebrovascular function, enhancing BBB integrity, and reducing neuronal damage. However, its precise efficacy and underlying mechanisms in the treatment of AD require further investigation. Notably, PC transplantation may differ from mesenchymal cell transplantation in terms of target location and mechanism of action and the bioactive components secreted. While MSC-based transplantation therapies have shown therapeutic efficacy in AD mouse models, their ability to differentiate into neuronal cell types determines that the primary mechanisms of action are neuronal replacement, promoting neuronal regeneration, and mediating inflammation.[18] In contrast, as a key component of the neurovascular unit, PCs can specifically target endothelial cells, thus regulating the function of the neurovascular unit.[17]

However, the engraftment rate of transplanted cells is typically low and cell transplantation has risks such as cell dedifferentiation, immune rejection, and malignant tumor formation.[19–21] Evidence has illuminated that the beneficial effects of transplanted cells are predominantly attributed to the extracellular vesicles (EVs) they secrete.[22,23] Cells dynamically regulate the molecular composition and functional characteristics of the extracellular matrix and facilitate intercellular communication by transporting active components, such as proteins and functional microRNAs (miRNAs), via EVs.[24]

It is noteworthy that compared to direct cell transplantation, EVs offer significant safety advantages. They not only naturally cross the BBB[25] but also exhibit characteristics such as low immunogenicity and minimal cellular toxicity.[26] The low immunogenicity may be closely linked to the immunomodulatory functions of EVs. Previous studies have demonstrated that EVs regulate various inflammatory signaling pathways, including the nuclear factor κB pathway, which in turn modulates a range of inflammatory cytokines, such as interleukin-1 (IL-1), IL-6, IL-10, IL-4, tumor necrosis factor (TNF), and interferons (IFNs).[27,28] This mechanism helps maintain immune homeostasis by suppressing excessive immune activation. Among the various components in EVs, miRNAs are particularly noteworthy due to their regulatory functions. miRNAs are endogenous, non-coding RNAs, about 22 nt long, that regulate gene expression by binding to the 3′ untranslated region of target mRNAs.[29] In the CNS, miRNAs play key roles in angiogenesis, BBB homeostasis, neurogenesis, synaptic plasticity, and neuroinflammation.[30] In AD, several miRNAs show altered expression, making them potential therapeutic targets. For example, Ouyang et al. showed that EV-delivered miR-124 inhibited β-secretase 1, reducing Aβ plaque deposition and improving cognitive function.[31] Another study demonstrated that miR-219 mimics reduced tau phosphorylation and improved memory dysfunction in tauopathy mouse models.[29] These findings highlight the potential of miRNAs in AD treatment and the clinical promise of using EVs or engineered carriers to deliver therapeutic miRNAs.

Research on PC-derived EVs remains in its infancy. The concept of PC-derived EVs was introduced in 2018.[32] Under physiological conditions, PCs integrate, coordinate, and process signals from their neighboring cells through EVs, generating diverse functional responses pivotal to CNS functions, including regulation of the BBB permeability, angiogenesis, clearance of toxic metabolites, and capillary hemodynamic responses.[33,34] In contrast, under pathological conditions, the expression profile of PC EVs undergoes substantial changes, contributing to the spread of pathological proteins and the propagation of neuronal damage. This, in turn, triggers a cascade of events that accelerates the progression of neurodegenerative diseases.[32,35] Previous studies have demonstrated that EVs derived from healthy PCs, when administered via intravenous or intranasal injection, can effectively treat disease models such as Parkinson disease.[36] These findings suggest that PC-derived EVs may offer a promising therapeutic approach for addressing vascular and neuronal injury in neurodegenerative diseases. However, the precise mechanisms underlying the actions of PC-derived EVs in vascular-neuronal

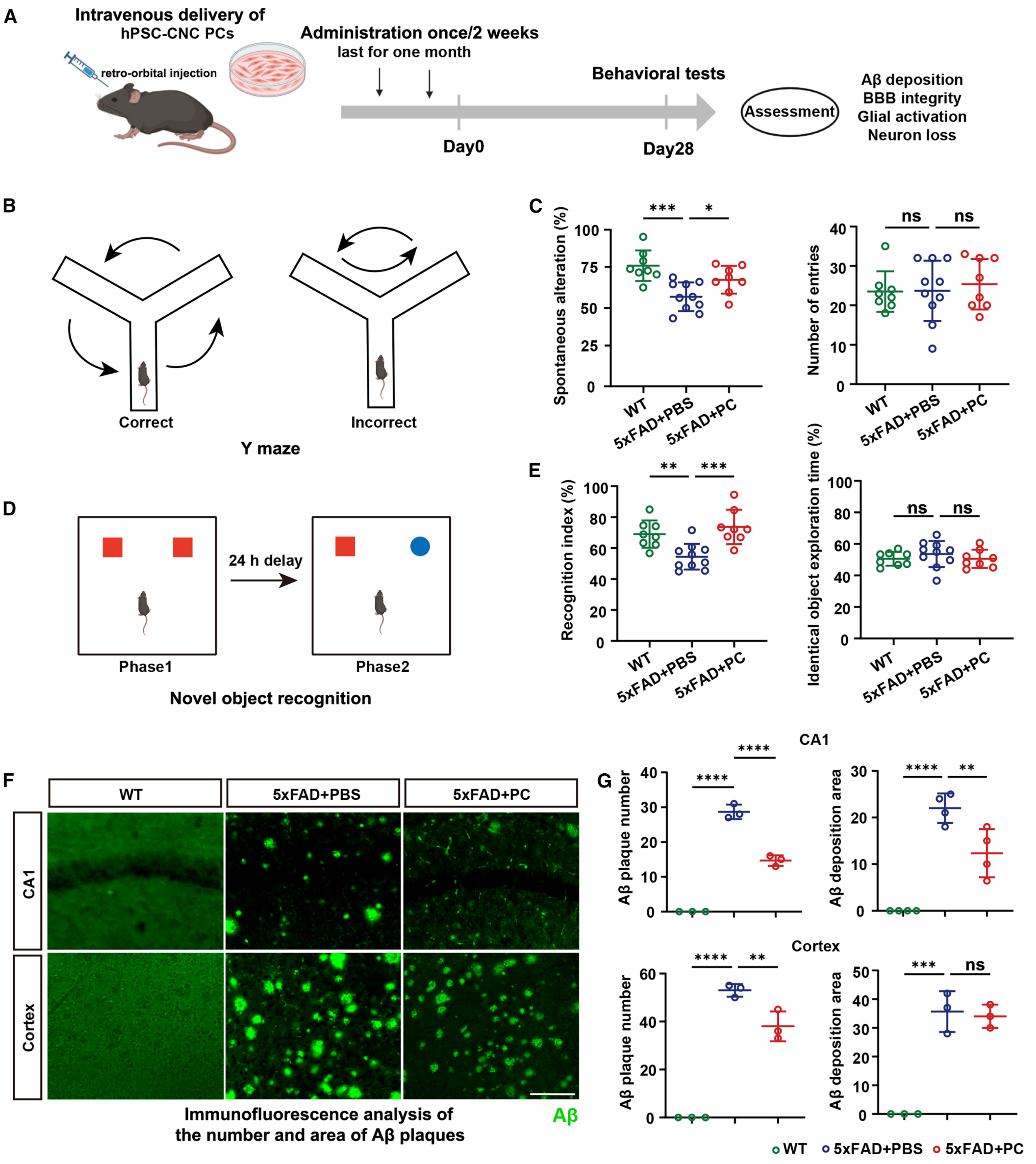

Figure 1. Intravascular transplantation of hPSC-CNC PCs improved cognitive function and reduced Aβ deposition in 5xFAD mice

(A) Experimental design (created with BioRender.com). (B) Diagram of the Y-maze. (C) Spontaneous alteration (left) and number of entries (right) measured in Y-maze test. The 12-month-old 5xFAD mice exhibited reduced spontaneous alteration, and intravenous hPSC-CNC PCs transplantation improved performance. Data are presented as the mean ± SE. $N_{WT} = 8$, $N_{5xFAD+PBS} = 10$, $N_{5xFAD+PC} = 8$ (one-way ANOVA). ns, $p \geq 0.05$; *$p < 0.05$; **$p < 0.01$; ***$p < 0.001$. (D) Experimental design for novel object recognition (NOR) test. (E) Recognition index in phase 2 (left) and identical object exploration time in phase 1 (right) in NOR test. The 12-month-old 5xFAD mice exhibited reduced

(legend continued on next page)

function remain poorly understood, and their therapeutic potential in AD requires further investigation.

In our study, we revealed that intravenous administration of hPSC-CNC PCs into 5xFAD mice enabled a significant recovery of cognitive function by reducing the Aβ deposits, restoring BBB properties, and preventing neuron death in a paracrine way. Moreover, intravenous transplantation of EVs derived from hPSC-CNC PCs demonstrated comparable therapeutic effects in AD mouse models. Supporting this, *in vitro* experiments revealed that EVs, distinct from other secreted protein components, could enhance neuronal and endothelial functions. Furthermore, we found that activating miRNA-486-5p, the key component of hPSC-PC-derived EVs, also restored the cognition impairment, reduced Aβ deposits and BBB leakage, and improved neural function, potentially through the insulin-like growth factor 1 receptor (IGF1R) pathway. Our data demonstrated that EVs from human PCs can regulate vascular stability and enhance neuron survival in AD.

RESULTS

Intravenous transplantation of hPSC-CNC PCs improved cognitive function and reduced Aβ deposition in 5xFAD mice

As previously described, we successfully derived hPSC-CNC PCs using a neural crest differentiation protocol.[13] Immunofluorescence staining indicated that hPSC-CNC PCs expressed the PC markers neuron-glia antigen 2 (NG2) and platelet-derived growth factor receptor β (PDGFRβ) (Figure S1A). Additionally, real-time quantitative reverse transcription polymerase chain reaction (RT-qPCR) analysis demonstrated high *NG2* expression and low expression of the neural crest markers *P75* and sex-determining region Y-box transcription factor 10 (*SOX10*) in hPSC-CNC PCs (Figures S1B–S1D). Western blotting and flow cytometry further confirmed the high expression of the PC marker PDGFRβ in hPSC-CNC PCs, with PDGFRβ^{-} BV2 cells used as a negative control (NC) (Figures S1E and S1F).

To evaluate the therapeutic potential of hPSC-CNC PCs transplantation for AD, we administered hPSC-CNC PCs intravenously to 12-month-old 5xFAD mice via retro-orbital injection, performed once every 2 weeks for 1 month. The control group was injected with the same dose of phosphate-buffered saline (PBS) at the same time. We then assessed whether hPSC-CNC PC transplantation improved the cognition impairment and pathological changes in 5xFAD mice (Figure 1A). The hPSC-CNC PC treatment had no impact on the locomotion of the mice according to the open field test (Figures S2A and S2B). In the Y Maze test to assess working memory (Figures 1B and 1C), the 12-month-old 5xFAD mice with PBS injection exhibited significant cognitive decline, as evidenced by reduced Y-maze spontaneous alteration rate (Figure 1C, $P_{WT\ vs.\ 5xFAD+PBS} = 0.0003$). However, after intravenous hPSC-CNC PCs transplantation, 5xFAD mice showed better Y-maze performance (Figure 1C, $P_{5xFAD+PBS\ vs.\ 5xFAD+PC} = 0.0417$), and the total number of arm crossings did not differ among the three groups (Figure 1C). Similar results were shown in the novel object recognition (NOR) (Figures 1D and 1E). The mice in the PBS group exhibited a reduced recognition index compared to the age-matched wild-type (WT) mice (Figure 1E, $P_{WT\ vs.\ 5xFAD+PBS} = 0.0088$). However, after intravenous hPSC-CNC PCs transplantation, 5xFAD mice showed better recognition index (Figure 1E, $P_{5xFAD+PBS\ vs.\ 5xFAD+PC} = 0.0007$), and no differences of identical object exploration time were found among the three groups (Figure 1E). Furthermore, immunofluorescence staining revealed that Aβ deposition in the hippocampus CA1 and the cortex of 5xFAD mice decreased to varying degrees post-transplantation, both in plaque number (Figures 1F and 1G, left, CA1: $P_{5xFAD+PBS\ vs.\ 5xFAD+PC} < 0.0001$; cortex: $P_{5xFAD+PBS\ vs.\ 5xFAD+PC} = 0.0080$) and deposition area (Figures 1F and 1G, right, CA1: $P_{5xFAD+PBS\ vs.\ 5xFAD+PC} = 0.0090$), while the Aβ deposition showed no differences in hippocampus dentate gyrus (DG) (Figures S2C and S2D) Collectively, these data revealed that hPSC-CNC PCs transplantation restored 5xFAD mice cognitive behaviors probably by reducing the Aβ deposition.

hPSC-CNC PCs transplantation reduced BBB injury and restored neurological function in 5xFAD mice

In AD, the development of BBB leakage allows vascular substances, such as fibrinogen, albumin, and immunoglobulins, to infiltrate the brain parenchyma, which further impair brain function.[37] In accordance with previous studies, immunofluorescence staining of fibrinogen revealed significant BBB leakage in 12-month-old 5xFAD mice (Figures 2A, 2B, S2E, and S2F), particularly in the cortex ($P_{WT\ vs.\ 5xFAD+PBS} = 0.0286$) and hippocampus (CA1: $P_{WT\ vs.\ 5xFAD+PBS} = 0.0001$; DG: $P_{WT\ vs.\ 5xFAD+PBS} = 0.8753$). Intravenous transplantation of hPSC-CNC PCs significantly reduced BBB leakage in both cortical and hippocampal regions of 5xFAD mice (cortex: $P_{5xFAD+PBS\ vs.\ 5xFAD+PC} = 0.0350$; CA1: $P_{5xFAD+PBS\ vs.\ 5xFAD+PC} = 0.0091$; DG: $P_{5xFAD+PBS\ vs.\ 5xFAD+PC} = 0.1788$). Using *in vivo* two-photon imaging, we observed obvious BBB disruption as indicated by leakage of 4 and 70 kDa dextran into the brain parenchyma, and hPSC-CNC PCs transplantation reduced BBB leakage in the cortex of 5xFAD mice (Figure 2C). Furthermore, the results of Nissl Staining demonstrated an increase in the number of neurons and morphological recovery in the cortex of mice following cell injection (Figure 2D; neuron number: $P_{5xFAD+PBS\ vs.\ 5xFAD+PC} = 0.0008$; soma size: $P_{5xFAD+PBS\ vs.\ 5xFAD+PC} = 0.0425$).

recognition index, and intravenous hPSC-CNC PCs transplantation improved NOR performance. Data are presented as the mean ± SE. $N_{WT} = 8$, $N_{5xFAD+PBS} = 10$, $N_{5xFAD+PC} = 8$ (one-way ANOVA). ns, $p \geq 0.05$; *$p < 0.05$; **$p < 0.01$; ***$p < 0.001$. (F and G) Fluorescence intensity and plaque number of Aβ in cortex and hippocampal CA1 region in each visible field. The Aβ deposition in the hippocampus CA1 (top) and the cortex (bottom) of 5xFAD mice decreased to varying degrees post-transplantation, both in plaque number and deposition area. (G) Data are presented as the mean ± SE. The experiment had no less than three independent biological replicates (one-way ANOVA). ns, $p \geq 0.05$; **$p < 0.01$; ***$p < 0.001$; ****$p < 0.0001$.

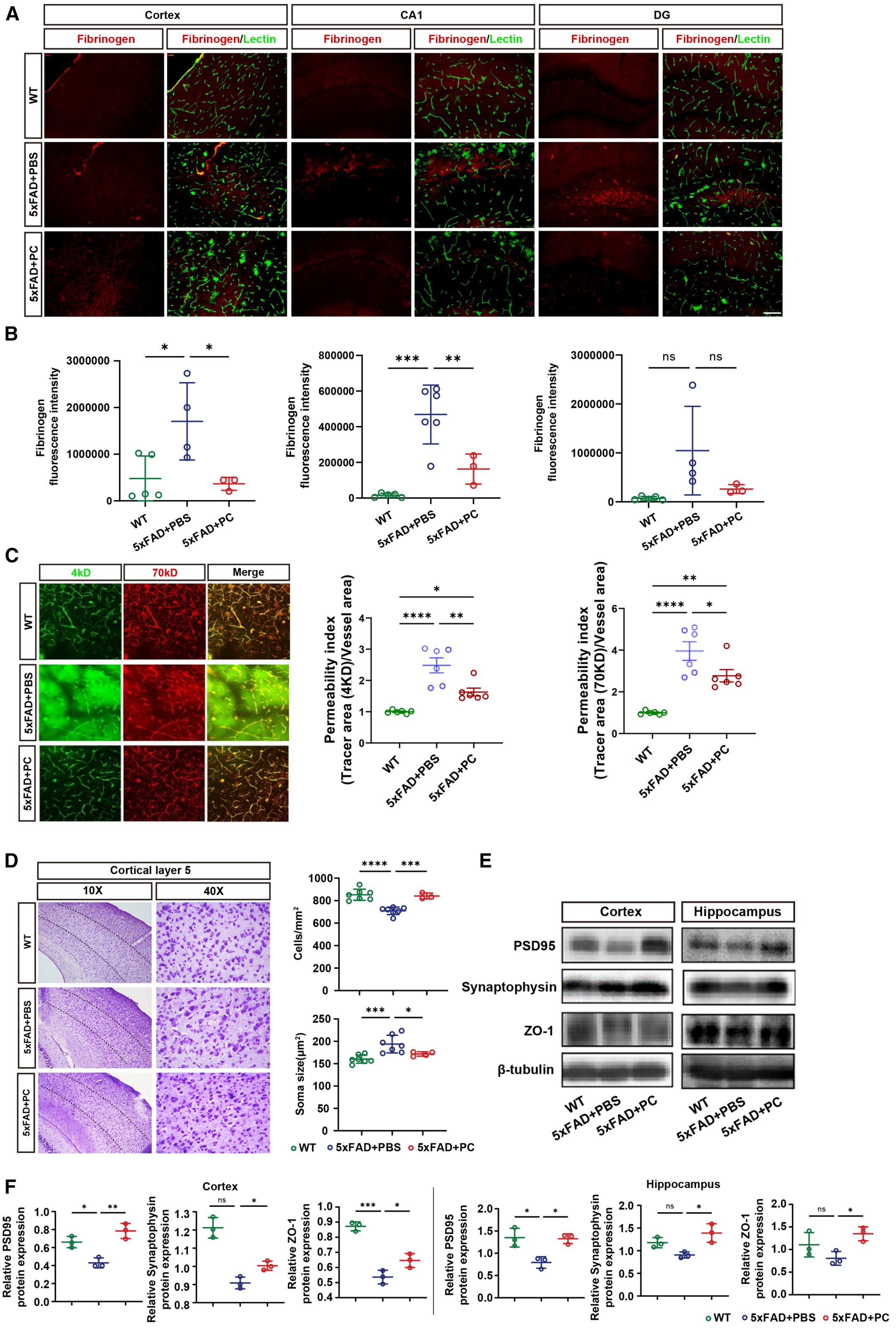

(legend on next page)

Moreover, we found upregulated expression of tight junction proteins marker zonula occludens-1 (ZO-1) in 5xFAD mice after hPSC-CNC PCs injection (Figures 2E and 2F; cortex: $P_{5xFAD+PBS\ vs.\ 5xFAD+PC} = 0.0499$; hippocampus: $P_{5xFAD+PBS\ vs.\ 5xFAD+PC} = 0.0363$). Additionally, cell transplantation increased the expression of synaptic proteins, including postsynaptic density protein 95 (PSD95) (Figures 2E and 2F; cortex: $P_{5xFAD+PBS\ vs.\ 5xFAD+PC} = 0.0022$; hippocampus: $P_{5xFAD+PBS\ vs.\ 5xFAD+PC} = 0.0134$) and synaptophysin (Figures 2E and 2F; cortex: $P_{5xFAD+PBS\ vs.\ 5xFAD+PC} = 0.0164$; hippocampus: $P_{5xFAD+PBS\ vs.\ 5xFAD+PC} = 0.0133$) in the hippocampus and cortex of 5xFAD mice.

There were, however, no significant changes in the vascular length and areas (Figures S3A and S3B), the coverage area of PCs (Figures S3C and S3D), and the expression of the marker *PDGFRβ* RNA (Figure S3E). Glia activation is also a common pathological feature of AD.[38] We found increased ionized calcium-binding adapter molecule 1 (IBA1), a marker of microglia, and glial fibrillary acidic protein (GFAP), a marker of astrocytes in 5xFAD mice, and similarly increased numbers of microglia and astrocytes were observed in cell transplantation group (Figures S3F–S3H). The administration of hPSC-CNC PCs was observed to alleviate BBB leakage and restore neuronal morphology, which might primarily be attributed to influencing the expression of endothelial and neuronal functional proteins, rather than affecting PC coverage or glial cell activation.

hPSC-CNC PCs did not accumulate in the mouse CNS after intravenous transplantation

To figure out how hPSC-CNC PCs work in 5xFAD mice, we used a PKH26 kit to label hPSC-CNC PCs, which allowed the cells to exhibit stable red fluorescence on their membrane. Following intravenous transplantation, we examined frozen sections of peripheral and brain tissues for PKH26 fluorescence signal intensity and area, and specific sequences for hPSC-CNC PCs were detected by RT-qPCR (Figure 3A). No PKH26 fluorescence signal was observed in the brain (Figure 3B, left), whereas significant cell aggregation was detected in the lung tissue within minutes after cell injection (Figure 3B, right). However, the cells were gradually cleared, nearly disappearing by day 3 (Figure 3C). To confirm cell presence, we performed human-specific *ALU* and glyceraldehyde-3-phosphate dehydrogenase (*GAPDH*) sequence (Figures 3D and 3E) detection in both peripheral and brain tissues, and we found that the cells were unable to enter the CNS and primarily accumulated in the lungs, where they were completely cleared within 3 days (Figures 3F–3I). These findings showed that no hPSC-CNC PCs enter the CNS after intravenous transplantation. Therefore, we speculate that the therapeutic effects of hPSC-CNC PCs were not due to the cells directly repairing the BBB breaches but rather through an indirect way.

EVs-mediated function of hPSC-CNC PCs

Based on the results above, we speculated that the transplanted cells exerted their effects not through direct mechanisms such as filling vascular gaps but rather through the secretion of key bioactive components. Given that EVs are critical carriers of such substances—including cytokines, exosomes, and other signaling molecules—we further investigated the potential mechanisms by which EVs contribute following hPSC-CNC PC transplantation. We used size-exclusion chromatography (SEC) to isolate the main functional EVs from the cell supernatant and identified the characteristics of the EVs (Figure 4A). Nanoparticle tracking analysis (NTA) revealed that EVs from hPSC-CNC PCs ranged in size from 50 to 300 nm (Figure 4B). Western blotting results indicated that EVs were enriched with CD63, tumor susceptibility 101 (TSG101), and CD9 (Figure 4C). The transmission electron microscopy (TEM) image showed hPSC-CNC PC-derived EVs with a bilayer lipid structure (Figure 4D). To investigate the accumulation of EVs from hPSC-CNC PCs in 5xFAD mice, we labeled the extracted EVs with the Ev-Link505 kit and intravenously injected them into 5xFAD mice. The fluorescence signals were primarily observed in endothelial cells and neurons (Figure 4E). Furthermore, we treated mouse brain microvascular endothelial cells (bEnd.3) and hippocampal neuronal cells (HT22) with the PKH26-labeled EVs and found that these EVs could be taken up by HT22 and bEnd.3 cells (Figure 4F). We then tested the function of EVs in Aβ oligomer-treated cells. Western blotting results from HT22 and bEnd.3 cells indicated an increased expression of synaptic protein synaptophysin and the tight junction protein ZO-1 in EV-treated groups; in comparison, we did not observe an obvious increase in the cells treated with supernatant after removing the EVs (Figures 4G–4I).

Intravenous transplantation of hPSC-PC-derived EVs showed therapeutic effects similar to those of hPSC-CNC PCs in 5xFAD mice

To further investigate the function of EVs derived from hPSC-CNC PCs *in vivo*, we intravenously injected EVs into the retro-orbital veins of 12-month-old 5xFAD mice, twice per week for 2 weeks. The control group was injected with the same dose of PBS at the

Figure 2. hPSC-CNC PCs transplantation reduced BBB injury and promoted neurological functional recovery in 5xFAD mice

(A and B) Fluorescence intensity of fibrinogen in cortex and hippocampal CA1 region in each visible field. Immunofluorescence staining of fibrinogen revealed intravenous transplantation of hPSC-CNC PCs reduced BBB leakage in the cortex (top) and hippocampal CA1 region (bottom) of 5xFAD mice. (C) Two-photon imaging of the permeability index of the BBB. The intravenous transplantation of hPSC-CNC PCs reduced BBB leakage in 5xFAD mice, with less leakage of fluorescent dextran into the brain parenchyma. The permeability index was calculated as the area of fluorescent dextran versus the whole area of vessels in each visible field. (D) Nissl staining. The cell transplantation alleviated the degeneration of pyramidal neurons in the cortex of 5xFAD mice, as evidenced by the restoration of cell number (top) and morphology (bottom). (E and F) Western blotting analysis of endothelial and neuronal markers. The cell transplantation increased the expression of tight junction protein ZO-1 and synaptic proteins PSD95 and synaptophysin. (B–D and F) Data are presented as the mean ± SE. The experiment had no less than three independent biological replicates (one-way ANOVA). ns, $p \geq 0.05$; *$p < 0.05$; **$p < 0.01$; ***$p < 0.001$; ****$p < 0.0001$.

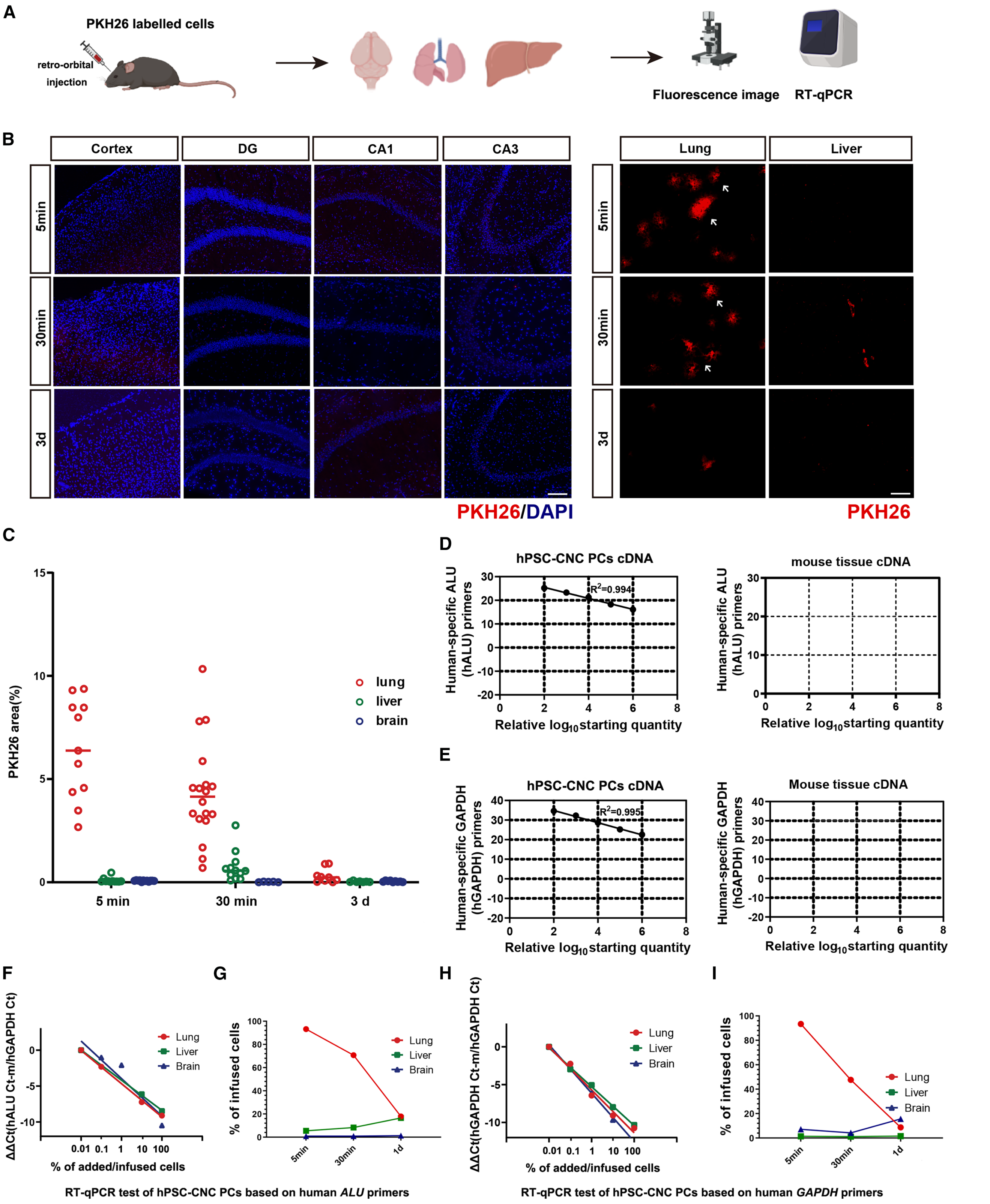

(legend on next page)

same time. Subsequently, we assessed the cognitive behaviors by Y-maze and NOR, and pathological changes, including Aβ deposition in 5xFAD mice (Figure 5A). Two weeks of EV injection did not affect the locomotion of 5xFAD mice (Figures S4A and S4B). In the Y-maze test, 5xFAD mice injected with EVs showed better Y-maze performance (Figure 5B, $P_{5xFAD+PBS\ vs.\ 5xFAD+PC} < 0.0001$). Similar results were shown in the NOR: after intravenous EV transplantation, 5xFAD mice showed better recognition index (Figure 5C, $P_{5xFAD+PBS\ vs.\ 5xFAD+PC} = 0.0003$). Furthermore, immunofluorescence staining revealed that Aβ deposition in the hippocampal CA1 region of 5xFAD mice decreased to varying degrees post-injection (Figures 5D and 5E), both in plaque number ($P_{5xFAD+PBS\ vs.\ 5xFAD+PC} = 0.0017$) and deposition area ($P_{5xFAD+PBS\ vs.\ 5xFAD+PC} = 0.0467$), while the number of Aβ plaque and the area of deposition in the cortex showed a decrease, but they did not reach statistical significance (plaque number: $P_{5xFAD+PBS\ vs.\ 5xFAD+PC} = 0.0722$; deposition area: $P_{5xFAD+PBS\ vs.\ 5xFAD+PC} = 0.1306$). No differences were shown in the Aβ deposition in the hippocampal DG region (Figures S4C and S4D). To sum up, these data revealed that the transplantation of EVs derived from hPSC-CNC PCs restored the cognitive behaviors of 5xFAD mice and reduced Aβ deposition.

We then performed immunofluorescence staining for fibrinogen and found that intravenous injection of EVs reduced BBB leakage in the cortex and hippocampal CA1 region (Figures 5F and 5G; CA1: $P_{5xFAD+PBS\ vs.\ 5xFAD+PC} = 0.0099$; cortex: $P_{5xFAD+PBS\ vs.\ 5xFAD+PC} = 0.0245$). We also observed recovery of neuron number through Nissl staining (Figures 5H and 5I; $P_{5xFAD+PBS\ vs.\ 5xFAD+PC} = 0.0003$). Moreover, western blotting analysis revealed that EV treatment increased ZO-1 expression (Figures 5J and 5K; cortex: $P_{5xFAD\ vs.\ 5xFAD+PC} = 0.0311$; hippocampus: $P_{5xFAD+PBS\ vs.\ 5xFAD+PC} = 0.0235$), as well as the expression of PSD95 (Figures 5J and 5K; cortex: $P_{5xFAD+PBS\ vs.\ 5xFAD+PC} = 0.0250$; hippocampus: $P_{5XFAD+PBS\ vs.\ 5xFAD+PC} = 0.0012$) and synaptophysin (Figures 5J and 5K; cortex: $P_{5xFAD+PBS\ vs.\ 5xFAD+PC} = 0.0691$; hippocampus: $P_{5xFAD+PBS\ vs.\ 5xFAD+PC} = 0.0102$).

To assess the safety of cell or EV injections, we monitored mouse body weight and survival at multiple time points. The survival rate was nearly 100% following cell or EV administration in our experiments (Figure S7). Moreover, no significant decrease in body weight was observed at any of the examined time points, including the day of injection and 1, 7, 14, and 28 days post-injection (Figure S8).

We also evaluated the peripheral immune response in mice following PC or EV administration (Figures S9 and S10). Flow cytometric analysis of immune cells revealed that after cyclophosphamide preconditioning, cell injection did not elicit a pronounced acute rejection response: lymphocytes, T cells, and natural killer cells remained unchanged (Figures S9A–S9H). Interestingly, however, macrophage numbers rose significantly by day 1 post-injection ($p = 0.0485$ vs. WT) and showed a trend toward increase on day 3 ($p = 0.1044$ vs. WT), suggesting that macrophages begin clearing PC aggregates in the lung (Figure S9I). Moreover, by day 3, polymorphonuclear myeloid-derived suppressor cells (PMN-MDSCs) decreased markedly ($p = 0.0019$ vs. WT), while monocytic MDSCs (M-MDSCs) ($p = 0.0068$ vs. WT), indicating that PC may influence MDSC subset dynamics (Figures S9J and S9K). In contrast, EV injection did not significantly alter any peripheral immune-cell subsets (Figures S10A–S10H), underscoring the superior safety profile of EVs relative to PCs. We also measured serum levels of IL-1, IL-4, IL-6, and IL-10 following cell or EV injection. Both treatments led to a significant reduction in IL-1 (PC: day 1 vs. WT, $p = 0.00196$; EV: day 3 vs. WT, $p = 0.0159$; Figures S9L and S10L), further demonstrating that our cell and EV treatments are minimally immunogenic and do not provoke cytokine storms.

miRNA-486-5p was highly enriched in EVs and demonstrated therapeutic effects in AD mice

EVs can carry a variety of biologically active components, including proteins, nucleic acids, and lipids. Among them, miRNAs play a significant role in regulating gene expression, facilitating intercellular communication, and are valuable for disease diagnosis and treatment. To further elucidate the potential downstream signaling pathways, we conducted miRNA sequencing on hPSC-CNC PC-derived EVs and identified miRNA-486-5p as the most highly expressed miRNA, with an abundance more than twice that of other miRNAs (Figure 6A; Table S1). Pathway enrichment analysis of miRNA-486-5p revealed its regulatory role in pathways associated with postsynaptic structure, postsynaptic density, and postsynaptic specialization (Figure 6B). RT-qPCR analysis of mouse brain tissue following intravenous cell or EV injection confirmed elevated

Figure 3. hPSC-CNC PCs failed to enter the mouse central nervous system after intravenous transplantation
(A) Experimental design (created with BioRender.com). Following intravenous transplantation, PKH26 fluorescence signal and human-specific primer analysis were used to track the hPSC-CNC PCs. (B and C) The tracking of PKH-26-labeled hPSC-CNC PCs after injection. Cells were labeled with PKH26, and the fluorescence intensity of PKH26 was quantified in each visible field of the brain tissue after cell transplantation. No PKH26 fluorescence signal was observed in the brain at 5 or 30 min or 3 days after cell injection, while in peripheral tissues, significant PKH26 fluorescence signal was detected in the lung tissue within minutes after cell injection. However, the cells were gradually cleared, nearly disappearing by day 3. (D and E) The specificity of human sequences. To confirm cell presence, human-specific sequences were also detected in both peripheral and brain tissues. The primers we used were human specific and were unable to recognize the *Alu* or *Gapdh* sequences of mice. (F and G) RT-qPCR test of hPSC-CNC PCs in tissues after injection based on human-specific *ALU* primers. Human-specific *ALU* sequence detection revealed that the cells were unable to enter the central nervous system and primarily accumulated in the lungs, where they were completely cleared within 3 days. (H and I) RT-qPCR test of hPSC-CNC PCs in tissues after injection based on human-specific *GAPDH* primers. Human-specific *GAPDH* sequence detection revealed that the cells were unable to enter the central nervous system and primarily accumulated in the lungs, where they were completely cleared within 3 days. (C) Data are presented as the mean ± SE. The experiment had no less than three independent biological replicates.

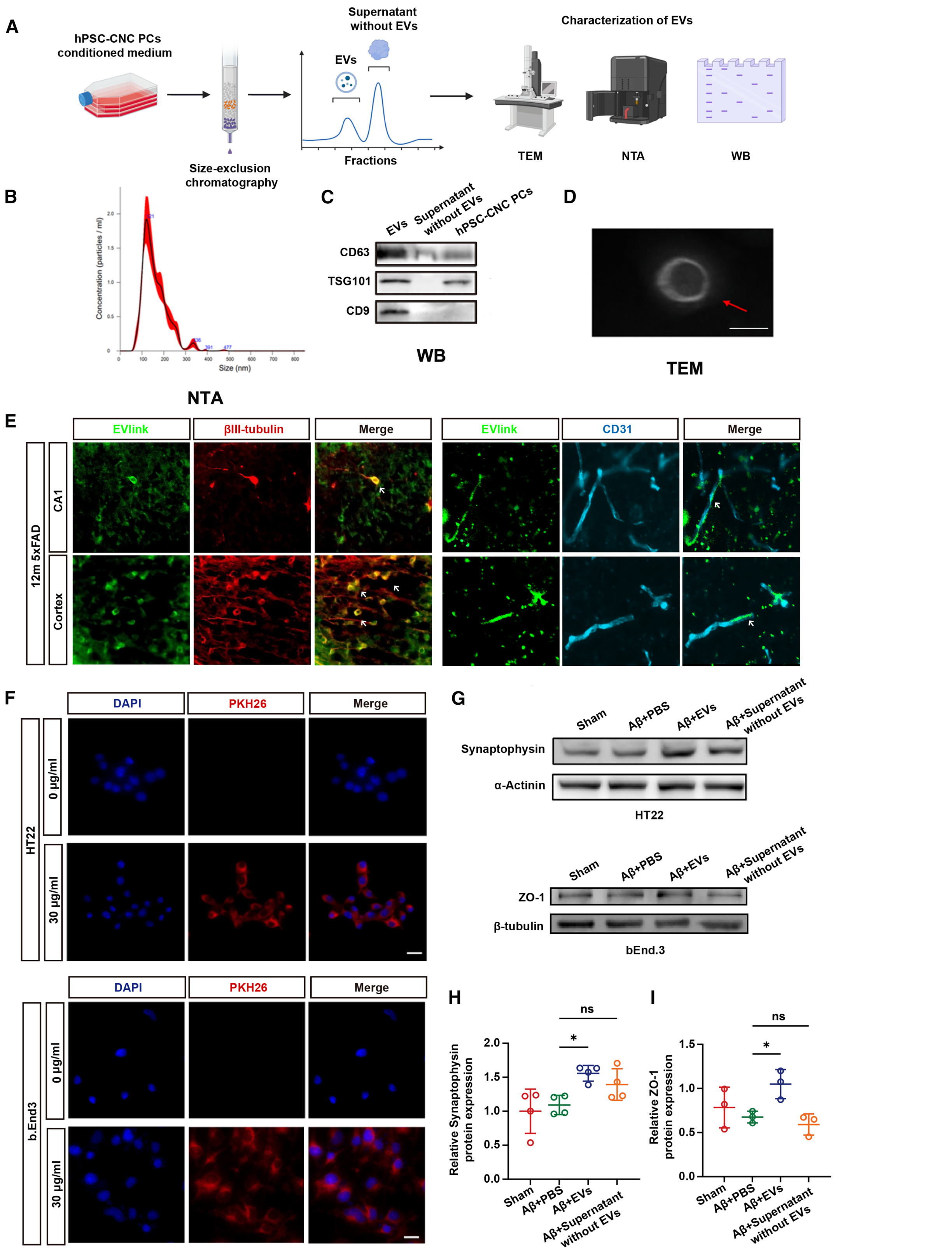

(legend on next page)

miRNA-486-5p levels (Figure 6C, $P_{5xFAD+PBS\ vs.\ 5xFAD+PC} = 0.0482$; $P_{5xFAD+PBS\ vs.\ 5xFAD+EVs} = 0.0425$). Given previous findings that EVs target neurons and endothelial cells, we treated HT22 and bEnd.3 cell lines with miRNA-486-5p mimics. Validation results demonstrated that the mimic significantly increased miRNA-486-5p levels in these cell lines (Figure S5A), and the mimic enhanced the expression of tight junction protein ZO-1 and neuronal synaptic protein synaptophysin (Figures 6D and 6E; $P_{A\beta+NC\ vs.\ A\beta+miRNA-486-5p} = 0.0254$). Furthermore, in an AD cell model treated with EVs, the therapeutic effect of EVs was significantly reduced by the addition of an miRNA-486-5p inhibitor (Figures 6F and 6G). These results confirmed that miRNA-486-5p was not only highly expressed in EVs but also acted as a key functional miRNA.

Based on this, we conducted a preliminary exploration of potential downstream signaling pathways. By retrieving references, we identified miRNA-486-5p-related signaling pathways (Figure S5B). Further RT-qPCR analysis of various molecules within these pathways revealed significant changes in *IGF1R* expression, indicating that this molecule may be a critical downstream target (Figures S5C and S5D). Western blotting analysis revealed a notable elevation in IGF1R protein expression within the brains of 5xFAD mice following either hPSC-CNC PC injection or EV injection (Figures 6H and 6I; $P_{5xFAD+PBS\ vs.\ 5xFAD+PC} = 0.0347$; $P_{5xFAD+PBS\ vs.\ 5xFAD+EVs} = 0.0189$). Considering these findings, we postulated that the miRNA-486-5p/IGF1R signaling pathway plays a pivotal role in the therapeutic efficacy of EVs.

To further investigate the function of miRNA-486-5p, we administered miRNA-486-5p agomir intranasally to 12-month-old 5xFAD mice, with a dosing frequency of once every 2 days for a total duration of 1 month. The NC group received the same dose of NC agomir at the same time. Subsequently, we assessed the behavior and pathological changes of the mice, including Aβ deposition (Figure 7A). A significant increase in miR-486-5p levels was observed in their brains, reaching a peak approximately 30 min after intranasal administration of miRNA-486-5p agomir (Figure 7B). Behavioral results indicated that miRNA-486-5p agomir improved Y-maze performance (Figure 7C; $P_{5xFAD+NC\ vs.\ 5xFAD+agomir} = 0.0038$) and NOR scores (Figure 7D; $P_{5xFAD+NC\ vs.\ 5xFAD+agomir} = 0.0115$) in 5xFAD mice. Immunofluorescence results showed a reduction in Aβ fluorescence intensity (CA1: $P_{5xFAD+NC\ vs.\ 5xFAD+agomir} = 0.0056$; cortex: $P_{5xFAD+NC\ vs.\ 5xFAD+agomir} < 0.0001$) and plaque number (CA1: $P_{5xFAD+NC\ vs.\ 5xFAD+agomir} = 0.0980$; cortex: $P_{5xFAD+NC\ vs.\ 5xFAD+agomir} = 0.0011$) in the cortex and hippocampus of 5xFAD mice after miRNA-486-5p agomir administration (Figures 7E, 7F, and S6). We also found that miRNA-486-5p agomir alleviated the degeneration of cortical pyramidal neurons (Figures 7G and 7H; $P_{5xFAD+NC\ vs.\ 5xFAD+agomir} = 0.0469$) and BBB leakage in cortex (Figures 7I and 7J; Cortex: $P_{5xFAD+NC\ vs.\ 5xFAD+agomir} = 0.0382$). The expression of Igf1r was also significantly decreased after agomir administration (Figure 7K; $P_{5xFAD+NC\ vs.\ 5xFAD+agomir} = 0.0493$). To ensure consistency and robustness of the findings, we conducted *in vitro* experiments in HT22 and bEnd.3 cells. Our results revealed that following incubation with Aβ oligomers, the levels of Igf1r protein significantly increased in both HT22 (Figure 7L; $P_{sham+NC\ vs.\ A\beta+NC} = 0.0305$) and bEnd.3 cell lines (Figure 7L; $P_{sham+NC\ vs.\ A\beta+NC} = 0.0116$). Moreover, the introduction of miRNA-486-5p mimics effectively reversed this upregulation, as evidenced by the results depicted in Figure 7L (HT22: $P_{A\beta+NC\ vs.\ A\beta+miRNA-486-5p} = 0.0433$; bEnd.3: $P_{A\beta+NC\ vs.\ A\beta+miRNA-486-5p} = 0.0462$). In addition, the RT-qPCR test found similar results: the levels of *Igf1r* mRNA significantly increased in both HT22 (Figure 7M; $P_{sham+NC\ vs.\ A\beta+NC} = 0.0015$) and bEnd.3 cell lines (Figure 7M; $P_{sham+NC\ vs.\ A\beta+NC} = 0.1330$), and miRNA-486-5p mimics effectively reversed this upregulation (Figure 7M; HT22: $P_{A\beta+NC\ vs.\ A\beta+miRNA-486-5p} = 0.0313$; bEnd.3: $P_{A\beta+NC\ vs.\ A\beta+miRNA-486-5p} = 0.0446$).These findings align well with those obtained from *in vivo* experiments, as well as with the outcomes of *in vivo* and *in vitro* intervention studies employing EVs. The results above indicated that the miRNA-486-5p/IGF1R signaling pathway might play a role in the neuroprotective and vascular repair effects conveyed by EVs from hPSC-CNC PCs.

DISCUSSION

Our study demonstrated that intravenous transplantation of hPSC-CNC PCs improved neuronal and endothelial cell function in a mouse model of advanced AD. In addition, we discovered that the therapeutic efficacy of EVs derived from these cells in AD. We also identified the role of miRNA-486-5p in improving neurovascular function.

Due to the lack of social awareness of AD, patients are often diagnosed at an advanced stage, when they have already lost the ability to move and care for themselves. At this stage, they are prone to complications such as infections, multi-organ failure, and even death,

Figure 4. Extracellular vesicles mediated function of intravenously transplanted hPSC-CNC PCs

(A) Procedure of the extraction and identification of extracellular vesicles (EVs) (created with BioRender.com). (B) Nanoparticle tracking analysis (NTA) of EVs. EVs from hPSC-CNC PCs ranged in size from 50 to 300 nm. (C) Western blotting of the markers of EVs. EVs were enriched with CD63, TSG101, and CD9. (D) The electron microscopy image of EVs. hPSC-CNC PC-derived EVs with a bilayer lipid structure. (E) Tracking of the EVlink505 labeled EVs after injection. The extracted EVs were stained using the EvLink505 kit and intravenously injected into 5xFAD mice, where fluorescence signals were primarily observed in endothelial cells and neurons. We marked some of the co-localization of EVlink with neurons (left) or endothelial cells (right) with white arrows. (F–I) The effect of EVs on HT22 and bEnd.3 cells *in vitro* experiments. Mouse brain microvascular endothelial cells (bEnd.3) and hippocampal neuronal cells (HT22) were treated with Aβ oligomers to induce Alzheimer disease (AD) cell models, followed by treatment with EVs derived from hPSC-CNC PCs. The results demonstrated that these EVs could be ingested by HT22 and bEnd.3 cells, as shown by red fluorescence signals surrounding the cell nuclei. (G–I) Western blotting test of synaptophysin and ZO-1 proteins in HT22 and bEnd.3 cells. EVs derived from hPSC-CNC PCs protected endothelial cells and neurons from Aβ oligomer treatment, as indicated by increased expression of the synaptophysin and the tight junction protein ZO-1, while other protein components from the hPSC-CNC PC supernatant, excluding EVs, showed no significant protective effect. (H and I) Data are presented as the mean ± SE. The experiment had no less than three independent biological replicates (one-way ANOVA). ns, $p \geq 0.05$; $^*p < 0.05$.

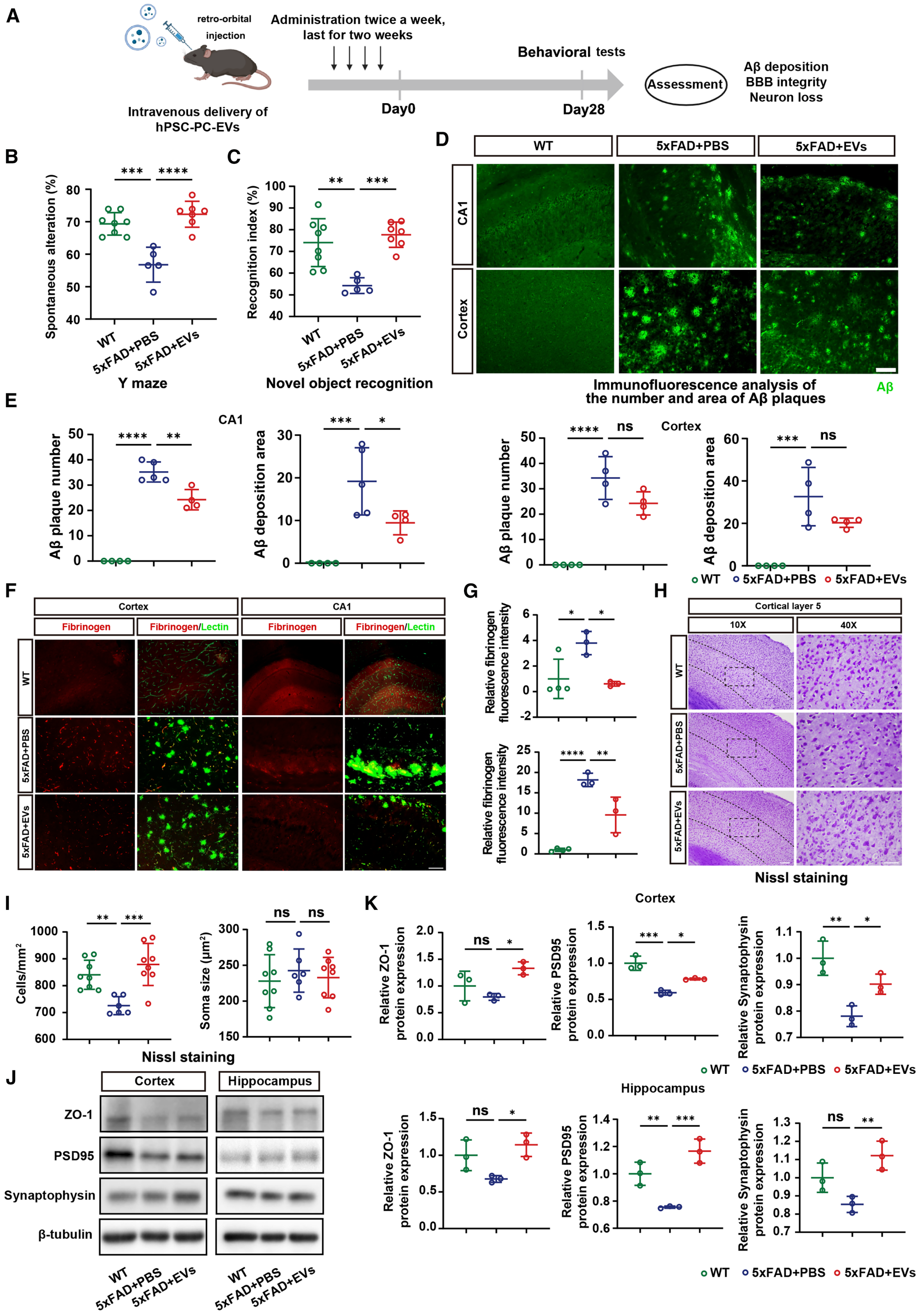

(legend on next page)

which imposes a significant economic burden on both society and families.[39,40] Moreover, recent efforts to develop clinical drugs targeting the main pathological features of AD, such as Aβ, have failed repeatedly.[41] Two anti-Aβ antibodies, aducanumab and lecanemab, have FDA approval, but concerns about their efficacy and safety persist. The approval of aducanumab in 2021 followed controversial data showing plaque clearance and some clinical benefits, despite earlier trial failures and risks like amyloid-related imaging abnormalities.[42,43] Lecanemab demonstrated clearer efficacy in phase 3 trials, with a 37% benefit in MCI but no effect in advanced AD. Current guidelines limit its use to early-stage AD.[44] These results highlight that while Aβ-targeted therapies may help prevent AD, they are ineffective in treating moderate-to-severe stages, stressing the need for new treatment strategies.

Therefore, our study focused on evaluating the therapeutic potential of hPSC-CNC PCs and their EVs in advanced AD. However, we did not explore their efficacy in earlier stages of AD, which will be the subject of future investigations. Our research further supported the therapeutic potential of targeting PCs and their EVs in treating advanced AD. In our study, we employed hPSC-CNC PCs, which were successfully induced following the protocol described by Sun et al.[13] Their research demonstrated that these cells support three-dimensional vascular network formation and regulate endothelial barrier function, establishing them as a robust model closely resembling human brain PCs. Moreover, hPSC-CNC PCs were shown to directly repair vascular defects in an MCAO mouse model, thereby alleviating cerebral ischemia-reperfusion injury.[13] Their study proposed the possibility of using peripheral transplantation of hPSC-CNC PCs as a treatment for neurological diseases. However, our results demonstrated that hPSC-CNC PCs did not across and directly repair the BBB in AD mice. This may be because compared with the acute damage of MCAO foci, the BBB of the AD mouse model chronically breaks down, resulting in a less compromised BBB that may prevent direct entry of circulating cells into the CNS. In addition, a study on the distribution of cells in different tissues in mice showed that intravenously transplanted MSCs accumulated mainly in the lungs and were rapidly cleared within a few days.[45] This supports our own findings.

Based on this, we further found that EVs derived from hPSC-CNC PCs have therapeutic effects in advanced AD mice. Research has demonstrated that EVs possess dual potential in the treatment of neurodegenerative diseases: they can act as both therapeutic agents and delivery vehicles for transporting active components, such as proteins and functional miRNAs, with surface markers that enable cell-specific targeting.[24] Compared to cell transplantation or traditional synthetic carriers, the ability of EVs to cross the BBB, its ease of storage, autologous origin, and low risk of immunogenicity and tumorigenicity[46,47] represent a readily available resource for clinical translation.

Previous studies have extensively demonstrated the therapeutic potential of stem cell-derived EVs. Among them, MSC-EVs have been the most thoroughly investigated. These EVs have been shown to selectively target hippocampal newborn neurons and microglial cells, thereby modulating neurogenesis, neuroinflammation, Aβ phagocytosis, and oxidative stress, processes that are critically involved in CNS disorders.[48–52] In our study, we found that EVs derived from stem cell-differentiated PCs specifically target endothelial cells and neurons. This finding underscores the therapeutic potential of stem cell-derived PC EVs for treating AD and highlights the possibility that EVs from different cellular origins may possess distinct targeting specificities. Consequently, comprehensive characterization of EV membrane receptor components is essential to elucidate the molecular mechanisms underlying their targeting behavior. Such investigations will advance our understanding of intercellular communication and support the development of novel, targeted drug delivery systems.

Currently, there is limited research on PC-EVs, and their specific mechanisms of action remain unclear. The existence of PC-EVs has been revealed only in recent years, with Gaceb et al. reporting their discovery in 2018.[53] However, it is only in the last 6 years that two studies focusing on brain PCs have effectively characterized the size and morphology of EVs.[20,54–56] The mechanisms underlying vascular and neuronal dysfunction associated with PC-EVs remain unclear, and the therapeutic efficacy of PC-EVs has not been confirmed in many neurodegenerative diseases. Our study investigated the functional properties of PC-EVs and demonstrated their potential as a novel therapeutic strategy for neurovascular injury-related disorders.

Our study delved into the mechanism of action of PC-EVs and discovered that they contained miRNAs that promoted angiogenesis,

Figure 5. Intravenous transplantation of EVs derived from hPSC-CNC PCs improved cognitive function, reduced Aβ deposition, reduced BBB injury, and promoted neurological functional recovery in 5xFAD mice

(A) Experimental design (created with BioRender.com). (B) Spontaneous alteration measured in Y-maze test. Intravenous transplantation of EVs derived from hPSC-CNC PCs improved Y-maze performance of the 12-month-old 5xFAD mice. (C) Recognition index measured in NOR test. Intravenous transplantation of EVs derived from hPSC-CNC PCs improved NOR performance of the 12-month-old 5xFAD mice. (D and E) Fluorescence intensity and plaque number of Aβ in cortex and hippocampal CA1 region in each visible field. Aβ deposition in the hippocampal CA1 region (left) and the cortex (right) of 5xFAD mice decreased to varying degrees post-transplantation, both in fluorescence intensity and plaque number. (F and G) Immunofluorescence staining for fibrinogen after EV injection. Intravenous injection of EVs reduced BBB leakage in cortex and hippocampal CA1 region. (H and I) Nissl staining. Intravenous injection of EVs induced the recovery of neuron number and morphology. (J and K) Western blotting analysis of endothelial and neuronal markers. EV treatment increased ZO-1 expression as well as the expression of PSD95 and synaptophysin in the cortex and hippocampal CA1 region of 5xFAD mice. (B and C) Data are presented as the mean ± SE. $N_{WT} = 8$, $N_{5xFAD+PBS} = 5$, $N_{5xFAD+EVs} = 6$ (one-way ANOVA). ns, $p \geq 0.05$; $^{**}p < 0.01$; $^{***}p < 0.001$; $^{****}p < 0.0001$. (E, I, and K) Data are presented as the mean ± SE. The experiment had no less than three independent biological replicates (one-way ANOVA). ns, $p \geq 0.05$; $^{**}p < 0.01$; $^{***}p < 0.001$; $^{****}p < 0.0001$.

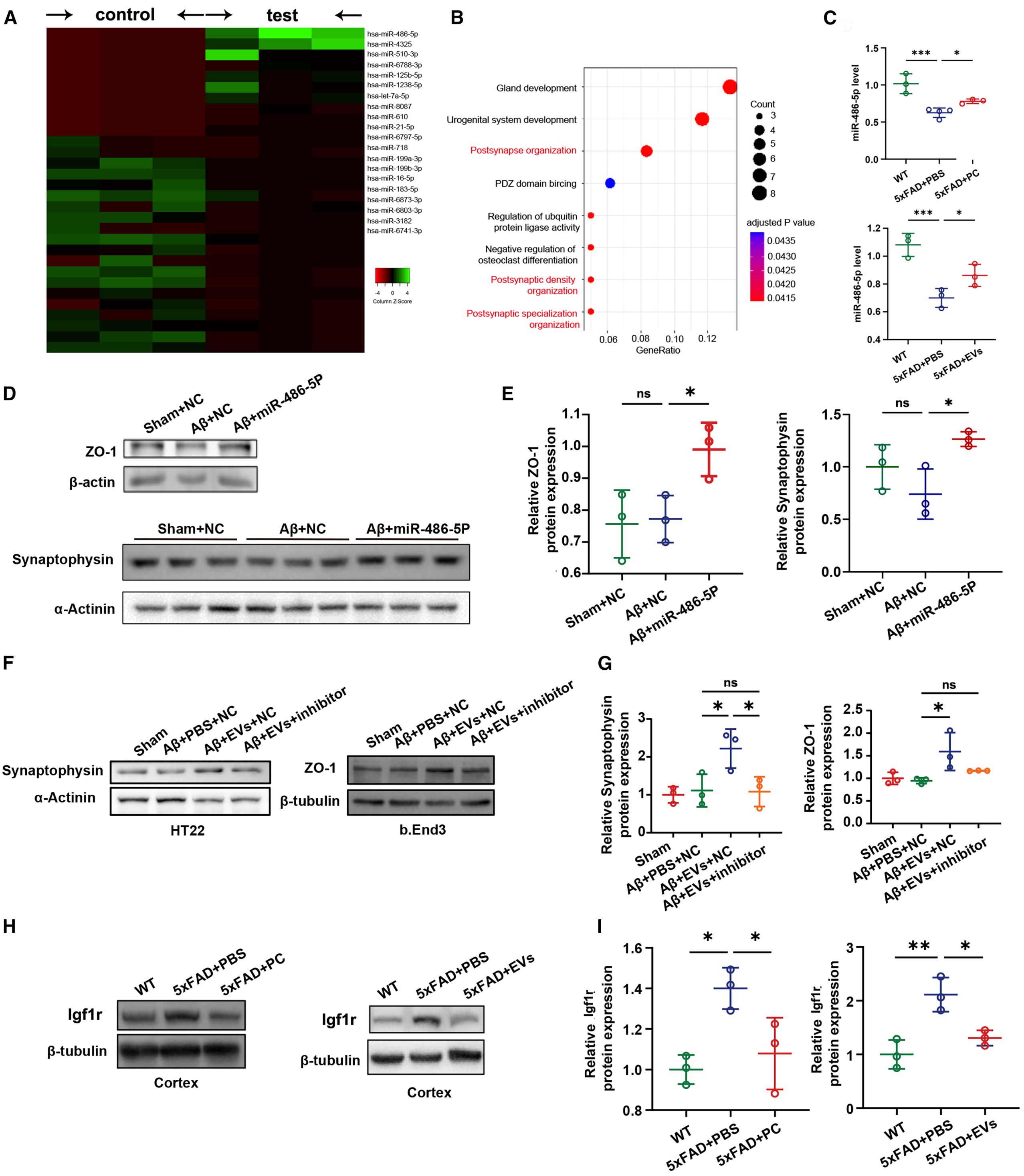

Figure 6. miRNA-486-5p was not only highly expressed in EVs derived from hPSC-CNC PCs but it also was a key functional miRNA within them

(A) miRNA sequencing of the control group (EVs extracted from exosome-free medium) and test group (EVs extracted from the supernatant of hPSC-CNC PCs). miRNA-486-5p was identified as the most highly expressed miRNA. Each group has three biological replicates. (B) Pathway enrichment analysis of miRNA-486-5p. miRNA-486-5p is involved mainly in pathways associated with postsynaptic structure, postsynaptic density, and postsynaptic specialization. (C) RT-qPCR analysis of mouse brain tissue for

(legend continued on next page)

BBB integrity, and neuronal survival. Among these miRNAs, has-miRNA-486-5p emerged as a potentially significant component of EVs. Preclinical investigations have revealed that miRNA-486-5p targets key signaling pathways involved in cell proliferation, migration, angiogenesis, and apoptosis regulation. Validated targets of miRNA-486-5p include phosphatase and tensin homolog (PTEN), forkhead box O1 (FOXO1), SMAD1/2/4, and IGF-1.[57]

miRNAs play a crucial role in neural activity by mediating processes such as cell proliferation, synaptic plasticity, and apoptosis. Currently, several potential miRNA targets for AD therapy have been identified in animal studies, including miR-124, miR-483-5p, miR-219, and miR-23/23b-3p.[29] In our study, we conducted intranasal delivery of miRNA-486-5p agomir and found that the delivery of miRNA-486-5p had a therapeutic effect similar to that of hPSC-CNC PCs isolated EVs. Previous studies have primarily focused on the role of miRNA-486-5p in diseases such as cancer.[58] There is limited research on the effects of miRNA-486-5p on the CNS. A clinical study demonstrated the enrichment of miRNA-486-5p in submucosal tissues of routine colon biopsies from Parkinson disease patients, suggesting its potential as a biomarker for the disease.[59] In addition, inhibition of endogenous miRNA-486-5p was found to activate PTEN and FOXO1, leading to the upregulation of the pro-apoptotic protein B cell lymphoma-2 interacting mediator of cell death and induction of cell death in glioblastoma multiforme cells.[58] Another animal study suggested that miRNA-486-5p may regulate neurogenesis, as observed by miRNA analysis of mouse cortical progenitor cells and neurons.[60] However, there is a lack of research on the expression, diagnostic utility, and therapeutic role of miRNA-486-5p in AD. Our study identified the potential of this miRNA to treat AD and reveal the mechanism of the miRNA-486-5p/IGF1R pathway in AD pathology.

miR-486-5p may protect the AD brain by inhibiting overactive IGF1R. Although IGF1R is essential in development, since IGF-1 binding triggers receptor autophosphorylation, insulin receptor substrate 1/2 (IRS1/2) recruitment and activation of neurotrophic pathways, its chronic activation in adulthood accelerates aging and neurodegeneration.[61,62] Lifelong reduction of IGF1R signaling in mice through heterozygous deletion or IRS2 knockout extends lifespan by approximately 20%–30%, enhances resistance to oxidative stress, and preserves metabolic health.[63,64] In AD models, IGF1R inhibition improves learning, lowers Aβ deposition, reduces glial activation, and restores neuronal and synaptic functions.[65–67] Pharmacological inhibitors such as picropodophyllin and NT219, together with our data showing that miR-486-5p downregulates IGF1R mRNA to reduce receptor levels, improve cognition and decrease Aβ pathology, support controlled IGF1R attenuation after development as a promising strategy to slow age-related decline and counter AD pathology.[68,69]

It is worth noting, however, that miRNA-based therapeutic strategies often involve off-target effects, which represents the primary obstacle that miRNA therapies must overcome to achieve clinical application. The multitargeting characteristics of miRNAs can easily result in adverse effects, as a single miRNA can bind to multiple transcripts, while a single mRNA may be targeted by various miRNAs.[70,71] Furthermore, miRNAs can target mRNAs that exert opposing effects within the same molecular pathway.[71] The relative endogenous abundance of miRNAs, as well as other "competing" miRNAs and their common targets in specific cells, can influence the extent to which a specific miRNA impacts its homologous mRNA.[72] Reliable identification of miRNA targets is a challenging task, and the high false positive rates associated with many target prediction algorithms complicate the matter further.[73] Therefore, EVs may have better potential in clinical translation due to their specific binding to certain cells.

Furthermore, it is important to note that EVs contain not only miRNAs but also lipids and proteins. Our study does not rule out the possibility that components other than EVs may also contribute to therapeutic effects. Additionally, our results showed that, alongside miRNA-486-5p, several other miRNAs were overexpressed. Although miRNA-486-5p was significantly more highly expressed than the other miRNAs, the roles of these miRNAs remain unclear. They may exhibit similar, opposing, or even synergistic effects. The previously mentioned off-target effects, the contributions of lipids and proteins, and the interactions between miRNAs may explain why EVs are more effective than miRNA agomir treatments alone. Future multi-omics analyses could help establish the expression profile of PC-EVs and clarify the signaling networks that underlie their therapeutic effects. The future integration of artificial intelligence with multi-omics analysis, single-cell spatial transcriptomics, chemical modifications, and optimized delivery systems may facilitate the determination of the expression profile of PC-EVs, elucidate the signaling networks underlying their therapeutic effects, and minimize off-target effects and toxicity.

In addition, several limitations must be addressed for the clinical application of PC-EVs in the future. In this study, we employed a fixed-dose administration strategy without body weight adjustment in mice. Currently, there is no consensus on whether weight-based dose correction is necessary in cell/EV therapy research for CNS diseases. Most preclinical studies adopt weight-independent fixed

miRNA-486-5p. Elevated miRNA-486-5p expression was found after hPSC-CNC PCs (left) or EV injection (right). (D and E) Western blotting test of synaptophysin and ZO-1 proteins in HT22 and bEnd.3 cells with treatment of negative control (NC) or miR-486-5p mimics. In HT22 and bEnd.3 cell lines treated with Aβ, the miRNA-486-5p mimics significantly enhanced the expression of tight junction protein ZO-1 and neuronal synaptic protein synaptophysin. (F and G) Western blot detection of synaptophysin and ZO-1 proteins in HT22 and bEnd.3 to verify the key role of EVs. In AD cell models treated with EVs, the therapeutic effect of EVs was significantly reduced by the addition of an miRNA-486-5p inhibitor, confirming that miRNA-486-5p has the possibility to be a key functional miRNA. (H and I) Western blotting test of Igf1r protein within the brains of 5xFAD mice following hPSC-CNC PC or EV injections. The Igf1r protein levels were decreased following either hPSC-CNC PC injection or EV injection. (C, E, G, and I) Data are presented as the mean ± SE. The experiment had no less than three independent biological replicates (one-way ANOVA). ns, $p \geq 0.05$; $^{*}p < 0.05$; $^{**}p < 0.01$; $^{***}p < 0.001$.

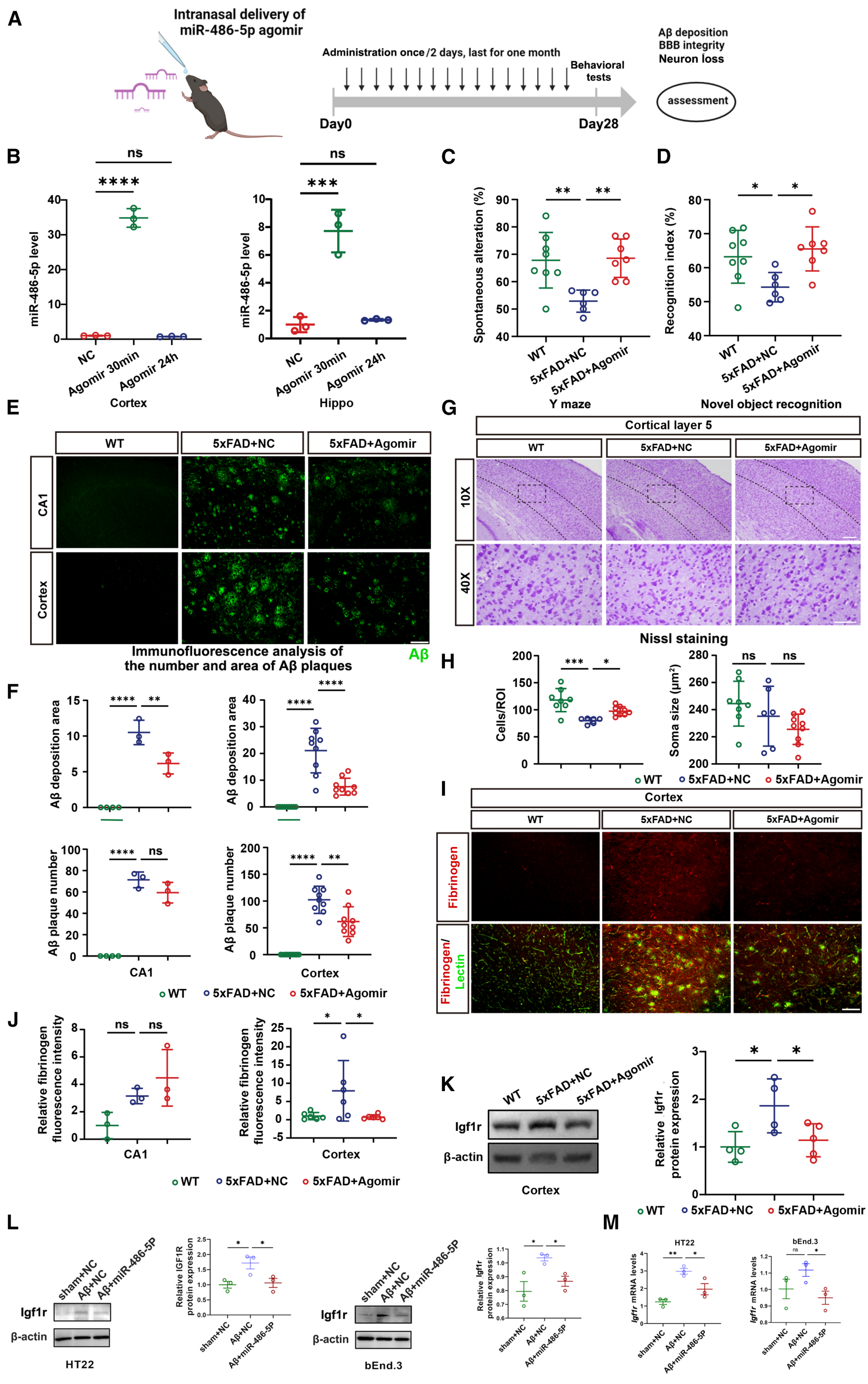

(legend on next page)

dosing regimens, likely due to the complex dose-response relationships of cell-based therapeutics and interindividual variability in heterologous cell engraftment efficiency. It is crucial to emphasize that establishing a scientific dose standardization system is paramount for clinical translation. Future studies should systematically evaluate the influence of body weight, metabolic rate, immune function, and BBB penetration efficiency on cell/EV therapy, including pharmacokinetic investigations such as developing physiologically based pharmacokinetic modeling prediction systems.[74] Additionally, dose translation from animal studies to human trials and therapeutic efficacy validation should be explored. These efforts will help provide a theoretical foundation for developing personalized treatment regimens. To summarize, standardized isolation and purification methods, efficient dosing and safety protocols, as well as standards for potency and final product quality control are essential prerequisites. Additionally, like EVs derived from other sources, the adverse effects of PC-EVs remain unclear. It is crucial to characterize the composition of PC-EVs under both physiological and pathological conditions to better understand the progression of neurodegenerative diseases. Overcoming these limitations will pave the way for the utilization of PC-EVs as potential therapeutic agents for vascular and neurological diseases.

MATERIALS AND METHODS

Mice model of AD

All experimental procedures involving animals received approval from the Sun Yat-Sen University Animal Utilization and Care Committee (approval no. BAP20230069). For this study, we used male 5xFAD mice, aged 12 months, and age-matched male WT mice procured from The Jackson Laboratory.[75] All mice were accommodated in a controlled environment following standard specific pathogen-free conditions, including regulated temperature, humidity, and a 12-h light/dark cycle, and provided with unrestricted access to food and water.

PC-like cell differentiation from hPSCs

We generated human induced PSC (hiPSC) lines by transducing human embryonic fibroblasts with a lentiviral vector expressing the reprogramming factors octamer-binding transcription factor 4, Kruppel-like factor 4, SOX2, and myelocytomatosis viral oncogene homolog.[76] hPSC-PCs were then differentiated from hiPSC induced CNC according to previous research.[13] The step-by-step protocol describing the differentiation process can be found at Protocol Exchange.[77] In brief, the differentiation in hPSCs was induced through a chemical small-molecule induction system. Subsequently, HNK1$^+$ p75bright cells were purified via flow cytometry sorting. The culture was then switched to commercial Pericyte Medium (ScienCell) containing 10 ng/mL basic fibroblast growth factor and 50 ng/mL PDGF-BB for 7–14 days to induce CNC differentiation into cerebral microvascular PCs (CNC-PCs). *In vitro* experiments convincingly demonstrated that CNC-PCs expressed specific PC markers, including NG2, PDGFRβ, and CD13. Moreover, these cells exhibited the characteristic abilities of PCs, such as contraction and tube formation. Significantly, CNC-PCs have been proved to play a pivotal role in enhancing the barrier function of endothelial cells, evident in the increased transmembrane resistance and upregulation of tight junction protein expression in previous research.[13]

Transplantation and tracking of hPSC-derived PCs

After hPSC-CNC PCs were successfully differentiated from CNC as previously described,[13] we labeled the cell membranes of hPSC-CNC PCs with PKH26 dye, which produces red fluorescence. The cells were then transplanted into mice via retro-orbital injection. To prevent pulmonary embolism, sodium was diluted to a concentration of 100 U/kg in suspension and co-administered with cells via retro-orbital injection.[78] According to the previous protocol, cyclosporine A (CSA, 10 mg/kg/day body weight, Sandoz) was administered subcutaneously to inhibit the immune rejection of transplanted cells.[79] To monitor the distribution of transplanted PKH26-labeled (Sigma-Aldrich) hPSC-CNC PCs in mice, the livers, lungs, kidneys, and brains were processed into frozen sections at 5 min, 30 min, and 3 days post-transplantation. These sections underwent fixation, washing, and incubation with 4′,6-diamidino-2′-phenylindole (DAPI). Subsequently, all sections were imaged using a fluorescence microscope (Olympus BX63). In addition, to determine cell distribution within tissues, varying amounts of hPSC-CNC PCs were

Figure 7. miRNA-486-5p has a therapeutic effect on attention-deficit/hyperactivity disorder mice, and miRNA-486-5p/IGF1R signaling might play a role in the protected effects mediated by EVs

(A) Experimental design (created with BioRender.com). (B) RT-qPCR test of miRNA-486-5p levels after miRNA-486-5p agomir administration. A significant increase in miR-486-5p level was observed in the brains of the mice, reaching a peak approximately 30 min after intranasal administration of agomir. (C) Spontaneous alteration measured in Y-maze test. Intranasal delivery of miRNA-486-5p agomir improved Y-maze performance of the 12-month-old 5xFAD mice. (D) Recognition index measured in the NOR test. Intranasal delivery of miRNA-486-5p agomir improved NOR performance of the 12-month-old 5xFAD mice. (E and F) Fluorescence intensity and plaque number of Aβ in cortex and hippocampal CA1 region in each visible field. Aβ deposition in the hippocampal CA1 region (left) and the cortex (right) of 5xFAD mice decreased to varying degrees after delivery of miRNA-486-5p agomir, both in fluorescence intensity and plaque number. (G and H) Nissl staining. Intranasal delivery of miRNA-486-5p agomir alleviated the degeneration of cortical pyramidal neurons, shown by the recovery of neuron number and morphology. (I and J) Fluorescence intensity of fibrinogen in cortex and hippocampus in each visible field. Intranasal delivery of miRNA-486-5p agomir alleviated the BBB leakage, particularly in the cortex. (K) Western blotting test of Igf1r protein within the brains of 5xFAD mice following miRNA-486-5p agomir administration. The Igf1r protein levels within the brains of 5xFAD mice were decreased following intranasal delivery of miRNA-486-5p agomir. (L) Western blotting test of Igf1r protein of HT22 and bEnd.3 cells with treatment of NC or miRNA-486-5p mimics. Aβ oligomers can increase the expression of Igf1r in cells, while miRNA-486-5p mimics reverse this change. (M) RT-qPCR test of *Igf1r* mRNA of HT22 and bEnd.3 cells with treatment of NC or miRNA-486-5p mimics. Aβ oligomers can increase the levels of *Igf1r* mRNA in cells, while miRNA-486-5p mimics reverse this change. (C and D) Data are presented as the mean ± SE. $N_{WT} = 8$, $N_{5xFAD+NC} = 6$, $N_{5xFAD+agomir} = 7$ (one-way ANOVA). ns, $p \geq 0.05$; *$p < 0.05$; **$p < 0.01$. (B, F, H, and J–M) Data are presented as the mean ± SE. The experiment had no less than three independent biological replicates (one-way ANOVA). ns, $p \geq 0.05$; *$p < 0.05$; **$p < 0.01$; ***$p < 0.001$; ****$p < 0.0001$.

injected and human-specific *ALU* and *GAPDH* mRNA were detected by RT-qPCR (catalog no. ABI7500, Thermo Fisher). Separate standard curves were generated for each tissue to reduce variability due to differences in the amount of DNA extracted, the number of cells in the organ, or the efficiency of the PCR. The assay had a sensitivity of approximately 100 human cells per mouse organ. RT-qPCR analysis of *ALU* sequences was conducted in a 50-μL reaction volume, comprising 25 μL TaqMan Universal PCR Master Mix, 900 nM forward and reverse primers, 250 nM TaqMan probe, and 200 ng target template. Similarly, RT-qPCR for human and mouse *GAPDH* genes was performed in a 50-μL volume, using 25 μL SYBR Green Master Mix, 200 nM forward and reverse primers, and 200 ng target template (primer and probe sequences are detailed in Table S2). The reaction mixture was incubated at 50°C for 2 min, followed by an initial denaturation at 95°C for 10 min, then subjected to 40 cycles of 15 s at 95°C and 1 min at 60°C. All RT-qPCR assays were conducted in duplicate or triplicate, with the average values reported. To correct the final value of total DNA in the sample, primers were used that simultaneously amplify the human and mouse *Gapdh* genes. Specific procedural details were consistent with those described in previous studies.[45]

In the cell therapy group, 1×10^6 cells, suspended in 400 μL PBS (ServiceBio), was administered intravenously to each mouse at biweekly intervals over a span of 4 weeks to evaluate therapeutic efficacy, a dosage guided by those utilized in intravenous MSC administration for AD mouse models.[80,81] Control group mice were intravenously transplanted with an equivalent volume of PBS. CSA (Solarbio) was administered subcutaneously following previous protocols[13] to mitigate immune rejection of the transplanted cells.

Isolation, transplantation, and tracking of EVs

hPSC-PCs were cultured in a specialized PC medium (ScienCell). This medium comprises a basal PC medium supplemented with PC growth supplement, fetal bovine serum (FBS), and a penicillin/streptomycin (P/S) solution. EV-removed serum was prepared by subjecting intact FBS to ultracentrifugation at 100,000 × *g* for more than 16 h using an ultracentrifuge (Beckman), resulting in EV-removed PC medium, which was then filter sterilized using 0.22-μm filters (NEST).

After seeding the cells, the culture medium was replaced with EV-removed PC medium when the cell confluence reached 70%–80%. The supernatant was collected following a 48-h incubation, and EV extraction was performed using qEV SEC columns (IZON) according to the manufacturer's guidelines.[82] Quantification and characterization of EVs were performed by BCA kits (Beyotime), protein blotting, NTA, and TEM.

EVs were labeled using EvLINK505 (TINGOscience), a fluorescent probe chemically coupled to the outer surface of EVs and emitting stable and bright green fluorescence with an excitation wavelength of 505 nm and an emission wavelength of 535 nm, followed by intravenous injection of the labeled EVs at a dose of 50 μg dissolved in 200 μL PBS per mouse. To prevent pulmonary embolism, sodium was diluted to a concentration of 100 U/kg in suspension and co-administered with EVs via retro-orbital injection.[78,83] Mice were euthanized 1 day post-injection, and frozen brain tissue sections were obtained for observation. We then examined the localization relationship between EvLINK505 signals and $CD31^+$ endothelial cells and β-tubulinIII+ neurons. For the *in vitro* EV uptake experiment, we incubated HT22 or bEnd.3 cells with 30 μg PKH26-labeled EVs for 24 h, then observed the uptake using a fluorescence microscope.

Sequencing of hPSC-PC supernatant exosomal miRNAs

Sequencing of hPSC-PC supernatant exosomal miRNAs was conducted by Wayen Biotech using the QIAseq miRNA Library Kit, a next-generation sequencing (NGS) library kit designed for accurate miRNA quantification through second-generation sequencing (NGS). The library construction process involved several steps: ligation, cDNA synthesis, and library amplification. In ligation, pre-adenylated 3′ and 5′ junctions were sequentially added to the miRNA by ligase. Subsequently, reverse transcription was performed to convert the miRNA into cDNA using reverse transcription primers containing unique molecular identifier (UMI)-tagged sequences at the 3′ junction. Library amplification utilized universal forward and reverse primers, with the amplified products purified using magnetic beads for quality control analysis and sequencing. Library construction can be completed within 7 h, with UMIs introduced during the reverse transcription reaction.

The data analysis process was as follows: the Cutadapt software (version 2.7) was used to process low-quality, high N content sequencing junctions and short reads below 16 bp. Subsequently, reads with UMIs shorter than 12 bp and those without UMIs were processed using perl5 (version 26) and UMIs were identified. Next, bowtie software (version 1.3.0) was used to compare the reads with the miRBase, piRNAbank, and Rfam databases, respectively. Finally, reads were compared to miRBase, piRNAbank, and Rfam databases for small RNA quantification.

The top 50 miRNAs were annotated for target gene prediction using miRTarBase, an experimentally validated miRNA target gene interaction database. Gene Ontology enrichment analysis identified significant functions associated with trait changes, while Kyoto Encyclopedia of Genes and Genomes provided insight into biological functions from a genomic perspective. Enrichment analyses were conducted using the Fisher exact test with the clusterProfiler data package from R/Bioconductor.

Nasal administration of miRNA agomir and target engagement validation

The intranasal administration of MicrON hsa-miRNA-486-5p agomir (GeneID: MIMAT0002177, RiboBio) or agomir-NC was conducted following established protocols.[84] The hsa-miRNA-486-5p agomir, dissolved in 24 μL RNase-free water (2 nmol), was delivered in 4-μL drops (a total of 6 times) through a pipette, alternating

between each nostril every 2−3 min. The mice's respiration remained unobstructed, and no signs of other ailments were noted; all mice survived. The administration occurred every other day over a span of 28 days. Subsequently, mice underwent behavioral testing.

Cell culture and treatment

Amyloid β 1–42 ($A\beta_{1\text{-}42}$) oligomers were prepared according to established methods.[85] Frozen $A\beta_{1\text{-}42}$ lyophilized powder (GenScript) was injected into hexafluoroisopropanol (HFIP, Macklin), vortex mixed thoroughly, and allowed to stand at room temperature for 60 min until the liquid became clear, resulting in the formation of the Aβ-HFIP solution (1 mM). Subsequently, the Aβ-HFIP solution was aliquoted and the HFIP was dried using a vacuum freeze dryer (Labconco FreeZone 2.5 L) to produce a colorless and transparent Aβ peptide film. Prior to usage, the peptide was mixed with dimethyl sulfoxide (DMSO, Sigma-Aldrich), and subjected to a 10-min water bath ultrasonic treatment (300 W, 35 Hz) to obtain the Aβ-DMSO solution (5 mM). Finally, the Aβ-DMSO solution was mixed with pre-cooled PBS solution through vortexing. The resulting solution was incubated in a 4°C refrigerator for 24 h to obtain the Aβ oligomer solution (100 μM).

HT22 cells and bEnd.3 cells (Hunan Fenghui Biotechnology) were maintained in DMEM supplemented with 10% heat-inactivated FBS (Procell) and 1% P/S (Thermo Fisher). Cells were incubated at 37°C in a saturated humidified atmosphere containing 5% CO_2. When HT22 or bEnd.3 cells reached 70%–80% confluence, the culture medium was replaced with Opti-MEM medium (Thermo Fisher) and 5 μM of Aβ oligomers were added, while also adding EV (10 μg/mL) or protein components (corresponding to the residual protein content of the EV-extracted supernatant). Western blotting experiments were performed 24 h after change of medium.

For experiments involving miRNA mimics, when HT22 or bEnd.3 cells reached 70%–80% confluence, the culture medium was replaced with Opti-MEM medium. Aβ oligomers were added to create the AD cell model, while also adding 50 nmol miRNA mimics or the same dose of NC mimics with the incubation of Aβ. Subsequently, proteins and RNA were extracted for western blotting or RT-qPCR detection. For experiments involving miRNA inhibitors, in the AD cell model, 10 μg/mL EVs and 200 nmol miRNA inhibitors were added concurrently with the incubation of Aβ. After 24 h, the Western blotting experiments were conducted.

All the primers used for RT-qPCR are listed in Table S2, and the primary antibodies used in western blotting are listed in Table S3. The expression of the endothelial cell tight junction protein ZO-1, the neuronal marker proteins PSD95 and synaptophysin, as well as the downstream target IGF1R were analyzed using ImageJ software (NIH).

Behavioral tests

Neurological functional deficits were assessed on the 14th and 28th day following cell administration. All behavioral tests were conducted under blinded conditions. For 3 days prior to all behavioral studies, mice underwent daily handling. Three main behavioral tests were conducted in this study: the open field test, the Y-maze spontaneous alternation test, and the NOR test.

The open field test utilizes the natural preference of animals to explore novel environments and assesses the spontaneous behavior of experimental animals in open, novel environments. The dimensions of the open field for mice were 50 cm in length, 50 cm in width, and 40 cm in height. The mouse was gently removed from its cage and quickly placed in the central area of the experiment chamber. An automated animal behavior analysis system (CleverSys) was then used to automatically record the mouse's activity in the box, typically over a 10-min period. The time spent by the mouse in the center and corner was quantified. Cases where the time spent in the center was insufficient or the time spent in the corners was excessive may indicate that the mouse was experiencing anxious or depressive emotions. The experiment chamber and objects were cleaned with 70% ethanol to reduce the influence of mouse odor between the trials. The time spent in the center and corners was quantified using the automated animal behavior analysis system (CleverSys).

The Y-maze spontaneous alternation test utilizes the natural preference of mice to explore novel environments to assess working memory. The Y-maze comprises three black opaque plastic arms (40 cm in length, 10 cm in width, and 12 cm in height), each forming a 120° angle with the others. Once positioned at the center of the maze, the mouse was given 10 min to freely explore the three arms. To minimize the influence of mouse odor between trials, the maze was cleaned with 70% ethanol. A complete entry of the mouse's tail into one of the maze arms was recorded as an instance of entry. The criterion for a successful alternation was fulfilled when the mouse made three entries into different arms. The alternation rate (%) was calculated by dividing the number of successful alternations by the total number of entries minus 2.

To study long-term memory in mice, we administered a NOR test according to a previously documented protocol.[86] Mice were housed for 3 days, during which they underwent a familiarization phase on day 1, where they spent 10 min in a square chamber (50 × 50 × 40 cm) with no objects. Then, on day 2, the mice encountered two identical objects within the chamber and were given a 10-min exploration window. On day 3, one of the familiar objects was replaced with a novel object and the animals were given another 10-min period of unrestricted exploration. Mice that failed to explore both objects for at least 20 s on either day 2 or 3 were excluded from the analysis. Throughout the study, the behaviors of the mice in the chamber were documented using an overhead camera. Exploration time on days 2 and 3 was assessed by a trained observer blinded to the experimental groups. To minimize the influence of mouse odor between trials, the chamber and experimental items were cleaned with 70% ethanol. Exploration time was quantified as the cumulative time that mice spent sniffing each object within a 2-cm

radius, with their noses directed toward the objects. The recognition rate was calculated by dividing the time spent sniffing the novel object by the total time spent exploring both objects.

Assessing pathology, BBB integrity, and neuronal function in treated mice

At 28 days after intravenous transplantation of cells or nasal administration of miRNA-related drugs, or 2 weeks after transplantation of EVs, pathological deposits, endothelial cells, and neuronal changes in the mice brains were assessed to further evaluate the treatment effects.

Immunofluorescence staining with Aβ mOC64 antibody (Abcam) was used to detect Aβ deposits in the mouse brains. The integrity of the BBB was evaluated using immunofluorescence staining with fibrinogen antibody (Solarbio) and two-photon imaging. For immunofluorescence staining, the mice were anesthetized with isoflurane and transcardially perfused with ice-cold PBS. The brains were dissected and fixed in 4% paraformaldehyde, and then sliced into 20-μm-thick sections using a vibratome (Leica CM1950). The slices were blocked with a buffer containing 0.3% Triton X-100 (Sigma-Aldrich), 5% normal donkey serum (catalog no. SL050, Solarbio), and 5% BSA (Sigma-Aldrich) for 2 h at room temperature, before being incubated with primary antibodies at 4°C overnight. Secondary antibodies (1:1,000 dilution) were incubated for 2 h at room temperature. The primary antibodies used are listed in Table S4. The nucleus was stained with DAPI (Cell Signaling Technology) for 30 min at room temperature. Imaging was performed with an Olympus BX63 microscope. Quantitative image analysis, including Aβ deposition area, Aβ plaque number, $CD13^+$ PC coverage, $CD31^+$ or $lectin^+$ vascular length, $Iba1^+$ microglia area, $GFAP^+$ astrocyte area, PKH26 labeling area, and fluorescence density of fibrinogen, was conducted on maximum projections of 10-μm-thick z stack images. The analysis was performed by investigators who were blinded to the treatment groups using ImageJ software.

For two-photon imaging, fluorescent dyes with different molecular weights (Table S6) were injected intravenously at a concentration of 10 mg/mL (0.1 mL/mouse). Detectable BBB leakage of 4 kDa tetramethylrhodamine-dextran or 70 kDa fluorescein isothiocyanate-dextran was observed in the mice after behavior tests. BBB permeability measurements were performed on cortical vessels using the open skull method.[87] Images were obtained using a two-photon microscope (Olympus, catalog no. FV31S) with a 1.05 numerical aperture 25× objective immersed in artificial cerebrospinal fluid and the laser tuned to 850 nm. Vessel images of the cerebral cortex 50 μm below the pial surface were obtained every 2 min for a total of 30 min after dye injection in each mouse. Regions of interest (ROIs) were stochastically selected 50 μm below the pial surface throughout the brain parenchyma for fluorescence intensity measurement. Increasing fluorescence signal of ROIs over the first projected z stack (baseline) in the time-lapse recording indicated that fluorescent dyes circulating in the blood had leaked into the brain parenchyma.[87] The permeability index was calculated as the area of fluorescent dextran versus the whole area of vessels in each visible field as previously reported.[88] Quantification was performed by an investigator blinded to the experimental design using ImageJ software.

In addition, changes in ZO-1, an endothelial tight junction marker, and synapse-related proteins such as PSD95 (Abcam) and synaptophysin (Abcam), which respond to changes in synaptic function, were detected by western blotting (Thermo Fisher, catalog no. 40-2210). To detect neuronal changes in the mouse brain, neuronal number and morphological changes were assessed by Nissl staining (Solarbio) according to the product instructions.

Detection of the immune system after cell or EV injection

ELISA for cytokine quantification

Cytokine levels in serum were measured using a commercial ELISA kit (R&D Systems) according to the manufacturer's instructions. Briefly, serum samples were obtained from each mouse before cell or EV injection (WT group), as well as 1 and 3 days following injection. These samples were promptly frozen and stored at −80°C, awaiting further analysis. The cytokine panels included IL-1, IL-4, IL-6, and IL-10 (all from Thermo Fisher). For the assay, 96-well plates were coated overnight with the appropriate capture antibody (1 μg/mL) at 4°C. After blocking with 1% BSA in PBS for 1 h at room temperature, the plates were incubated with serum samples (diluted 1:2 in assay buffer) for 2 h at room temperature. After washing, biotinylated detection antibodies (0.5 μg/mL) were added for 1 h at room temperature, followed by streptavidin-horseradish peroxidase (1:1,000 dilution). Substrate solution was added, and the absorbance was measured at 450 nm using a microplate reader (BioTek). Data were analyzed using Gen5 software (BioTek) and are expressed as pg/mL. The minimum detectable concentration for each cytokine was <10 pg/mL. The intra-assay coefficient of variation was <10% for all cytokines measured.

Fluorescence-activated cell sorting for immune cell profiling

Peripheral blood was acquired from each mouse before cell or EV injection (WT group), as well as 1 and 3 days following injection, and were stored in EDTA-coated tubes. We aliquoted 200 μL blood and stored it at 4°C for up to 48 h. For the experiment, we transferred 50 μL blood into a flow cytometry tube and then added 0.1 μL Zombie NIR and 0.5 μL TruStain FcX PLUS (anti-mouse CD16/32). This was incubated in the dark for 10 min without washing. Next, we added 1 μL F4/80 antibody and incubated it in the dark for an additional 15 min. Following this, we added 2 mL PBS, centrifuged at 350 × *g* for 5 min and discarded the supernatant. We added 50 μL Brilliant Stain Buffer to each tube, followed by the sequential addition of 0.25 μL CD45, 0.5 μL CD3, 0.5 μL CD4, 1 μL CD8, 0.5 μL CD49b, 0.25 μL CD11b, 0.25 μL Ly-6G, and 0.5 μL Ly-6C antibodies. We vortexed to mix and incubated it in the dark for 15 min. Subsequently, we added 2 mL of BW01 lysing buffer, vortexed thoroughly, and incubated it at room temperature for 10 min. We centrifuged at 350 × *g* for 5 min and discarded the supernatant. We resuspend the cells in 2 mL PBS, centrifuged again at 350 × *g* for 5 min,

discarded the supernatant, and finally, resuspended the cells in 200 μL PBS for analysis on a flow cytometer. Blank tubes and single-stain controls (including mCD45-FITC, mCD3-APC-Cy7, mCD4-BV786, mCD8-BV605, mCD49b-PE, m/hCD11b-AF700, mLy-6C-BV421, mLy-6G-PE/Cy5, mF4/80-APC, and Zombie NIR) were included to perform unmixing, ensuring the accuracy and reliability of the results.

Flow cytometry was performed using a BD LSRFortessa flow cytometer (BD Biosciences), and data were analyzed using FlowJo version 10 (TreeStar). The gating strategy included the following steps: singlets were selected based on forward scatter and side scatter; live cells were identified using a live/dead fixable viability stain; leukocyte populations were gated based on CD45 expression, and subsets were analyzed based on marker expression.

Results are expressed as percentages of total live cells and mean fluorescence intensity for each marker. Flow cytometry compensation was performed using single-stained control samples for each fluorochrome. Isotype controls were used to set gating for non-specific staining. All the antibodies used in flow cytometry are listed in Table S5.

All the antibodies and reagents used in cell and animal experiments can be found in Tables S1–S7.

Statistical analysis

Data analysis was performed using GraphPad Prism 9.0 software. The data presented represent the results of at least three independent experiments, each performed in triplicate ($n \geq 3$). Densitometric analysis for western blot was performed on blots from at least three independent experiments ($n \geq 3$). Distribution normality and variance homogeneity were checked before data analysis. Statistical analysis was performed using one-way ANOVA followed by the Tukey post hoc test if the data conformed to the normal distribution; otherwise, nonparametric tests were be performed. Error bars indicate the standard error of the mean. Statistical significance levels were indicated as follows: $^{*}p < 0.05$; $^{**}p < 0.01$; $^{***}p < 0.001$; $^{****}p < 0.0001$; ns, not statistically significant ($p > 0.05$).

DATA AND CODE AVAILABILITY

The data that support the findings of this study are available from the corresponding author upon reasonable request.

ACKNOWLEDGMENTS

This study was supported by STI 2030 Major Projects (2022ZD0208900) to Yi Li; STI 2030 Major Projects (2022ZD0211603), National Natural Science Foundation of China (82530100 and 82330099), the Key Area Research and Development Program of Guangdong Province (2023B0303040003) and Science and Technology Program of Guangzhou (2023A03J0708) to Yamei Tang; National Natural Science Foundation of China (82372607) to Songhua Xiao; Natural Science Foundation of Guangdong Province (2023A1515011831) to Yi Li; the Zhejiang Provincial Natural Science Foundation of China (LQN25H090017) and the Huzhou Municipal Science and Technology Bureau Project (2024GYB04) to Ying Liu.

This study was approved by the ethics committee of Sun Yat-sen University (approval no. SYSU-IACUC-2022000485) and was conducted in compliance with the Basel Declaration. Detailed data can be obtained from the corresponding author upon reasonable request.

AUTHOR CONTRIBUTIONS

Conception and design, W.L., S.X., and Y.T.; laboratory experiments, Y. Liu, Z.N., and Q.D., and Z.Z.; writing – original draft, Y. Liu, Z.N., and Q.D.; writing – review & editing, Y. Liu, Z.N., Q.D., X.Z., Z.Z., D.G., J.C., Y. Li, W.L., S.X., and Y.T.; W.L. provided the hPSC-CNC PCs; supervision, W.L., S.X., and Y.T.; funding acquisition, Y.T., Y. Liu, and Y. Li. All authors have read and approved the final version of the manuscript. The corresponding author attests that all listed authors meet authorship criteria and that no others meeting the criteria have been omitted.

DECLARATION OF INTERESTS

The authors declare no competing interests.

SUPPLEMENTAL INFORMATION

Supplemental information can be found online at https://doi.org/10.1016/j.ymthe.2025.08.024.

REFERENCES

1. Dey, A., Bhattacharya, R., Mukherjee, A., and Pandey, D.K. (2017). Natural products against Alzheimer's disease: Pharmaco-therapeutics and biotechnological interventions. Biotechnol. Adv. *35*, 178–216. https://doi.org/10.1016/j.biotechadv.2016.12.005.
2. Gauthier, S.,R.-N.P., Morais, J.A., and Webster, C. (2021). World Alzheimer Report 2021: Journey through the Diagnosis of Dementia (Alzheimer's Disease International).
3. (2018). Alzheimerś Association Report. (Alzheimer's Association).
4. Sharma, P., Srivastava, P., Seth, A., Tripathi, P.N., Banerjee, A.G., and Shrivastava, S. K. (2019). Comprehensive review of mechanisms of pathogenesis involved in Alzheimer's disease and potential therapeutic strategies. Prog. Neurobiol. *174*, 53–89. https://doi.org/10.1016/j.pneurobio.2018.12.006.
5. Söderberg, L., Johannesson, M., Nygren, P., Laudon, H., Eriksson, F., Osswald, G., Möller, C., and Lannfelt, L. (2023). Lecanemab, Aducanumab, and Gantenerumab — Binding Profiles to Different Forms of Amyloid-Beta Might Explain Efficacy and Side Effects in Clinical Trials for Alzheimer's Disease. Neurotherapeutics *20*, 195–206. https://doi.org/10.1007/s13311-022-01308-6.
6. Howard, R., McShane, R., Lindesay, J., Ritchie, C., Baldwin, A., Barber, R., Burns, A., Dening, T., Findlay, D., Holmes, C., et al. (2012). Donepezil and memantine for moderate-to-severe Alzheimer's disease. N. Engl. J. Med. *366*, 893–903. https://doi.org/10.1056/NEJMoa1106668.
7. Armulik, A., Genové, G., and Betsholtz, C. (2011). Pericytes: developmental, physiological, and pathological perspectives, problems, and promises. Dev. Cell *21*, 193–215. https://doi.org/10.1016/j.devcel.2011.07.001.
8. Sagare, A.P., Bell, R.D., Zhao, Z., Ma, Q., Winkler, E.A., Ramanathan, A., and Zlokovic, B.V. (2013). Pericyte loss influences Alzheimer-like neurodegeneration in mice. Nat. Commun. *4*, 2932. https://doi.org/10.1038/ncomms3932.
9. Sweeney, M.D., Sagare, A.P., and Zlokovic, B.V. (2018). Blood-brain barrier breakdown in Alzheimer disease and other neurodegenerative disorders. Nat. Rev. Neurol. *14*, 133–150. https://doi.org/10.1038/nrneurol.2017.188.
10. Sengillo, J.D., Winkler, E.A., Walker, C.T., Sullivan, J.S., Johnson, M., and Zlokovic, B.V. (2013). Deficiency in mural vascular cells coincides with blood-brain barrier disruption in Alzheimer's disease. Brain Pathol. *23*, 303–310. https://doi.org/10.1111/bpa.12004.
11. Armulik, A., Genové, G., Mäe, M., Nisancioglu, M.H., Wallgard, E., Niaudet, C., He, L., Norlin, J., Lindblom, P., Strittmatter, K., et al. (2010). Pericytes regulate the blood-brain barrier. Nature *468*, 557–561. https://doi.org/10.1038/nature09522.
12. Deng, P., Wang, L., Zhang, Q., Chen, S., Zhang, Y., Xu, H., Chen, H., Xu, Y., He, W., Zhang, J., and Sun, H. (2022). Therapeutic Potential of a Combination of Electroacupuncture and Human iPSC-Derived Small Extracellular Vesicles for Ischemic Stroke. Cells *11*, 820. https://doi.org/10.3390/cells11050820.

13. Sun, J., Huang, Y., Gong, J., Wang, J., Fan, Y., Cai, J., Wang, Y., Qiu, Y., Wei, Y., Xiong, C., et al. (2020). Transplantation of hPSC-derived pericyte-like cells promotes functional recovery in ischemic stroke mice. Nat. Commun. *11*, 5196. https://doi.org/10.1038/s41467-020-19042-y.

14. Wang, C., Najm, R., Xu, Q., Jeong, D.E., Walker, D., Balestra, M.E., Yoon, S.Y., Yuan, H., Li, G., Miller, Z.A., et al. (2018). Gain of toxic apolipoprotein E4 effects in human iPSC-derived neurons is ameliorated by a small-molecule structure corrector. Nat. Med. *24*, 647–657. https://doi.org/10.1038/s41591-018-0004-z.

15. Dräger, N.M., Sattler, S.M., Huang, C.T.L., Teter, O.M., Leng, K., Hashemi, S.H., Hong, J., Aviles, G., Clelland, C.D., Zhan, L., et al. (2022). A CRISPRi/a platform in human iPSC-derived microglia uncovers regulators of disease states. Nat. Neurosci. *25*, 1149–1162. https://doi.org/10.1038/s41593-022-01131-4.

16. Tachibana, M., Yamazaki, Y., Liu, C.C., Bu, G., and Kanekiyo, T. (2018). Pericyte implantation in the brain enhances cerebral blood flow and reduces amyloid-β pathology in amyloid model mice. Exp. Neurol. *300*, 13–21. https://doi.org/10.1016/j.expneurol.2017.10.023.

17. Zhang, X., Zhou, Y., Liu, Q., Wang, F., Fu, L., Wei, Y., Ye, Y., and Guo, Y. (2023). The therapeutic value of adipose-derived pericyte transplantation after intracerebral hemorrhage in rats. J. Mol. Histol. *54*, 499–508. https://doi.org/10.1007/s10735-023-10140-x.

18. Duncan, T., and Valenzuela, M. (2017). Alzheimer's disease, dementia, and stem cell therapy. Stem Cell Res. Ther. *8*, 111. https://doi.org/10.1186/s13287-017-0567-5.

19. Rodriguez, R., Rubio, R., and Menendez, P. (2012). Modeling sarcomagenesis using multipotent mesenchymal stem cells. Cell Res. *22*, 62–77. https://doi.org/10.1038/cr.2011.157.

20. Yuan, X., Wu, Q., Wang, P., Jing, Y., Yao, H., Tang, Y., Li, Z., Zhang, H., and Xiu, R. (2019). Exosomes Derived From Pericytes Improve Microcirculation and Protect Blood-Spinal Cord Barrier After Spinal Cord Injury in Mice. Front. Neurosci. *13*, 319. https://doi.org/10.3389/fnins.2019.00319.

21. Jeong, J.O., Han, J.W., Kim, J.M., Cho, H.J., Park, C., Lee, N., Kim, D.W., and Yoon, Y.S. (2011). Malignant tumor formation after transplantation of short-term cultured bone marrow mesenchymal stem cells in experimental myocardial infarction and diabetic neuropathy. Circ. Res. *108*, 1340–1347. https://doi.org/10.1161/circresaha.110.239848.

22. Katsuda, T., Kosaka, N., Takeshita, F., and Ochiya, T. (2013). The therapeutic potential of mesenchymal stem cell-derived extracellular vesicles. Proteomics *13*, 1637–1653. https://doi.org/10.1002/pmic.201200373.

23. Kim, S., Lee, S.K., Kim, H., and Kim, T.M. (2018). Exosomes Secreted from Induced Pluripotent Stem Cell-Derived Mesenchymal Stem Cells Accelerate Skin Cell Proliferation. Int. J. Mol. Sci. *19*, 3119. https://doi.org/10.3390/ijms19103119.

24. Conlan, R.S., Pisano, S., Oliveira, M.I., Ferrari, M., and Mendes Pinto, I. (2017). Exosomes as Reconfigurable Therapeutic Systems. Trends Mol. Med. *23*, 636–650. https://doi.org/10.1016/j.molmed.2017.05.003.

25. Chen, J., Tian, C., Xiong, X., Yang, Y., and Zhang, J. (2025). Extracellular vesicles: new horizons in neurodegeneration. EBioMedicine *113*, 105605. https://doi.org/10.1016/j.ebiom.2025.105605.

26. Guo, Y., Hu, D., Lian, L., Zhao, L., Li, M., Bao, H., and Xu, S. (2023). Stem Cell-derived Extracellular Vesicles: A Promising Nano Delivery Platform to the Brain? Stem Cell Rev. Rep. *19*, 285–308. https://doi.org/10.1007/s12015-022-10455-4.

27. Wu, H., Zhou, X., Wang, X., Cheng, W., Hu, X., Wang, Y., Luo, B., Huang, W., and Gu, J. (2021). miR-34a in extracellular vesicles from bone marrow mesenchymal stem cells reduces rheumatoid arthritis inflammation via the cyclin I/ATM/ATR/p53 axis. J. Cell. Mol. Med. *25*, 1896–1910. https://doi.org/10.1111/jcmm.15857.

28. Nojehdehi, S., Soudi, S., Hesampour, A., Rasouli, S., Soleimani, M., and Hashemi, S. M. (2018). Immunomodulatory effects of mesenchymal stem cell-derived exosomes on experimental type-1 autoimmune diabetes. J. Cell. Biochem. *119*, 9433–9443. https://doi.org/10.1002/jcb.27260.

29. Ramirez-Gomez, J., Dalal, S., Devara, D., Sharma, B., Rodarte, D., and Kumar, S. (2025). MicroRNA-based recent research developments in Alzheimer's disease. J. Alzheimers Dis. *104*, 14–31. https://doi.org/10.1177/13872877241313397.

30. Lukiw, W.J. (2023). MicroRNA (miRNA) Complexity in Alzheimer's Disease (AD). Biology (Basel) *12*, 788. https://doi.org/10.3390/biology12060788.

31. Ouyang, Q., Liu, K., Zhu, Q., Deng, H., Le, Y., Ouyang, W., Yan, X., Zhou, W., and Tong, J. (2022). Brain-Penetration and Neuron-Targeting DNA Nanoflowers Co-Delivering miR-124 and Rutin for Synergistic Therapy of Alzheimer's Disease. Small *18*, e2107534. https://doi.org/10.1002/smll.202107534.

32. Sharma, K., Zhang, Y., Paudel, K.R., Kachelmeier, A., Hansbro, P.M., and Shi, X. (2022). The Emerging Role of Pericyte-Derived Extracellular Vesicles in Vascular and Neurological Health. Cells *11*, 3108. https://doi.org/10.3390/cells11193108.

33. Shaw, I., Rider, S., Mullins, J., Hughes, J., and Péault, B. (2018). Pericytes in the renal vasculature: roles in health and disease. Nat. Rev. Nephrol. *14*, 521–534. https://doi.org/10.1038/s41581-018-0032-4.

34. Zheng, Z., Chopp, M., and Chen, J. (2020). Multifaceted roles of pericytes in central nervous system homeostasis and disease. J. Cereb. Blood Flow Metab. *40*, 1381–1401. https://doi.org/10.1177/0271678x20911331.

35. Cheng, J., Korte, N., Nortley, R., Sethi, H., Tang, Y., and Attwell, D. (2018). Targeting pericytes for therapeutic approaches to neurological disorders. Acta Neuropathol. *136*, 507–523. https://doi.org/10.1007/s00401-018-1893-0.

36. Haney, M.J., Zhao, Y., Fay, J., Duhyeong, H., Wang, M., Wang, H., Li, Z., Lee, Y.Z., Karuppan, M.K., El-Hage, N., et al. (2020). Genetically modified macrophages accomplish targeted gene delivery to the inflamed brain in transgenic Parkin Q311X(A) mice: importance of administration routes. Sci. Rep. *10*, 11818. https://doi.org/10.1038/s41598-020-68874-7.

37. Li, J., Zheng, M., Shimoni, O., Banks, W.A., Bush, A.I., Gamble, J.R., and Shi, B. (2021). Development of Novel Therapeutics Targeting the Blood-Brain Barrier: From Barrier to Carrier. Adv. Sci. *8*, e2101090. https://doi.org/10.1002/advs.202101090.

38. Uddin, M.S., and Lim, L.W. (2022). Glial cells in Alzheimer's disease: From neuropathological changes to therapeutic implications. Ageing Res. Rev. *78*, 101622. https://doi.org/10.1016/j.arr.2022.101622.

39. Tariot, P.N. (2003). Medical management of advanced dementia. J. Am. Geriatr. Soc. *51*, S305–S313. https://doi.org/10.1046/j.1532-5415.5156.x.

40. Schneider, L.S., Mangialasche, F., Andreasen, N., Feldman, H., Giacobini, E., Jones, R., Mantua, V., Mecocci, P., Pani, L., Winblad, B., and Kivipelto, M. (2014). Clinical trials and late-stage drug development for Alzheimer's disease: an appraisal from 1984 to 2014. J. Intern. Med. *275*, 251–283. https://doi.org/10.1111/joim.12191.

41. Boxer, A.L., and Sperling, R. (2023). Accelerating Alzheimer's therapeutic development: The past and future of clinical trials. Cell *186*, 4757–4772. https://doi.org/10.1016/j.cell.2023.09.023.

42. Tolar, M., Hey, J., Power, A., and Abushakra, S. (2021). Neurotoxic Soluble Amyloid Oligomers Drive Alzheimer's Pathogenesis and Represent a Clinically Validated Target for Slowing Disease Progression. Int. J. Mol. Sci. *22*, 6355. https://doi.org/10.3390/ijms22126355.

43. Tolar, M., Abushakra, S., and Sabbagh, M. (2020). The path forward in Alzheimer's disease therapeutics: Reevaluating the amyloid cascade hypothesis. Alzheimers Dement. *16*, 1553–1560. https://doi.org/10.1016/j.jalz.2019.09.075.

44. van Dyck, C.H., Swanson, C.J., Aisen, P., Bateman, R.J., Chen, C., Gee, M., Kanekiyo, M., Li, D., Reyderman, L., Cohen, S., et al. (2023). Lecanemab in Early Alzheimer's Disease. N. Engl. J. Med. *388*, 9–21. https://doi.org/10.1056/NEJMoa2212948.

45. Lee, R.H., Pulin, A.A., Seo, M.J., Kota, D.J., Ylostalo, J., Larson, B.L., Semprun-Prieto, L., Delafontaine, P., and Prockop, D.J. (2009). Intravenous hMSCs improve myocardial infarction in mice because cells embolized in lung are activated to secrete the anti-inflammatory protein TSG-6. Cell Stem Cell *5*, 54–63. https://doi.org/10.1016/j.stem.2009.05.003.

46. Colombo, M., Raposo, G., and Théry, C. (2014). Biogenesis, secretion, and intercellular interactions of exosomes and other extracellular vesicles. Annu. Rev. Cell Dev. Biol. *30*, 255–289. https://doi.org/10.1146/annurev-cellbio-101512-122326.

47. Hall, J., Prabhakar, S., Balaj, L., Lai, C.P., Cerione, R.A., and Breakefield, X.O. (2016). Delivery of Therapeutic Proteins via Extracellular Vesicles: Review and Potential Treatments for Parkinson's Disease, Glioma, and Schwannoma. Cell. Mol. Neurobiol. *36*, 417–427. https://doi.org/10.1007/s10571-015-0309-0.

48. Chen, X., Wang, S., and Cao, W. (2018). Mesenchymal stem cell-mediated immunomodulation in cell therapy of neurodegenerative diseases. Cell. Immunol. *326*, 8–14. https://doi.org/10.1016/j.cellimm.2017.06.006.

49. Wei, H., Xu, Y., Chen, Q., Chen, H., Zhu, X., and Li, Y. (2020). Mesenchymal stem cell-derived exosomal miR-223 regulates neuronal cell apoptosis. Cell Death Dis. *11*, 290. https://doi.org/10.1038/s41419-020-2490-4.

50. Lee, M., Ban, J.J., Yang, S., Im, W., and Kim, M. (2018). The exosome of adipose-derived stem cells reduces β-amyloid pathology and apoptosis of neuronal cells derived from the transgenic mouse model of Alzheimer's disease. Brain Res. *1691*, 87–93. https://doi.org/10.1016/j.brainres.2018.03.034.

51. Wang, S.S., Jia, J., and Wang, Z. (2018). Mesenchymal Stem Cell-Derived Extracellular Vesicles Suppresses iNOS Expression and Ameliorates Neural Impairment in Alzheimer's Disease Mice. J. Alzheimers Dis. *61*, 1005–1013. https://doi.org/10.3233/jad-170848.

52. Garcia-Contreras, M., and Thakor, A.S. (2021). Human adipose tissue-derived mesenchymal stem cells and their extracellular vesicles modulate lipopolysaccharide activated human microglia. Cell Death Discov. *7*, 98. https://doi.org/10.1038/s41420-021-00471-7.

53. Gaceb, A., Özen, I., Padel, T., Barbariga, M., and Paul, G. (2018). Pericytes secrete pro-regenerative molecules in response to platelet-derived growth factor-BB. J. Cereb. Blood Flow Metab. *38*, 45–57. https://doi.org/10.1177/0271678x17719645.

54. Wu, Q., Yuan, X., Li, B., Yang, J., Han, R., Zhang, H., and Xiu, R. (2020). Differential miRNA expression analysis of extracellular vesicles from brain microvascular pericytes in spontaneous hypertensive rats. Biotechnol. Lett. *42*, 389–401. https://doi.org/10.1007/s10529-019-02788-x.

55. Yin, G.N., Park, S.H., Ock, J., Choi, M.J., Limanjaya, A., Ghatak, K., Song, K.M., Kwon, M.H., Kim, D.K., Gho, Y.S., et al. (2020). Pericyte-Derived Extracellular Vesicle-Mimetic Nanovesicles Restore Erectile Function by Enhancing Neurovascular Regeneration in a Mouse Model of Cavernous Nerve Injury. J. Sex. Med. *17*, 2118–2128. https://doi.org/10.1016/j.jsxm.2020.07.083.

56. Yin, G.N., Shin, T.Y., Ock, J., Choi, M.J., Limanjaya, A., Kwon, M.H., Liu, F.Y., Hong, S.S., Kang, J.H., Gho, Y.S., et al. (2022). Pericyte-derived extracellular vesicles-mimetic nanovesicles improves peripheral nerve regeneration in mouse models of sciatic nerve transection. Int. J. Mol. Med. *49*, 18. https://doi.org/10.3892/ijmm.2021.5073.

57. Douvris, A., Viñas, J., and Burns, K.D. (2022). miRNA-486-5p: signaling targets and role in non-malignant disease. Cell. Mol. Life Sci. *79*, 376. https://doi.org/10.1007/s00018-022-04406-y.

58. Liu, L., Xiao, S., Wang, Y., Zhu, Z., Cao, Y., Yang, S., Mai, R., and Zheng, Y. (2022). Identification of a novel circular RNA circZNF652/miR-486-5p/SERPINE1 signaling cascade that regulates cancer aggressiveness in glioblastoma (GBM). Bioengineered *13*, 1411–1423. https://doi.org/10.1080/21655979.2021.2018096.

59. Kurz, A., Kumar, R., Northoff, B.H., Wenk, C., Schirra, J., Donakonda, S., Höglinger, G.U., Schwarz, J., Rozanski, V., Hübner, R., et al. (2021). Differential expression of gut miRNAs in idiopathic Parkinson's disease. Parkinsonism Relat. Disord. *88*, 46–50. https://doi.org/10.1016/j.parkreldis.2021.05.022.

60. Dori, M., Cavalli, D., Lesche, M., Massalini, S., Alieh, L.H.A., de Toledo, B.C., Khudayberdiev, S., Schratt, G., Dahl, A., and Calegari, F. (2020). MicroRNA profiling of mouse cortical progenitors and neurons reveals miR-486-5p as a regulator of neurogenesis. Development *147*, dev190520. https://doi.org/10.1242/dev.190520.

61. Kaur, N., and Aran, K.R. (2025). Uncovering the intricacies of IGF-1 in Alzheimer's disease: new insights from regulation to therapeutic targeting. Inflammopharmacology *33*, 1311–1330. https://doi.org/10.1007/s10787-025-01641-0.

62. Miao, J., Zhang, Y., Su, C., Zheng, Q., and Guo, J. (2025). Insulin-Like Growth Factor Signaling in Alzheimer's Disease: Pathophysiology and Therapeutic Strategies. Mol. Neurobiol. *62*, 3195–3225. https://doi.org/10.1007/s12035-024-04457-1.

63. Holzenberger, M., Dupont, J., Ducos, B., Leneuve, P., Géloën, A., Even, P.C., Cervera, P., and Le Bouc, Y. (2003). IGF-1 receptor regulates lifespan and resistance to oxidative stress in mice. Nature *421*, 182–187. https://doi.org/10.1038/nature01298.

64. Taguchi, A., Wartschow, L.M., and White, M.F. (2007). Brain IRS2 signaling coordinates life span and nutrient homeostasis. Science *317*, 369–372. https://doi.org/10.1126/science.1142179.

65. Cohen, E., Paulsson, J.F., Blinder, P., Burstyn-Cohen, T., Du, D., Estepa, G., Adame, A., Pham, H.M., Holzenberger, M., Kelly, J.W., et al. (2009). Reduced IGF-1 signaling delays age-associated proteotoxicity in mice. Cell *139*, 1157–1169. https://doi.org/10.1016/j.cell.2009.11.014.

66. Gontier, G., George, C., Chaker, Z., Holzenberger, M., and Aïd, S. (2015). Blocking IGF Signaling in Adult Neurons Alleviates Alzheimer's Disease Pathology through Amyloid-β Clearance. J. Neurosci. *35*, 11500–11513. https://doi.org/10.1523/jneurosci.0343-15.2015.

67. George, C., Gontier, G., Lacube, P., François, J.C., Holzenberger, M., and Aïd, S. (2017). The Alzheimer's disease transcriptome mimics the neuroprotective signature of IGF-1 receptor-deficient neurons. Brain *140*, 2012–2027. https://doi.org/10.1093/brain/awx132.

68. El-Ami, T., Moll, L., Carvalhal Marques, F., Volovik, Y., Reuveni, H., and Cohen, E. (2014). A novel inhibitor of the insulin/IGF signaling pathway protects from age-onset, neurodegeneration-linked proteotoxicity. Aging Cell *13*, 165–174. https://doi.org/10.1111/acel.12171.

69. Sohrabi, M., Floden, A.M., Manocha, G.D., Klug, M.G., and Combs, C.K. (2020). IGF-1R Inhibitor Ameliorates Neuroinflammation in an Alzheimer's Disease Transgenic Mouse Model. Front. Cell. Neurosci. *14*, 200. https://doi.org/10.3389/fncel.2020.00200.

70. Lim, L.P., Lau, N.C., Garrett-Engele, P., Grimson, A., Schelter, J.M., Castle, J., Bartel, D.P., Linsley, P.S., and Johnson, J.M. (2005). Microarray analysis shows that some microRNAs downregulate large numbers of target mRNAs. Nature *433*, 769–773. https://doi.org/10.1038/nature03315.

71. Selbach, M., Schwanhäusser, B., Thierfelder, N., Fang, Z., Khanin, R., and Rajewsky, N. (2008). Widespread changes in protein synthesis induced by microRNAs. Nature *455*, 58–63. https://doi.org/10.1038/nature07228.

72. Bartel, D.P. (2018). Metazoan MicroRNAs. Cell *173*, 20–51. https://doi.org/10.1016/j.cell.2018.03.006.

73. Pinzón, N., Li, B., Martinez, L., Sergeeva, A., Presumey, J., Apparailly, F., and Seitz, H. (2017). microRNA target prediction programs predict many false positives. Genome Res. *27*, 234–245. https://doi.org/10.1101/gr.205146.116.

74. Miller, N.A., Reddy, M.B., Heikkinen, A.T., Lukacova, V., and Parrott, N. (2019). Physiologically Based Pharmacokinetic Modelling for First-In-Human Predictions: An Updated Model Building Strategy Illustrated with Challenging Industry Case Studies. Clin. Pharmacokinet. *58*, 727–746. https://doi.org/10.1007/s40262-019-00741-9.

75. Oakley, H., Cole, S.L., Logan, S., Maus, E., Shao, P., Craft, J., Guillozet-Bongaarts, A., Ohno, M., Disterhoft, J., Van Eldik, L., et al. (2006). Intraneuronal beta-amyloid aggregates, neurodegeneration, and neuron loss in transgenic mice with five familial Alzheimer's disease mutations: potential factors in amyloid plaque formation. J. Neurosci. *26*, 10129–10140. https://doi.org/10.1523/jneurosci.1202-06.2006.

76. Ke, Q., Li, L., Cai, B., Liu, C., Yang, Y., Gao, Y., Huang, W., Yuan, X., Wang, T., Zhang, Q., et al. (2013). Connexin 43 is involved in the generation of human-induced pluripotent stem cells. Hum. Mol. Genet. *22*, 2221–2233. https://doi.org/10.1093/hmg/ddt074.

77. Jiaqi Sun, Y.H., Gong, J., Wei, Y., Xiong, C., Xiang, A.P., and Li, W. (2020). Differentiation protocol for cranial neural crest-derived pericyte-like cells from human pluripotent stem cells. Protoc. Exch. *1*, e21. https://doi.org/10.21203/rs.3.pex-1116/v1.

78. Kwon, J., Kim, M.Y., Lee, J.I., Kim, W., Hyun, J.E., and Yoon, H.Y. (2024). Medication effects on pulmonary thromboembolism in mice intravenously transplanted with canine adipose tissue-derived mesenchymal stem cells. J. Vet. Sci. *25*, e80. https://doi.org/10.4142/jvs.24163.

79. Modo, M., Rezaie, P., Heuschling, P., Patel, S., Male, D.K., and Hodges, H. (2002). Transplantation of neural stem cells in a rat model of stroke: assessment of short-term graft survival and acute host immunological response. Brain Res. *958*, 70–82. https://doi.org/10.1016/s0006-8993(02)03463-7.

80. Naaldijk, Y., Jäger, C., Fabian, C., Leovsky, C., Blüher, A., Rudolph, L., Hinze, A., and Stolzing, A. (2017). Effect of systemic transplantation of bone marrow-derived mesenchymal stem cells on neuropathology markers in APP/PS1 Alzheimer mice. Neuropathol. Appl. Neurobiol. *43*, 299–314. https://doi.org/10.1111/nan.12319.

81. Neves, A.F., Camargo, C., Premer, C., Hare, J.M., Baumel, B.S., and Pinto, M. (2021). Intravenous administration of mesenchymal stem cells reduces Tau

phosphorylation and inflammation in the 3xTg-AD mouse model of Alzheimer's disease. Exp. Neurol. *341*, 113706. https://doi.org/10.1016/j.expneurol.2021.113706.

82. Lobb, R.J., Becker, M., Wen, S.W., Wong, C.S.F., Wiegmans, A.P., Leimgruber, A., and Möller, A. (2015). Optimized exosome isolation protocol for cell culture supernatant and human plasma. J. Extracell. Vesicles *4*, 27031. https://doi.org/10.3402/jev.v4.27031.

83. Yang, B.-l., Long, Y.-y., Lei, Q., Gao, F., Ren, W.-x., Cao, Y.-l., Wu, D., Xu, L.-y., Qu, J., Li, H., et al. (2024). Lethal pulmonary thromboembolism in mice induced by intravenous human umbilical cord mesenchymal stem cell-derived large extracellular vesicles in a dose- and tissue factor-dependent manner. Acta Pharmacol. Sin. *45*, 2300–2312. https://doi.org/10.1038/s41401-024-01327-3.

84. Hanson, L.R., Fine, J.M., Svitak, A.L., and Faltesek, K.A. (2013). Intranasal administration of CNS therapeutics to awake mice. J. Vis. Exp. *74*, 4440. https://doi.org/10.3791/4440.

85. Stine, W.B., Jr., Dahlgren, K.N., Krafft, G.A., and LaDu, M.J. (2003). In vitro characterization of conditions for amyloid-beta peptide oligomerization and fibrillogenesis. J. Biol. Chem. *278*, 11612–11622. https://doi.org/10.1074/jbc.M210207200.

86. Leger, M., Quiedeville, A., Bouet, V., Haelewyn, B., Boulouard, M., Schumann-Bard, P., and Freret, T. (2013). Object recognition test in mice. Nat. Protoc. *8*, 2531–2537. https://doi.org/10.1038/nprot.2013.155.

87. Mathiesen Janiurek, M., Soylu-Kucharz, R., Christoffersen, C., Kucharz, K., and Lauritzen, M. (2019). Apolipoprotein M-bound sphingosine-1-phosphate regulates blood-brain barrier paracellular permeability and transcytosis. Elife *8*, e49405. https://doi.org/10.7554/eLife.49405.

88. Ayloo, S., Lazo, C.G., Sun, S., Zhang, W., Cui, B., and Gu, C. (2022). Pericyte-to-endothelial cell signaling via vitronectin-integrin regulates blood-CNS barrier. Neuron *110*, 1641–1655.e6. https://doi.org/10.1016/j.neuron.2022.02.017.

YMTHE, Volume *33*

Supplemental Information

Intravenously injected hPSC-derived pericytes for Alzheimer disease: Neuroprotection and vascular repair via extracellular vesicles

Ying Liu, Zhiyuan Ning, Qingyuan Dai, Xinkai Zhang, Yibin Xiao, Zhan Zhang, Daji Guo, Junhua Chen, Yi Li, Weiqiang Li, Songhua Xiao, and Yamei Tang

Table S1. Top 20 miRNA in the EVs

ID	miRNA	Average_UMI
MIMAT0002177	hsa-miR-486-5p	100378.6667
MIMAT0016887	hsa-miR-4325	64528.66667
MIMAT0026613	hsa-miR-510-3p	9010
MIMAT0027477	hsa-miR-6788-3p	7454.666667
MIMAT0000423	hsa-miR-125b-5p	5873.666667
MIMAT0022947	hsa-miR-1238-5p	5082
MIMAT0000062	hsa-let-7a-5p	4658.333333
MIMAT0031014	hsa-miR-8087	4114.333333
MIMAT0003278	hsa-miR-610	3103
MIMAT0000076	hsa-miR-21-5p	2316.333333
MIMAT0027494	hsa-miR-6797-5p	2192.666667
MIMAT0012735	hsa-miR-718	2152
MIMAT0000232	hsa-miR-199a-3p	2081.333333
MIMAT0004563	hsa-miR-199b-3p	2069.333333
MIMAT0000069	hsa-miR-16-5p	1978
MIMAT0000261	hsa-miR-183-5p	1932.666667
MIMAT0027647	hsa-miR-6873-3p	1837
MIMAT0027507	hsa-miR-6803-3p	1770.333333
MIMAT0015062	hsa-miR-3182	1684.666667
MIMAT0027384	hsa-miR-6741-3p	1564.333333

Table S2. Primers used for quantitative PCR

Gene	Direction	Sequences
U6-F	5'-3'	CTCGCTTCGGCAGCACA
U6-R	5'-3'	ACGCTTCACGAATTTGC
miR-486-5p-F	5'-3'	TCCTGTACTGAGCTGCCCCGAG
Igf1r-F	5'-3'	GTGGGGGCTCGTGTTTCTC
Igf1r-R	5'-3'	GATCACCGTGCAGTTTTCCA
PDGFRβ-F	5'-3'	AATGCTGAGCGACCACTCCATC
PDGFRβ-R	5'-3'	TCGGGTCATGTTCAAGTCCAGC
NG2-R	5'-3'	GAGGTCTTGGTGAACTTCACCC
NG2-F	5'-3'	GACAGTAGGAGACCGATGGTGT
Human ALU-F	5'-3'	CAT GGT GAA ACC CCG TCT CTA
Human ALU-R	5'-3'	GCC TCA GCC TCC CGA GTA G
Mouse Gapdh-F	5'-3'	CAT CAC TGC CAC CCA GAA GAC TG
Mouse Gapdh-R	5'-3'	ATG CCA GTG AGC TTC CCG TTC AG
Mmp19-F	5'-3'	AGG CAC TCA TGG CTC CTG TCT A
Mmp19-R	5'-3'	TGA GCA TCT CGG TCT CTT CCT C
Smo-F	5'-3'	GAG GCT ACT TCC TCA TCA GAG G
Smo-R	5'-3'	GCT GAA GGT GAT GAG CAC AAA GC
Gli1-F	5'-3'	CTC AAA CTG CCC AGC TTA ACC C
Gli1-R	5'-3'	TGC GGC TGA CTG TGT AAG CAG A
Dock3-F	5'-3'	CTT CTC CAT CGT CAA GAC CAG C
Dock3-R	5'-3'	GAT GGT GAG GAG AGT CTG TAG G
Foxo1-F	5'-3'	CTA CGA GTG GAT GGT GAA GAG C
Foxo1-R	5'-3'	CCA GTT CCT TCA TTC TGC ACT CG
Smad1-F	5'-3'	CTG AAG CCT CTG GAA TGC TGT G
Smad1-R	5'-3'	CAG AAG GCT GTG CTG AGG ATT G
Smad4-F	5'-3'	CAG CCA TAG TGA AGG ACT GTT GC
Smad4-R	5'-3'	CCT ACT TCC AGT CCA GGT GGT A
Tbx2-F	5'-3'	TCA TCG CTG TCA CTG CCT ACC A
Tbx2-R	5'-3'	CGG CTT ACA GTG CTC CTC ATA C

Table S3. Antibodies used in Western blotting

Antigen	Host	Dilution	Company	Cat. No
PSD95	rabbit	1:2000	Abcam	ab18258
α-Actinin	mouse	1:1000	Proteintech	66895-1-IG
β-Tubulin	mouse	1:5000	Proteintech	66240-1-Ig
ZO-1	rabbit	1:1000	Invitrogen	61-7300
SYP	rabbit	1:2000	Abcam	ab32127

Table S4. Antibodies and dyes used in immunofluorescence staining.

**Antigen**	**Host**	**Dilution**	**Company**	**Cat. No**
ZO-1	rabbit	1:1000	Invitrogen	61-7300
PDGFRβ	rabbit	1:1000	Cell Signaling Technology	4564S
CD31	rabbit	1:1000	Abcam	ab28364
CD13	rat	1:200	Abcam	ab33489
Iba1	Rabbit	1:1000	Wako	019-19741
GFAP	mouse	1:1000	Servicebio	GB12096
βIII-tubulin	rabbit	1：500	Beyotime	AF1216
CD9	rabbit	1：2000	Abmart	T55337M
CD63	rabbit	1：2000	Abmart	T55253S
TSG101	rabbit	1：2000	Abmart	T55985M
Fibrinogen	rabbit	1：500	Solarbio	K114703P
DAPI	/	1:1000	Cell Signaling Technology	4083S
PKH26	/	/	Sigma-Aldrich	MINI26
EVlink 505	/	/	TINGOscience	EL012100200
anti-mouse IgG HRP-linked Ab	horse	1:1000	Cell Signaling Technology	7076S
anti-rabbit IgG HRP-linked Ab	horse	1:1000	Cell Signaling Technology	7074S

Table S5. Reagents used for flow cytometry:

Reagent	Catalog No.	Clone	Brand
Zombie NIR™ Fixable Viability Kit	423105	–	BioLegend
TruStain FcX™ (anti-mouse CD16/32)	101319	93	BioLegend
APC anti-mouse F4/80	123116	BM8	BioLegend
FITC anti-mouse CD45	157214	S18009F	BioLegend
APC/Cyanine7 anti-mouse CD3	100221	17A2	BioLegend
BV786 Rat anti-mouse CD4	563331	GK1.5	BioLegend
Brilliant Violet 605™ anti-mouse CD8a	100744	53-6.7	BioLegend
PE anti-mouse CD49b (pan-NK cells)	108907	–	BioLegend
Alexa Fluor® 700 anti-mouse/human CD11b	101222	M1/70	BioLegend
BV421 Rat anti-mouse Ly-6C	562727	AL-21	BD Horizon™
PE/Cyanine5 anti-mouse Ly-6G	127671	–	BioLegend
Brilliant Stain Buffer	563794	–	BD Horizon™

Table S6. Fluorescent dyes for in vivo two-photon imaging

**Reagent**	**MW (kDa)**	**Company**	**Cat. No.**	**Dilution**
Fluorescein isothiocyanate–dextran	4	Sigma-Aldrich	46944	10mg/ml in saline
Dextran, Texas Red	70	Invitrogen	D1830	10mg/ml in saline

Table S7. Other major reagents used in cell and animal experiments

Reagent	Company	Cat. No
Human Pericyte Medium (PM)	ScienCell Research Laboratories	1201
pericyte growth additive for PM	ScienCell Research Laboratories	1252
penicillin/streptomycin solution for PM	ScienCell Research Laboratories	0503
fetal bovine serum for PM	ScienCell Research Laboratories	0010
Dulbecco's modified Eagle's medium (DMEM)	Gibco	C11965500BT
phosphate-buffered saline solution (PBS)	Servicebio	G4202
Cyclosporine A (CSA)	Solarbio	SC5120
BCA assay	Beyotime	P0012S
0.3% Triton X-100	Sigma-Aldrich	X100
BSA	Sigma-Aldrich	A1933
Aβ1-42 lyophilized powder	GenScript	RP10017CN
Hexafluoroisopropanol (HFIP)	Macklin	H811027
dimethyl sulfoxide (DMSO)	Sigma-Aldrich	D2650
miRNA mimics /inhibitors	RiboBio	miR10002177-1
MicrON hsa-miR-486-5p agomir and negative control	RiboBio	miR40002177-4-5
1M Tris-HCl, pH 8.8	Beyotime	ST788
1M Tris-HCl, pH 6.8	Beyotime	ST768
RIPA lysis buffer	Beyotime	P0013B
miRNA 1st Strand cDNA Synthesis Kit (by tailing A)	Vazyme	MR201
Taq Pro Universal SYBR qPCR Master Mix	Vazyme	Q712
PrimeScript™ RT Master Mix (Perfect Real Time)	Shiga	RR036A
SYBR qPCR SuperMix Plus kit	Novoprotein	E096-01B
Nissol stain assay	Solarbio	DK0022100

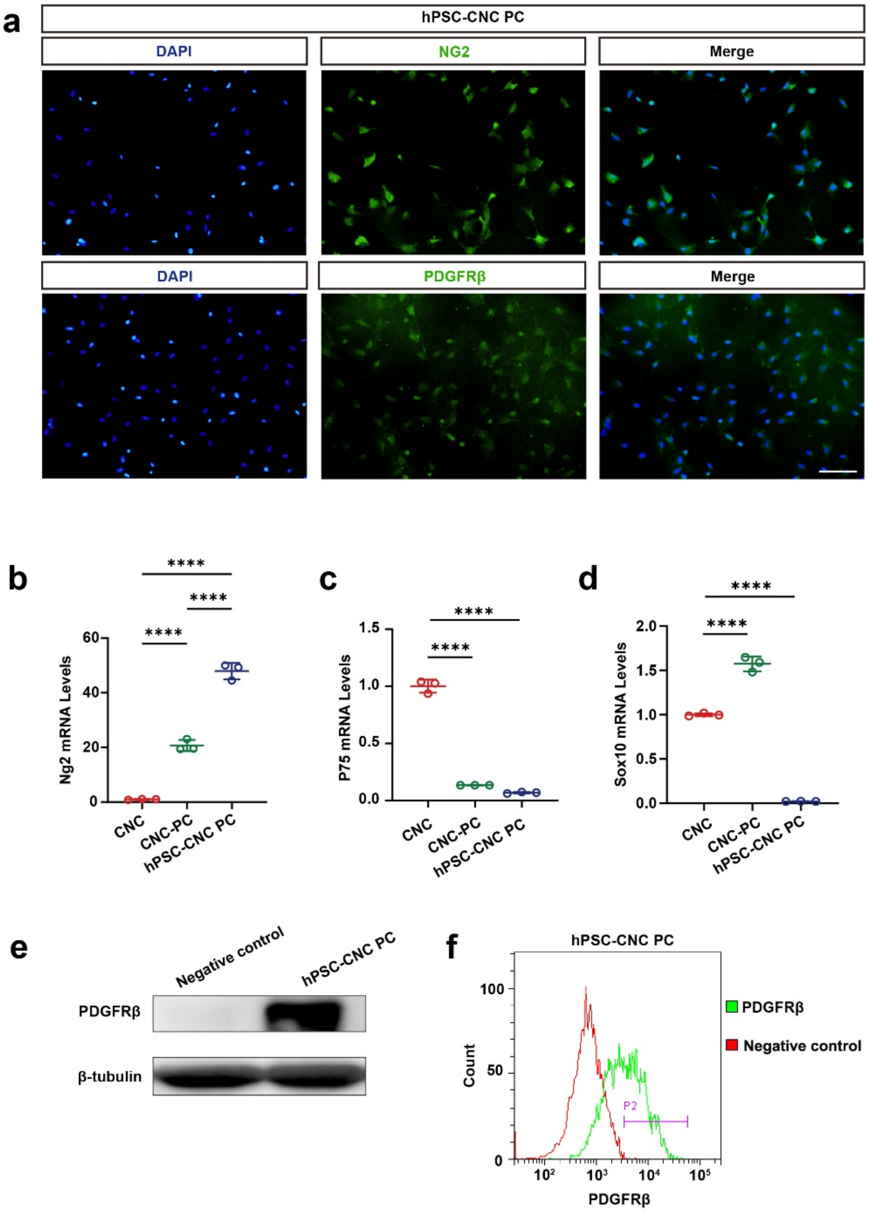

Figure S1. Characteristics of human pluripotent stem cell-derived pericyte-like cells (hPSC-PCs).

(a) Immunofluorescence staining indicated that the induced cells expressed the pericyte markers NG2 and PDGFRβ. (b–d) RT-PCR analysis demonstrated high *NG2* expression and low expression of the neural crest markers *P75* and *SOX10*. (e–f) Western blotting and flow cytometry further confirmed the high expression of the pericyte marker PDGFRβ in these cells. PDGFRβ-negative BV2 cells were used as a negative control.

(b, c, d) Data are presented as the mean ± S.E. The experiment had no less than three independent biological replicates (One-way ANOVA). ****, P<0.0001.

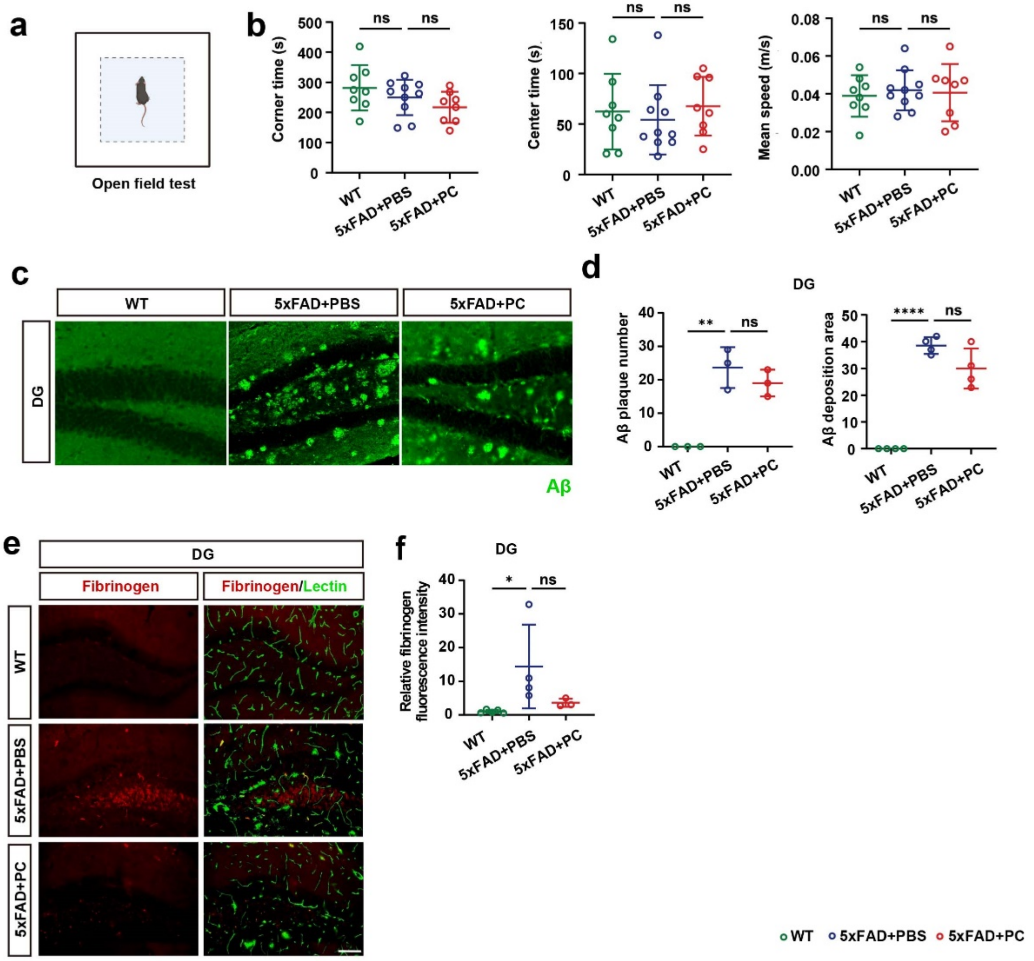

Figure S2. The hPSC-CNC PC treatment had no impact on mouse locomotion, and no differences of Aβ deposition and BBB leakage were found in DG area after cell injection.

(a) A diagram of open field test. (b) The hPSC-CNC PC treatment had no impact on the locomotion of the mice according to open field test. (c) Representative fluorescence photomicrographs of Aβ in the hippocampal DG area of WT, 5XFAD, and 5XFAD mice with hPSC-CNC PCs transplantation. (c-d) Fluorescence intensity and plaque number of Aβ in the hippocampal CA1 region were quantified in each visible field. No differences were found among three groups in the fluorescence area and plaque number of Aβ in DG. (e) Representative fluorescence photomicrographs of fibrinogen in the hippocampal DG area of WT, 5XFAD, and 5XFAD mice with hPSC-CNC PCs transplantation. (f) Immunofluorescence staining of fibrinogen revealed no significant differences of BBB leakage among three groups in the hippocampal DG region.

(b, d, f) Data are presented as the mean ± S.E. The experiment had no less than three independent biological replicates (One-way ANOVA). ns, P≥0.05; *, P<0.05; **, P<0.01; ****, P<0.0001.

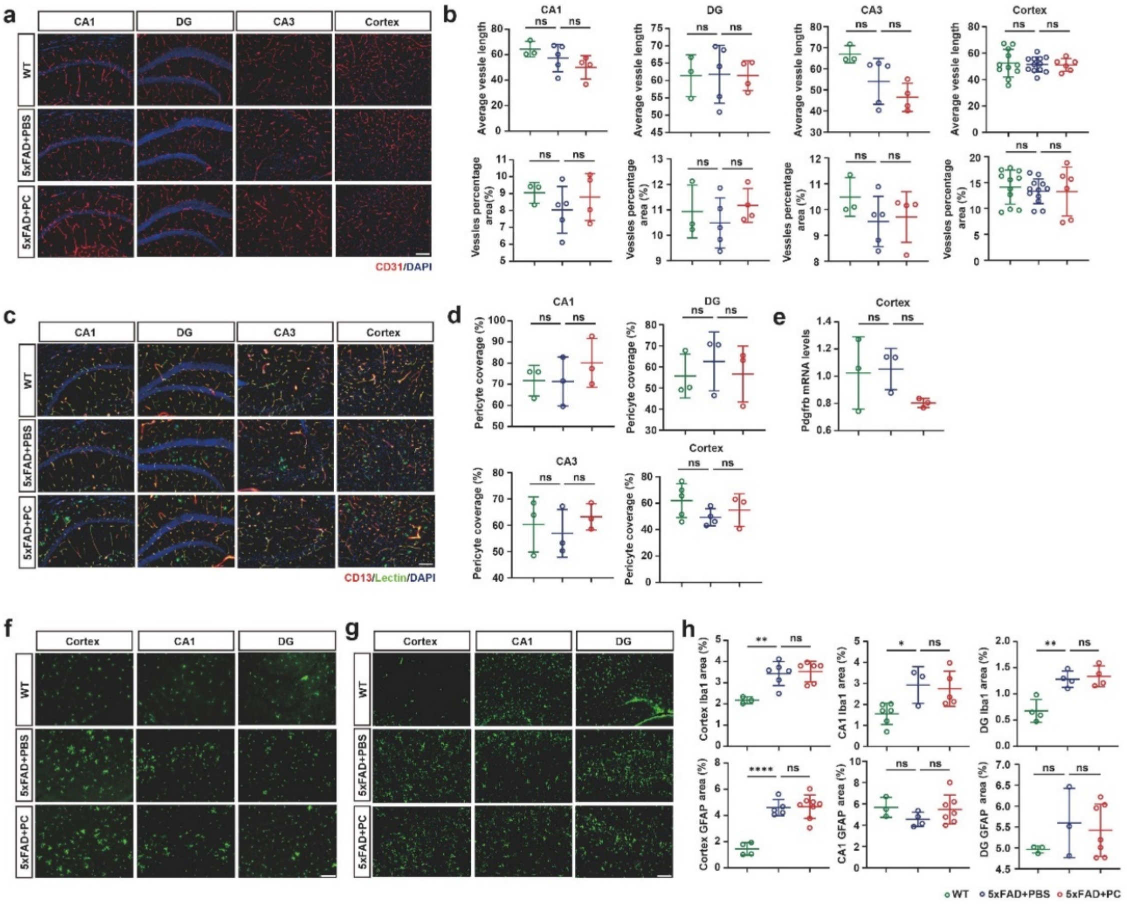

Figure S3. Other types of cells in the central nervous system did not change significantly after hPSC-CNC PC transplantation.

(a–b) Blood vessel length remained unchanged after hPSC-PCs injection. (c–e) Immunofluorescence and RT-PCR results showed no significant changes in the coverage area of pericytes or in the expression of the marker *Pdgfrb* RNA. (e) RT-PCR also indicated that while the RNA expression of inflammatory factors in the nervous system exhibited a downward trend post-cell injection, it did not reach statistical significance. (f-h) Immunofluorescence showed no significant changes in the fluorescence area of IBA1+ microglia or GFAP+ astrocyte after hPSC-PCs injection.

(b, d, e, h) Data are presented as the mean ± S.E. The experiment had no less than three independent biological replicates (One-way ANOVA). ns, P≥0.05; *, P<0.05; **, P<0.01; ****, P<0.0001.

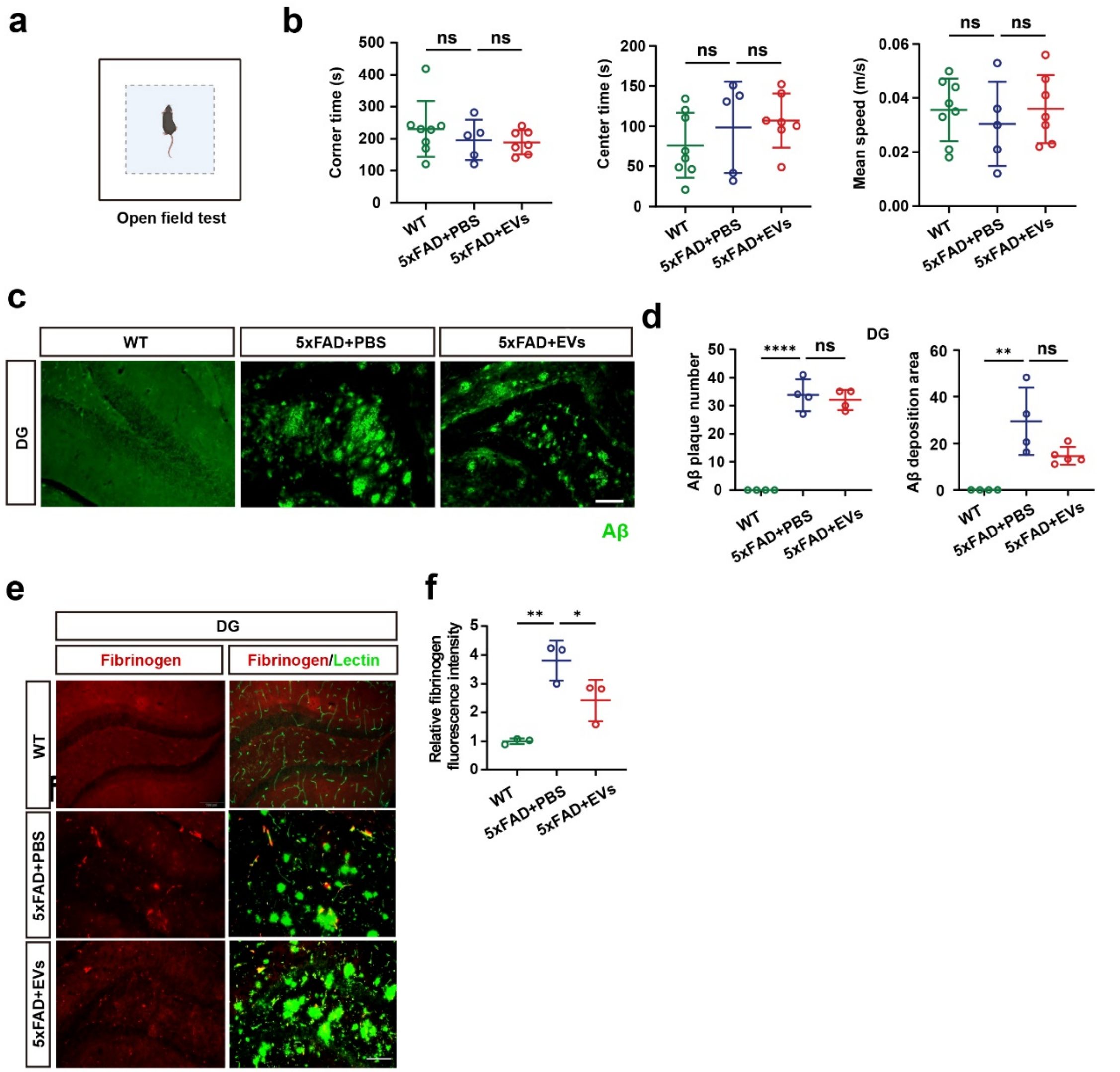

Figure S4. EV treatment had no impact on mouse locomotion or Aβ deposition in DG area, but decreased the BBB leakage in DG area.

(a) A diagram of open field test. (b) The EV treatment had no impact on the locomotion of the mice according to open field test. (c) Representative fluorescence photomicrographs of Aβ in the hippocampal DG area of WT, 5XFAD, and 5XFAD mice with EV transplantation. (d) No differences were found among three groups in the fluorescence intensity and plaque number of Aβ in DG. (e) Representative fluorescence photomicrographs of fibrinogen in the hippocampal DG area of WT, 5XFAD, and 5XFAD mice with EV transplantation. (f) Immunofluorescence staining of fibrinogen revealed significant decrease of BBB leakage in 5xFAD mice after EV injection in the hippocampal DG region.

(b, d, f) Data are presented as the mean ± S.E. The experiment had no less than three independent biological replicates (One-way ANOVA). ns, P≥0.05; *, P<0.05; **, P<0.01; ****, P<0.0001.

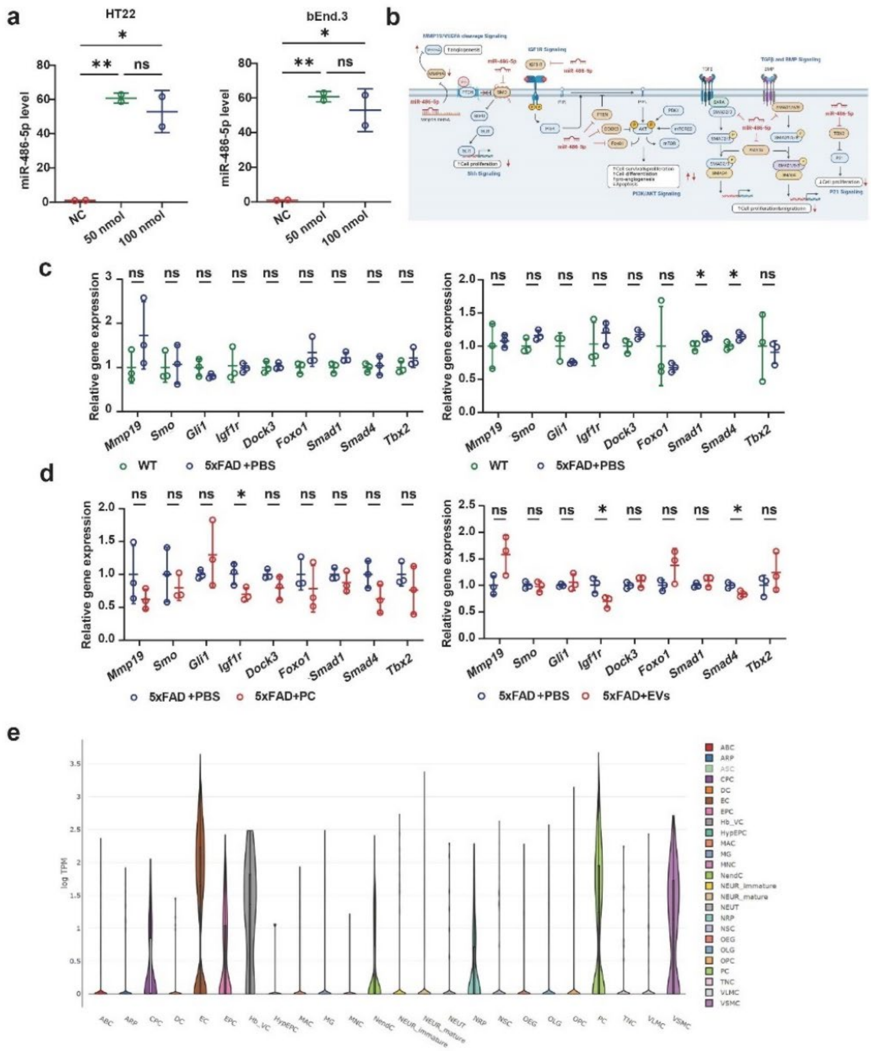

Figure S5. Potential downstream signaling pathways of miR-486-5p.

(a) An amount of 50nmol or 100nmol of miR-486-5P mimics could significantly increase the level of miR-486-5P in HT22 or bEnd.3 cells. (b) We conducted a preliminary exploration of potential downstream signaling pathways. Through sequencing and literature review, we identified miR-486-5p-related signaling pathways. (c–d) Further RT-PCR analysis of various molecules within these pathways revealed significant changes in type 1 insulin-like growth factor receptor (*IGF1R*), indicating that this molecule may be a critical downstream target. (e) *Igf1r* was found to be highly expressed in neurons and endothelial cells in mouse brain according to the published database (https://singlecell.broadinstitute.org/single_cell/study/SCP263/aging-mouse-brain) .

(a, c, d) Data are presented as the mean ± S.E. The experiment had no less than three independent biological replicates (One-way ANOVA). ns, $P \geq 0.05$; *, $P<0.05$; **, $P<0.01$.

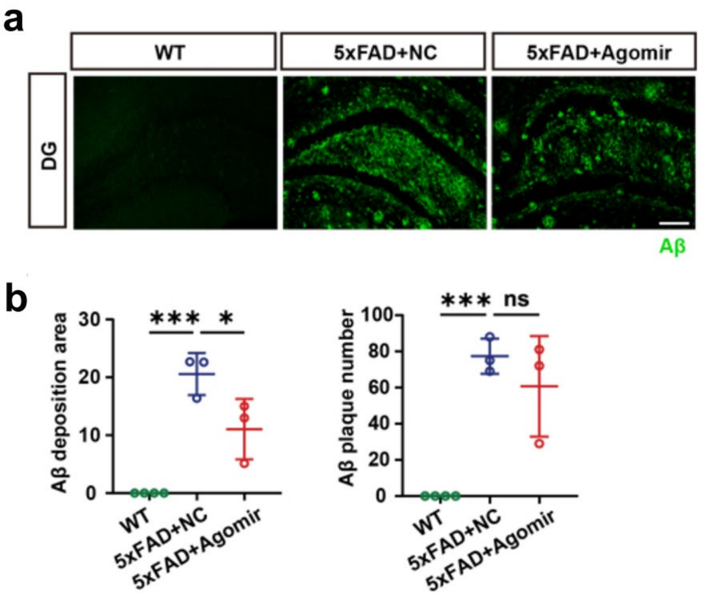

Figure S6. The miR-486-5P treatment decreased the Aβ deposition in DG area.

(a) Representative fluorescence photomicrographs of Aβ in the hippocampal DG area of WT, 5XFAD, and 5XFAD mice with EV transplantation. (b) Immunofluorescence staining revealed that Aβ deposition in the hippocampal DG region of 5xFAD mice decreased post agomir injection.

(b) Data are presented as the mean ± S.E. The experiment had no less than three independent biological replicates (One-way ANOVA). ns, $P \geq 0.05$; *, $P<0.05$; **, $P<0.01$.

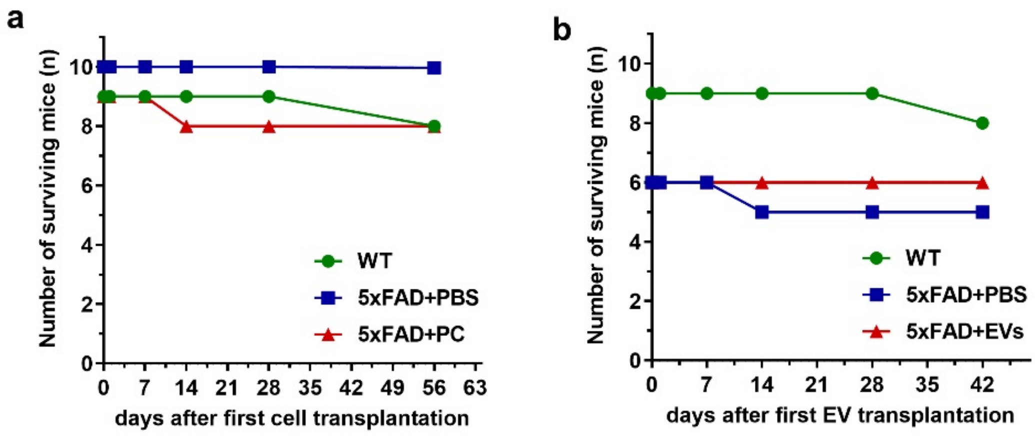

Figure S7. The number of surviving mice at different time points after cell or EV injection.

(a) The number of surviving mice at different time points after cell injection. (b) The number of surviving mice at different time points after EV injection.

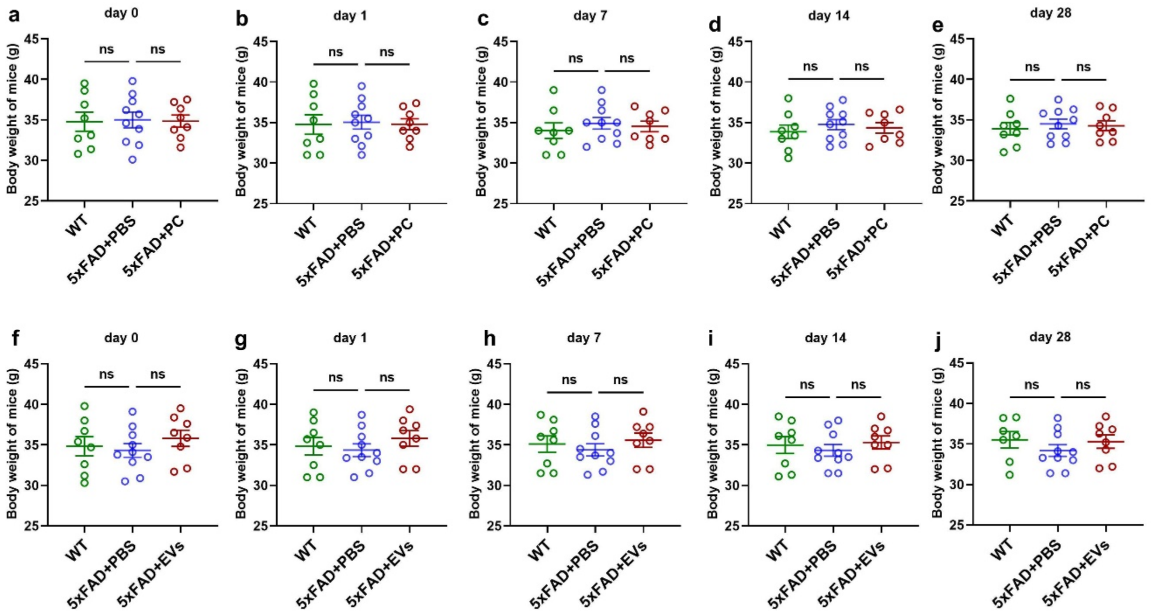

Figure.S8. Body weight of mice in each group at different time points.

Body weight of mice in each group on the day of injection (d0), as well as 1-, 7-, 14-, and 28-days post-injection. No significant decrease in body weight was observed at any of the examined time points. Data are presented as the mean ± S.E. The experiment had no less than three independent biological replicates (One-way ANOVA). ns, $P \geq 0.05$.

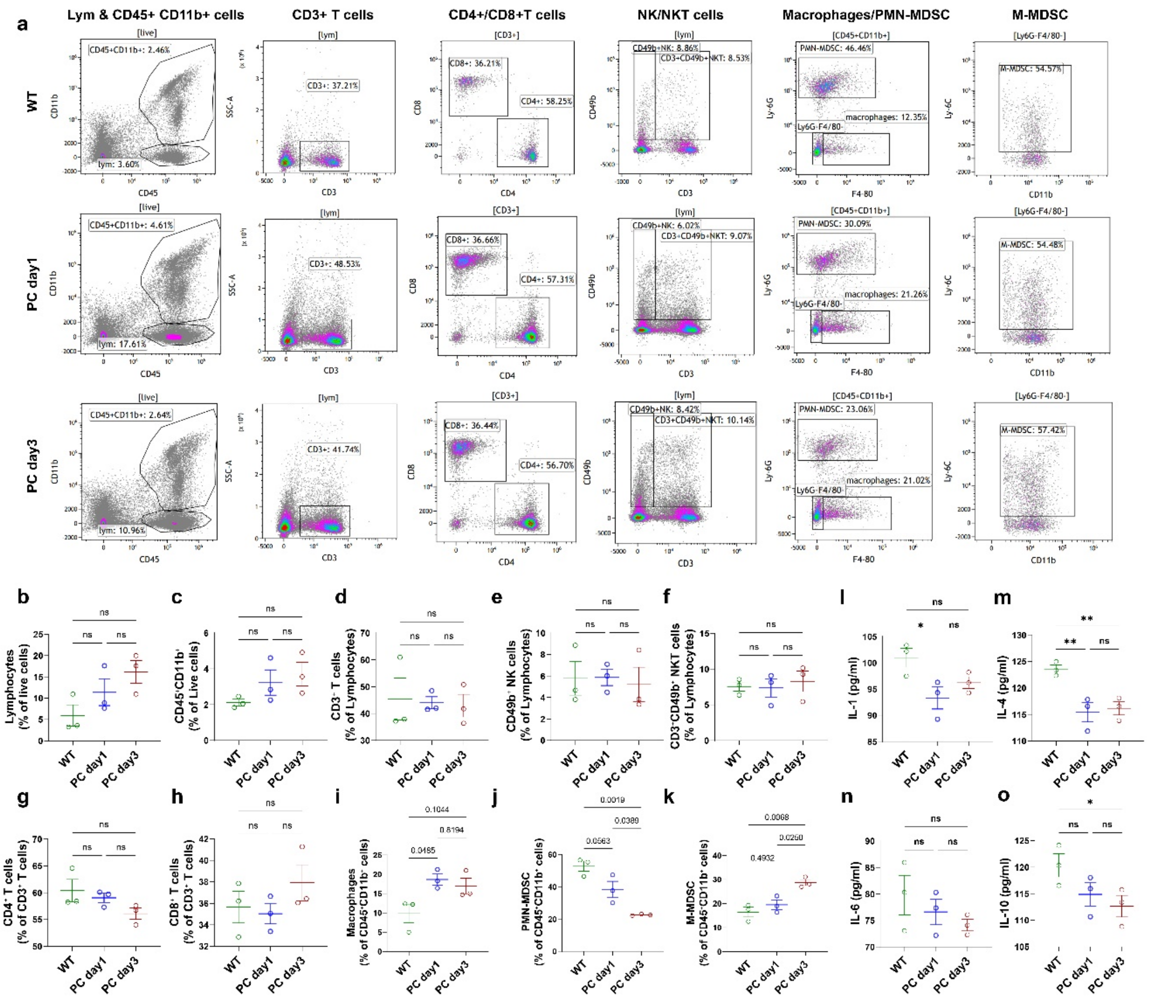

Figure S9. Landscape of peripheral blood immune cell populations and inflammatory cytokines 1 and 3 days after intravenous administration of hPSC-CNC PCs (PC).

(a) Flow cytometric analysis of peripheral blood immune cell dynamics in mice following intravenous injection of PC. Gating strategy and representative dot plots. (b–k) Quantification of immune cell subsets in wild-type mice at 1 and 3 days post-injection: lymphocytes (% of live cells), $CD45^+CD11b^+$ cells (% of live cells), $CD3^+$ T cells (% of lymphocytes), $CD49b^+$ NK cells (% of lymphocytes), $CD3^+CD49b^+$ NKT cells (% of lymphocytes), $CD4^+$ T cells (% of $CD3^+$ T cells), $CD8^+$ T cells (% of $CD3^+$ T cells), macrophages (% of $CD45^+CD11b^+$ cells), PMN-MDSCs (% of $CD45^+CD11b^+$ cells), and M-MDSCs (% of $CD45^+CD11b^+$ cells). (l–o) Serum levels of pro- and anti-inflammatory cytokines IL-1, IL-4, IL-6, and IL-10 measured in the same mice. Data are presented as mean ± SEM (n = 3 per group); statistical significance was determined by one-way ANOVA with Tukey's post hoc test (ns, $P \geq 0.05$; *$P < 0.05$; **$P < 0.01$).

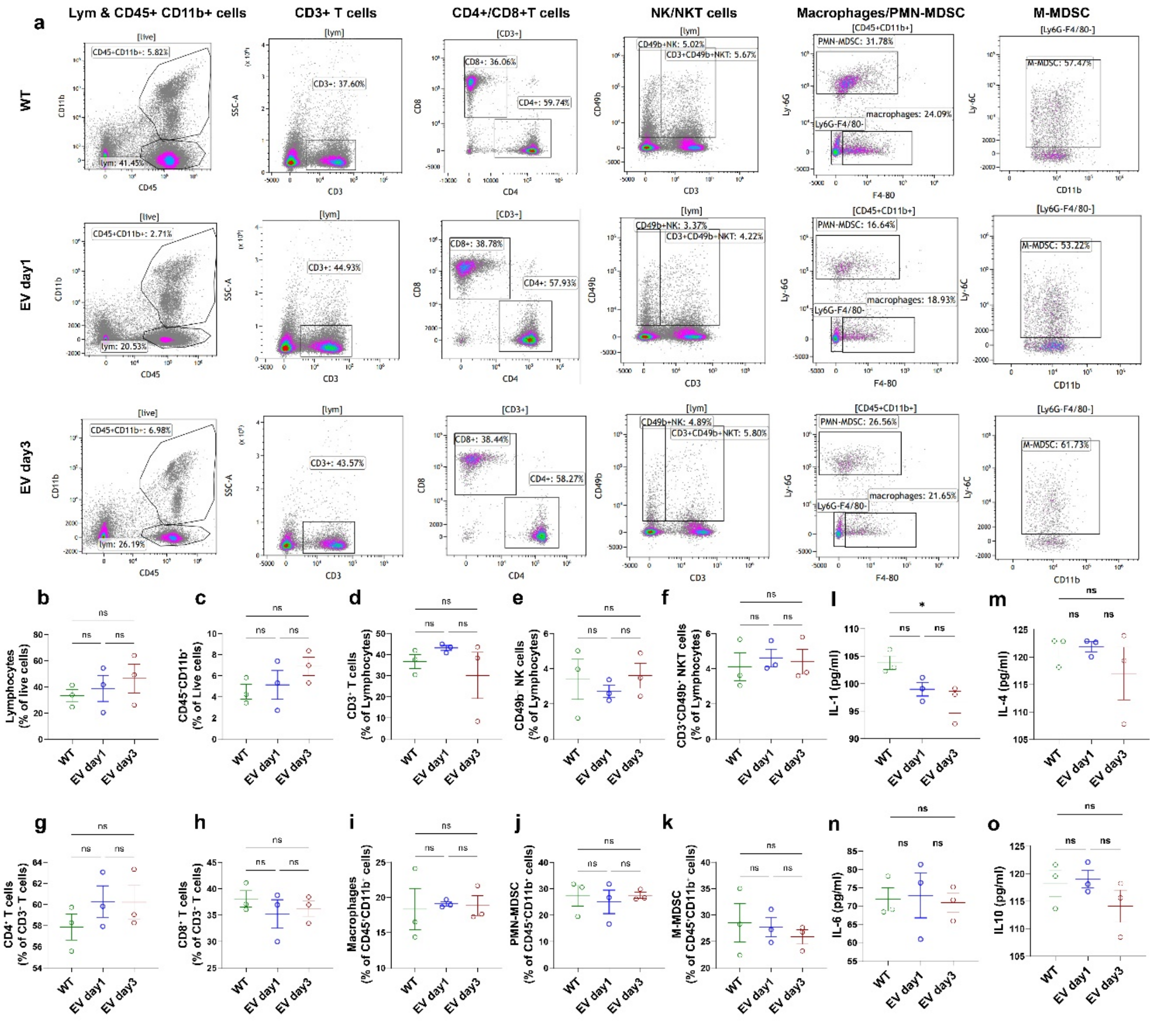

Figure S10. Landscape of peripheral blood immune cell populations and inflammatory cytokines 1 and 3 days after intravenous administration of EVs derived from hPSC-CNC PCs.

(a) Flow cytometric analysis of peripheral blood immune cell dynamics in mice following intravenous injection of EVs. Gating strategy and representative dot plots. (b–k) Quantification of immune cell subsets in wild-type mice at 1 and 3 days post-injection: lymphocytes (% of live cells), $CD45^+CD11b^+$ cells (% of live cells), $CD3^+$ T cells (% of lymphocytes), $CD49b^+$ NK cells (% of lymphocytes), $CD3^+CD49b^+$ NKT cells (% of lymphocytes), $CD4^+$ T cells (% of $CD3^+$ T cells), $CD8^+$ T cells (% of $CD3^+$ T cells), macrophages (% of $CD45^+CD11b^+$ cells), PMN-MDSCs (% of $CD45^+CD11b^+$ cells), and M-MDSCs (% of $CD45^+CD11b^+$ cells). (l–o) Serum levels of pro- and anti-inflammatory cytokines IL-1, IL-4, IL-6, and IL-10 measured in the same mice. Data are presented as mean ± SEM (n = 3 per group); statistical significance was determined by one-way ANOVA with Tukey's post hoc test (ns, $P \geq 0.05$; *$P < 0.05$; **$P < 0.01$).
